# Associations between genetically determined dietary factors and risk of autism spectrum disorder: a Mendelian randomization study

**DOI:** 10.3389/fnut.2024.1210855

**Published:** 2024-03-01

**Authors:** Wenwen Li, Cuncheng Liu, Shouqiang Chen

**Affiliations:** ^1^Second School of Clinical Medicine, Shandong University of Traditional Chinese Medicine, Jinan, China; ^2^Department of Neonatology, Weifang Traditional Chinese Hospital, Weifang, China

**Keywords:** Mendelian randomization, dietary factors, autism spectrum disorder, poultry intake, beef intake, cheese intake, dried fruit intake

## Abstract

**Background:**

Existing studies confirm the importance of dietary factors in developing autism spectrum disorder (ASD) and disease progression. Still, these studies are primarily observational, and their causal relationship is unknown. Moreover, due to the extensive diversity of food types, the existing research remains somewhat limited in comprehensiveness. The inconsistency of the results of some studies is very disruptive to the clinic. This study infers a causal relationship between dietary factors on the risk of developing ASD from a genetic perspective, which may lead to significant low-cost benefits for children with ASD once the specificity of dietary factors interfering with ASD is confirmed.

**Methods:**

We performed a two-sample Mendelian randomization (MR) analysis by selecting single nucleotide polymorphisms (SNPs) for 18 common dietary factors from the genome-wide association study (GWAS) database as instrumental variables (IVs) and obtaining pooled data for ASD (Sample size = 46,351) from the iPSYCH-PGC institution. Inverse variance weighted (IVW) was used as the primary analytical method to estimate causality, Cochran's Q test to assess heterogeneity, the Egger-intercept test to test for pleiotropy and sensitivity analysis to verify the reliability of causal association results.

**Results:**

The MR analysis identified four dietary factors with potential causal relationships: poultry intake (fixed-effects IVW: OR = 0.245, 95% CI: 0.084–0.718, *P* < 0.05), beef intake (fixed-effects IVW: OR = 0.380, 95% CI: 0.165–0.874, *P* < 0.05), cheese intake (random-effects IVW: OR = 1.526, 95% CI: 1.003–2.321, *P* < 0.05), and dried fruit intake (fixed-effects IVW: OR = 2.167, 95% CI: 1.342–3.501, *P* < 0.05). There was no causal relationship between the remaining 14 dietary factors and ASD (*P* > 0.05).

**Conclusion:**

This study revealed potential causal relationships between poultry intake, beef intake, cheese intake, dried fruit intake, and ASD. Poultry and beef intake were associated with a reduced risk of ASD, while cheese and dried fruit intake were associated with an increased risk. Other dietary factors included in this study were not associated with ASD.

## Introduction

Autism spectrum disorder (ASD) is a neurodevelopmental disease characterized by social and communication impairments, restricted interests, and repetitive behavior (1). The global incidence is ~1–3% (2–5), and the incidence of the disease is increasing yearly due to significant changes in the social environment, improved diagnosis and widespread social attention. The high prevalence and highly abnormal social stereotypic behaviors have made it an increasingly important health problem threatening children worldwide. Children with ASD have a variety of presentations that overlap with the clinical manifestations of many psychiatric disorders (1). The inaccuracy of diagnostic methods based on scales and the lack of specific diagnostic markers (6, 7) ultimately lead to an increased likelihood of under diagnosis and misdiagnosis, which is a significant threat to the physical and mental health of children and their growth and development as well as a potential social hazard. Currently, no single treatment is effective for all symptoms of ASD, and routinely prescribed therapy for a primary sign is not recommended (8), with the first line of treatment remaining behavioral interventions (7).

Its etiology and pathogenesis are not clear, but it is generally accepted that dietary factors are one of the critical factors in the development of ASD (3, 9). The gut-brain axis (GBA) effect on ASD provides a theoretical basis for the link between diet and ASD (10–12). The GBA promotes interactions between the gut system and the neuroendocrine, neuroimmune, and autonomic nervous systems, maintains homeostasis in the brain, and helps regulate cognitive and emotional functions (13–15). And the gut microbiome has been shown to play an essential role in regulating GBA (16). Dietary preferences inevitably affect the ecological balance of the gut microbiome and the digestion and absorption of nutrients, resulting in abnormal functional architecture along the GBA associated with ASD phenotypic heterogeneity, affecting the coding of amino acid, carbohydrate, and lipid profiles associated with ASD and changes in gene expression associated with the brain, leading to neurodevelopmental disorders and ASD (10, 17). In addition, a study by SRM Alsubaiei et al. (18) suggested that dietary therapies have anti-inflammatory and antioxidant effects and can improve oxidative stress and neuroinflammation to help prevent ASD. A case-control study that included 38 children and adolescents with ASD and 38 gender and age-matched peers without ASD suggested that maintaining a diet high in antioxidant capacity may effectively reduce some of the symptoms of ASD (19). Several other studies have also shown a strong link between diet and ASD. For example, a study by Zhang et al. (3) found that children with ASD had decreased fruit and vegetable consumption by comparing the eating and mealtime behavioral changes of 105 children with ASD and 105 children with typically developing (TD) and conducted an external validation cohort including 82 children with ASD and 51 TD children, with reliable results. A clinical study involving 106 children with ASD and 207 children with TD by Wang et al. (20) showed that poor diet quality was associated with impaired working memory and organizational capacity in children with ASD and that attention should be paid to improving their dietary quality. Mathew et al. (21) showed that altered dietary intake in children with ASD was associated with differences in autistic traits and sensory processing styles. A study by Rodrigues et al. (22) showed that children with ASD are more selective in their diets than children with TD. Although many studies have found a correlation between dietary factors and ASD, the causal relationship between the two is inconclusive. If risk and protective factors in the diet can be identified, there may be significant low-cost benefits for children with ASD.

Mendelian randomization (MR) is a type of instrumental variables (IVs) analysis that employs genetic variants as IVs to infer causal relationships between exposures and outcomes (23). In MR studies, IVs are genetic variants occurring during meiosis, making them less susceptible to environmental influences and confounding factors. They adhere to the principle of random allocation, akin to a “natural” randomized controlled trial (RCT), which enhances the strength of the causal evidence compared to observational studies (24); meanwhile, genetic variations are established prenatally and persist throughout one's lifetime, thereby enabling MR studies to avoid the influence of reverse causality effectively (25). The widespread application of MR continues to grow, especially with the accumulation of data from genome-wide association studies (GWAS). An increasing number of studies are employing MR methods to investigate causal relationships between dietary factors and diseases. These studies indicate that dietary factors can influence disease risk and lifespan (26–29). Adjusting dietary composition and altering dietary habits can be effective in disease prevention. Therefore, MR is an ideal approach for investigating causal relationships between dietary factors and ASD. This study uses MR methods to uncover the intrinsic link between dietary factors and ASD at the genetic level, hoping to provide research ideas for the prevention and diagnosis of ASD and help improve dietary guidance for children with ASD.

## Methods

### Study design

This study was conducted according to the guidelines of the STROBE-MR statement (30, 31). In this study, dietary factors were used as exposure variables and single nucleotide polymorphisms (SNPs) loci significantly associated with them were selected as IVs, and the outcome variable was ASD. A two-sample MR analysis was performed using a publicly available GWAS based extensive sample database. Cochran's Q test was used to assess heterogeneity, the Egger-intercept test for pleiotropy and sensitivity analysis to verify the reliability of causal association results. As the data used is previously publicly available, no additional ethical approval is required.

**Figure 1:**
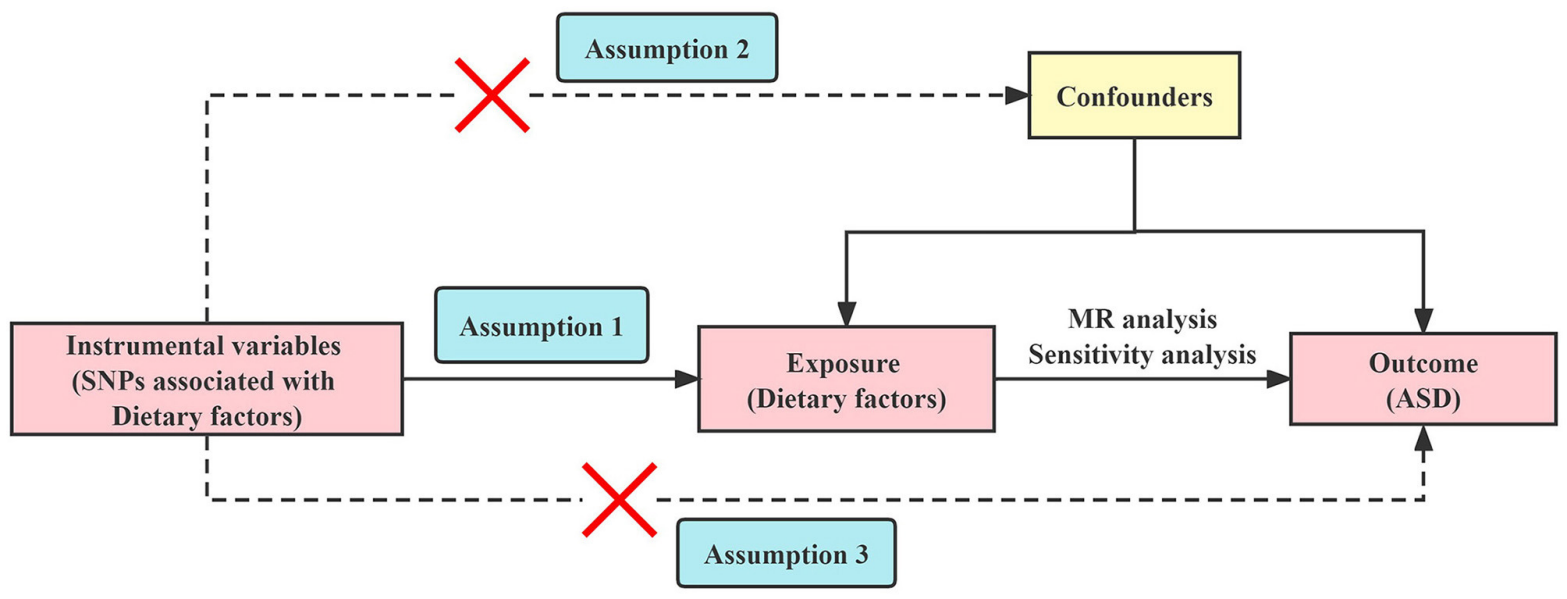
Overview of the Mendelian randomization study design. SNPs, single nucleotide polymorphisms; MR, Mendelian randomization; ASD, autism spectrum disorder.

MR analysis requires three core assumptions to be satisfied (32): (i) The selected IVs are strongly correlated with exposure; (ii) IVs are not related to confounding factors; (iii) IVs cannot be directly related to outcomes. The two-sample MR study design model is shown in Figure 1.

### Data source

Data on exposure variables were obtained from the IEU Open GWAS database summary website (https://gwas.mrcieu.ac.uk/), which primarily comprises publicly available GWAS summary data. We selected 18 variables as exposure factors, including processed meat intake (GWAS ID: ukb-b-6324), beef intake (GWAS ID: ukb-b-2862), pork intake (GWAS ID: ukb-b-5640), lamb/mutton intake (GWAS ID: ukb-b-14179), non-oily fish intake (GWAS ID: ukb-b-17627), oily fish intake (GWAS ID: ukb-b-2209), poultry intake (GWAS ID: ukb-b-8006), cooked vegetable intake (GWAS ID: ukb-b-8089), salad/raw vegetable intake (GWAS ID: ukb-b-1996), water intake (GWAS ID: ukb-b-14898), tea intake (GWAS ID: ukb-b-6066), coffee intake (GWAS ID: ukb-b-5237), alcohol intake frequency (GWAS ID: ukb-b-5779), bread intake (GWAS ID: ukb-b-11348), cheese intake (GWAS ID: ukb-b-1489), cereal intake (GWAS ID: ukb-b-15926), dried fruit intake (GWAS ID: ukb-b-16576), and fresh fruit intake (GWAS ID: ukb-b-3881). These exposure datasets were extracted from the UK Biobank through the IEU Open GWAS project. The summary statistics data for the outcome variable (ASD) was also derived from the IEU Open GWAS database. However, it should be noted that this dataset was not extracted from the UK Biobank (the outcome variables were from a different dataset to the exposed variables). In the IEU Open GWAS database, a search for “autism spectrum disorder” GWAS was conducted, and a dataset was selected based on sample size, originating from Integrative Psychiatric Research and the Psychiatric Genomics Consortium (iPSYCH-PGC) (GWAS ID: ieu-a-1185, https://gwas.mrcieu.ac.uk/datasets/ieu-a-1185/). This dataset comprises 46,351 participants of European ancestry (18,382 cases and 27,969 controls) and 9,112,386 SNPs. Both the exposed and outcome study populations were of European ancestry to mitigate bias stemming from race-related confounding factors. The specific information is shown in Table 1.

**Table 1:** Details of the GWAS included in the two-sample Mendelian randomization study.

**Exposure or outcome**	**GWAS ID**	**Sample size**	**Number of SNPs**	**Consortium**	**Population**	**Year**	**Author**
Processed meat intake	ukb-b-6324	461,981	9,851,867	MRC-IEU	European	2018	Ben Elsworth
Beef intake	ukb-b-2862	461,053	9,851,867	MRC-IEU	European	2018	Ben Elsworth
Pork intake	ukb-b-5640	460,162	9,851,867	MRC-IEU	European	2018	Ben Elsworth
Lamb/mutton intake	ukb-b-14179	460,006	9,851,867	MRC-IEU	European	2018	Ben Elsworth
Non-oily fish intake	ukb-b-17627	460,880	9,851,867	MRC-IEU	European	2018	Ben Elsworth
Oily fish intake	ukb-b-2209	460,443	9,851,867	MRC-IEU	European	2018	Ben Elsworth
Poultry intake	ukb-b-8006	461,900	9,851,867	MRC-IEU	European	2018	Ben Elsworth
Cooked vegetable intake	ukb-b-8089	448,651	9,851,867	MRC-IEU	European	2018	Ben Elsworth
Salad/raw vegetable intake	ukb-b-1996	435,435	9,851,867	MRC-IEU	European	2018	Ben Elsworth
Water intake	ukb-b-14898	427,588	9,851,867	MRC-IEU	European	2018	Ben Elsworth
Tea intake	ukb-b-6066	447,485	9,851,867	MRC-IEU	European	2018	Ben Elsworth
Coffee intake	ukb-b-5237	428,860	9,851,867	MRC-IEU	European	2018	Ben Elsworth
Alcohol intake frequency	ukb-b-5779	462,346	9,851,867	MRC-IEU	European	2018	Ben Elsworth
Bread intake	ukb-b-11348	452,236	9,851,867	MRC-IEU	European	2018	Ben Elsworth
Cheese intake	ukb-b-1489	451,486	9,851,867	MRC-IEU	European	2018	Ben Elsworth
Cereal intake	ukb-b-15926	441,640	9,851,867	MRC-IEU	European	2018	Ben Elsworth
Dried fruit intake	ukb-b-16576	421,764	9,851,867	MRC-IEU	European	2018	Ben Elsworth
Fresh fruit intake	ukb-b-3881	446,462	9,851,867	MRC-IEU	European	2018	Ben Elsworth
Autism spectrum disorder	ieu-a-1185	46,351	9,112,386	iPSYCH-PGC	European	2017	-

### Selection of instrumental variables

SNPs that were significantly correlated with exposure factors were screened (*P* < 5.0 × 10^−8^), and SNPs in a state of linkage disequilibrium (LD) were removed using a strict cut-off (*r*^2^ < 0.001, region size = 10000 kb). The bias introduced by weak IVs was avoided by excluding IVs with *F* ≤ 10 (33, 34). SNPs associated with confounders and outcomes were removed via the PhenoScanner website (http://www.phenoscanner.medschl.cam.ac.uk/) (35). We harmonized exposure and outcome SNP effects and excluded palindromic and incompatible SNPs (36). MR-pleiotropy residual sum outlier (MR-PRESSO) test detected and excluded horizontal pleiotropy outliers (37). The final SNPs obtained by filtering according to the above criteria were used for MR analysis.

### Statistical analysis

All analyses were performed using the “TwoSampleMR” (version 0.5.6) and “MR-PRESSO” packages in R software (version 4.2.1) (38). This study used inverse variance weighted (IVW) (39) as the primary analysis method to infer a potential causal relationship between dietary factors and ASD. IVW is based on the assumption that all genetically variable SNPs are valid IVs with an overall bias of zero and is the most common and accurate method for detecting causality in MR analysis. However, MR-Egger intercept analysis (40) must satisfy *P* > 0.05, i.e., no horizontal pleiotropy is present; otherwise, the IVW results are unreliable. Heterogeneity was assessed according to Cochran's Q test: if *P* < 0.05, heterogeneity was present, random-effects IVW was selected, when heterogeneity was acceptable; if *P* ≥ 0.05, heterogeneity was not present, fixed-effects IVW was selected. The weighted median method (41) and MR-Egger regression (42) complement IVW for MR analysis. The weighted median method informs the majority of evidence-supported estimates based on the assumption that more than 50% of the weights are derived from valid genetic instruments. MR-Egger regression allows all genetic instruments to be pleiotropic, thereby providing consistent estimates, provided that the InSIDE assumptions are met (43). The leave-one-out analysis is used for sensitivity analysis to assess the stability of the results.

## Results

After screening the corresponding GWAS databases for SNPs with strong correlations with exposure and eliminating the interference of LD, the number of SNPs we initially screened for the 18 exposure factors ranged from 8 to 99. The number of SNPs with F ≤ 10 was excluded, ranging from 0 to 19, and the remaining SNPs all had F statistics above 10. After completing all screening criteria, the final number of valid SNPs for MR analysis ranged from 6 to 69. Basic information about these SNPs is presented in the Supplementary material (Basic information of SNPs).

**Figure 2:**
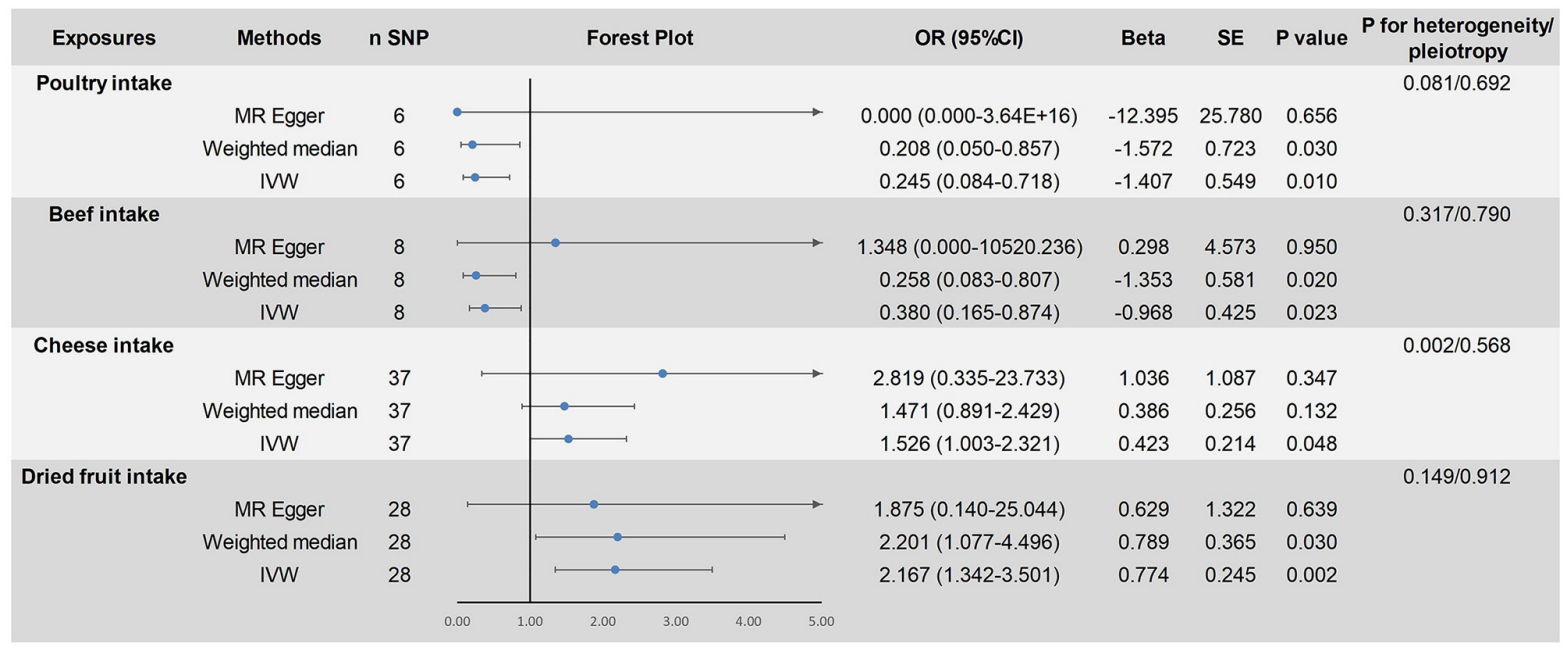
Mendelian randomized causal estimation of the risk of autism spectrum disorder by poultry intake, cheese intake, and dried fruit intake.

This study analyzed the causal relationship between 18 dietary factors and ASD. Based on the results of the IVW method, a causal relationship between four dietary factors and ASD was identified. We found that poultry intake (fixed-effects IVW: OR = 0.245, 95% CI: 0.084–0.718, *P* < 0.05) was associated with a reduced risk of developing ASD and was a protective factor for ASD. The risk of developing an ASD decreases with increased poultry intake. The results of this study were not affected by heterogeneity (*P* > 0.05) or pleiotropy (*P* > 0.05). After removing each SNP individually, the leave-one-out analysis showed no significant bias in our results. The weighted median method (OR = 0.208, 95% CI: 0.050–0.857, *P* < 0.05) verified this finding. In the MR analysis of the beef intake, the fixed-effects IVW results showed an OR = 0.513, 95% CI: 0.231–1.142, *P* = 0.102 > 0.05, indicating no causal relationship between beef intake and the risk of ASD. However, the weighted median method (OR = 0.270, 95% CI: 0.090–0.850, *P* = 0.026 < 0.05) showed a causal relationship. When sensitivity analysis was performed on them, it was found that rs7791463 caused a significant bias to our findings, dominating the occurrence of no statistical significance. The significant estimates observed in the weighted median method also suggest that the potential outliers rs7791463 biased the causal inference of the IVW method (44). We re-ran the MR analysis after excluding rs7791463, and the fixed-effects IVW results showed: OR = 0.380, 95% CI: 0.165–0.874, *P* = 0.023 < 0.05. This result was not affected by heterogeneity (*P* > 0.05) or pleiotropy (*P* > 0.05). No significant bias was found in the leave-one-out analysis. The weighted median method (OR = 0.258, 95% CI: 0.083–0.807, *P* < 0.05) yielded stable results and continues to support that beef intake is a protective factor for ASD. We also identified two risk factors for ASD: cheese intake (random-effects IVW: OR = 1.526, 95% CI: 1.003–2.321, *P* < 0.05) and dried fruit intake (fixed-effects IVW: OR = 2.167. 95% CI: 1.342–3.501, *P* < 0.05). The former pleiotropy was not significant (*P* > 0.05), but there was heterogeneity (*P* < 0.05), and we used random-effects IVW for causal estimation, and the results remained reliable. The latter was not affected by heterogeneity (*P* > 0.05) or pleiotropy (*P* > 0.05). The leave-one-out analysis showed that the results of our study were reliable. The weighted median method verified a causal relationship between dried fruit intake and ASD (OR = 2.201, 95% CI: 1.077–4.496, *P* < 0.05). However, the weighted median method did not find a causal relationship between cheese intake and ASD (*P* > 0.05). Since the three statistical methods yielded the same beta direction and no outliers were found in the sensitivity analysis, the IVW test still prevailed. The causal relationship between cheese intake and ASD remained reliable. None of the results were significant in the MR-Egger regression model (*P* > 0.05). As MR-Egger regression allows all SNPs to be pleiotropic and the findings are not sufficiently rigorous, we do not use MR-Egger regression as the final evaluation criterion for our findings. The results of the above study are shown in Figure 2. The visualization results and sensitivity analysis of the MR analysis can be found in the Supplementary material (Visualization results).

**Figure 3:**
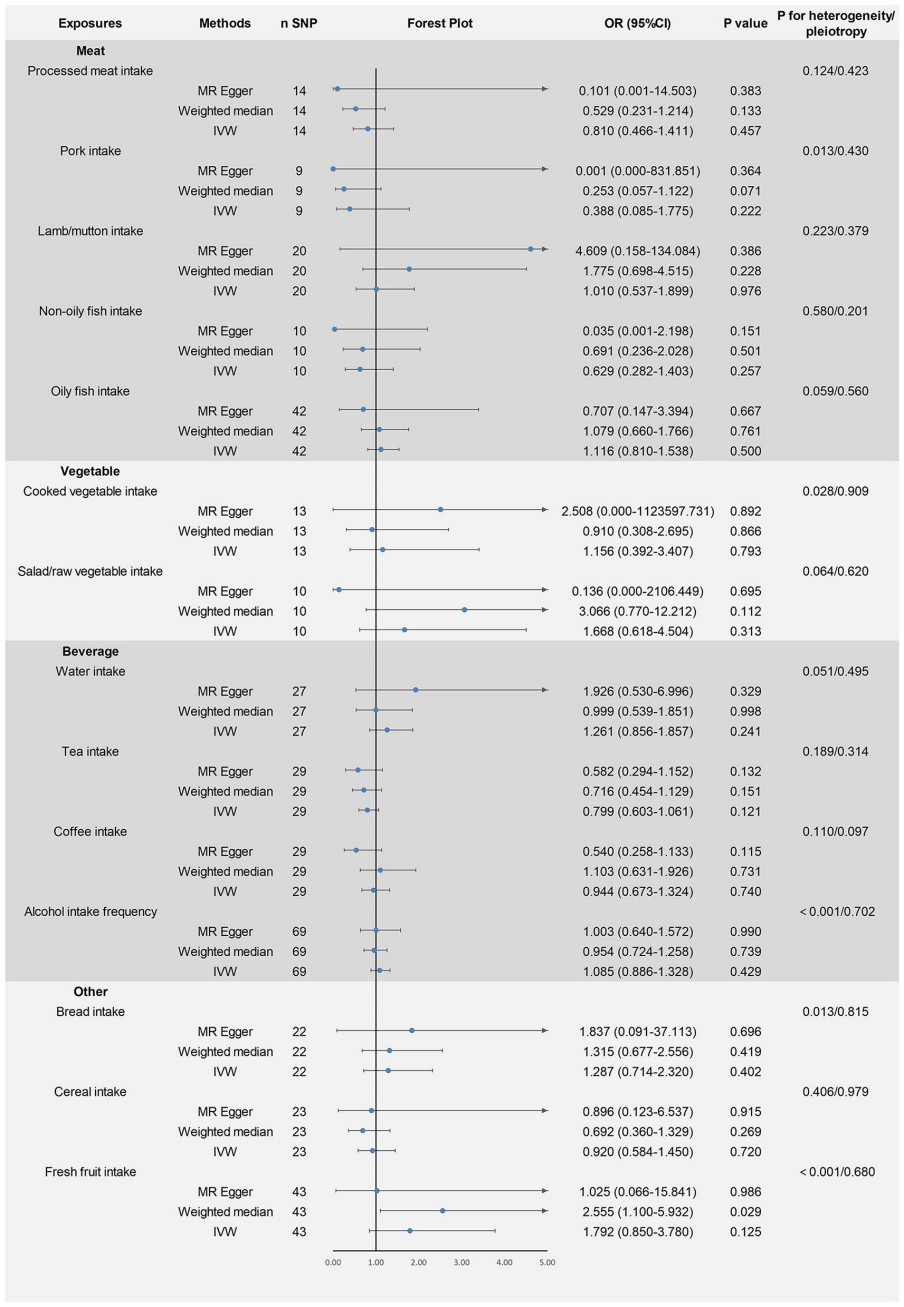
Mendelian randomized causal estimates of 14 dietary factors on the risk of developing autism spectrum disorder.

The remaining 14 dietary factors were tested for causal relationships with ASD using the IVW method, showing that processed meat intake (fixed-effects IVW: OR = 0.810, 95% CI: 0.466–1.411, *P* > 0.05), pork intake (random-effects IVW: OR = 0.388, 95% CI: 0.085–1.775, *P* > 0.05), lamb/mutton intake (fixed-effects IVW: OR = 1.010, 95% CI: 0.537–1.899, *P* > 0.05), non-oily fish intake (fixed-effects IVW: OR = 0.629, 95% CI: 0.282–1.403, *P* > 0.05), oily fish intake (fixed-effects IVW: OR = 1.116, 95% CI: 0.810–1.538, *P* > 0.05), cooked vegetable intake (random-effects IVW: OR = 1.156, 95% CI: 0.392–3.407, *P* > 0.05), salad/raw vegetable intake (fixed-effects IVW: OR = 1.668, 95% CI: 0.618–4.504, *P* > 0.05), water intake (fixed-effects IVW: OR = 1.261, 95% CI: 0.856–1.857, *P* > 0.05), tea intake (fixed-effects IVW: OR = 0.799, 95% CI: 0.603–1.061, *P* > 0.05), coffee intake (fixed-effects IVW: OR = 0.944, 95% CI: 0.673–1.324, *P* > 0.05), alcohol intake frequency (random-effects IVW: OR = 1.085, 95% CI: 0.886–1.328, *P* > 0.05), bread intake (random-effects IVW: OR = 1.287, 95% CI: 0.714–2.320, *P* > 0.05), cereal intake (fixed-effects IVW: OR = 0.920, 95% CI: 0.584–1.450, *P* > 0.05), fresh fruit intake (random-effects IVW: OR = 1.792, 95% CI: 0.850–3.780, *P* > 0.05) and ASD were not causally related. Heterogeneity was found for some of the exposure factors (pork intake, cooked vegetable intake, alcohol intake frequency, bread intake, and fresh fruit intake) and analyses using the random-effects IVW model considered the effect of heterogeneity on the results, which remained reliable. No pleiotropy was found for all results of the MR-Egger intercept analysis test (*P* > 0.05). After excluding each SNP individually, the leave-one-out analysis showed no significant bias in the study results. The results of the above study are shown in Figure 3. The visualization results and sensitivity analysis of the MR analysis can be found in the Supplementary material (Visualization results).

## Discussion

The development of ASD is now understood to be closely associated with genetic factors. However, it is typically not attributed to specific genes or highly localized lesions but rather arises from a combination of unknown genetic risks and abnormalities in neural pathways (45). To date, there are no official laboratory diagnoses or predictive tools for ASD. The forefront of medical research in ASD continues to focus on pathogenesis and diagnostic tools, including genetics, peptides, proteins, metabolites, and transcriptomics. Nevertheless, identifying genetic variations within common risk factors for ASD, which often overlap with the general population, can be challenging (46). In addition to genetic factors, the risk of developing ASD is also strongly associated with dietary factors. Dietary factors play an essential role in the development of ASD, from the maternal nutritional status to the onset and prognosis of children with ASD. Maternal nutritional status may have an impact on fetal brain development. Several studies have suggested that poor nutrition during pregnancy, deficiencies in critical nutrients, and exposure to specific illness may be associated with an increased risk of ASD (47–52). Likewise, infancy and early childhood play a pivotal role in the complex process of brain development. Diet has a profound effect on neurological development and functioning during this critical period, and nutritional imbalances and disorders in the diet may potentially impact the onset and development of ASD. Consequently, extensive research efforts have been dedicated to investigating diet-nutrition therapy for enhancing ASD management. Shaaban et al. (53) conducted an experiment in which probiotic nutritional supplements were administered to autistic children. After 3 months, an examination of fecal specimens revealed increased levels of Bifidobacteria and Lactobacilli, accompanied by significant improvements in autistic severity and gastrointestinal symptoms among the affected children (53). N, N-dimethylglycine (DMG) is a dietary supplement that has been reported to be beneficial for children with ASD, as it can ameliorate the mental and physical conditions of ASD children (54). Additionally, meta-analysis results indicate that dietary interventions can significantly ameliorate the core symptoms of ASD, and gluten-free diets are conducive to improving social behaviors (55). Although many studies have reported the efficacy of dietary interventions for ASD, these studies are predominantly observational in nature, and their causal relationships remain unknown. Moreover, the inconsistency of some research findings poses significant challenges to clinical interpretation. This study gives a genetic perspective on the intrinsic link between diet and the risk associated with ASD.

In this study, 18 daily dietary intake factors were selected as exposure variables, with ASD as the outcome variable. MR methods were employed to infer the causal relationships between dietary intake and ASD. The results indicated that the risk of ASD increased with higher cheese and dried fruit intake, while it decreased with increased beef and poultry intake. However, there were no statistically significant causal relationships observed between ASD and processed meat intake, pork intake, lamb/mutton intake, non-oily fish intake, oily fish intake, cooked vegetable intake, salad/raw vegetable intake, water intake, tea intake, coffee intake, alcohol intake frequency, bread intake, cereal intake, or fresh fruit intake.

A study involving 41 primary school children with ASD and 191 children with TD found that primary school children with ASD consumed fewer cheese and yogurt compared to children with TD (56). This finding contradicts our research results and may be attributed to the smaller sample size included in the study. Most importantly, we cannot determine the temporal relationship between cheese intake and the onset of ASD, and therefore, the relationship between cheese intake and ASD remains worthy of exploration. Current research suggests that cheese contains a high level of casein, and an excess of casein can be metabolized into opioid peptides (57, 58), which mimic analgesic effects and may contribute to the development of certain aberrant neurological behaviors (59, 60). Reducing casein intake may potentially reduce the incidence of ASD (61–63). Some studies have indicated significant improvement in ASD symptoms following strict dietary restrictions of gluten-free and casein-free foods in children (64). Therefore, we posit that cheese intake is highly likely to be a risk factor for ASD. No other studies have indicated a direct relationship between dried fruit and ASD. This study may represent the inaugural attempt to reveal a causal relationship between the two through MR methodology. However, the underlying mechanisms between the two remain unclear. Dried fruits emerge from moisture removal from fruits, culminating in the concentration of nutritional constituents and a relatively heightened sugar content. Research indicates that diets with excessive sugar content might exert unfavorable effects on children with ASD, potentially leading to challenges in emotional and behavioral problems among individuals with autism (65, 66). Given the established positive correlation between inflammation and psychological issues and autism-related symptoms (67, 68), its plausible mechanisms may be associated with the potential elevation of inflammatory risks attributed to sugar intake (66, 69, 70). This could be one factor contributing to the onset of ASD due to excessive consumption of dried fruits. Furthermore, it is worth noting that while the IVW method did not unveil a causal relationship between fresh fruit intake and ASD, the outcomes derived from the weighted median method suggest an elevated risk of ASD with increased intake of fresh fruit. However, in the case of dried fruits after moisture extraction, both research methodologies elucidate a causal relationship with ASD. Future research endeavors should investigate the association between dried fruit intake and the risk of developing ASD. The causal relationship between beef intake and the onset of ASD necessitates further in-depth clinical investigation. However, the underlying connection between these two factors provides us with some basis for investigation. Psychiatric disorders such as autism are genetically correlated with human temperament phenotypes (71, 72). The study by R Costilla et al. (73) showed that genetic control of temperament might be shared between humans and beef cattle. And certain susceptibility genes for ASD are associated with beef cattle temperament. Furthermore, genes associated with the temperament of beef cattle contribute to neuron development functions and exhibit differential expression in human brain tissues. This may provide potential evidence for beef intake as a protective factor in the development of ASD. The protective mechanism of poultry intake as a protective factor for ASD is unclear. It is speculated that it may be related to the intake of micronutrients and unsaturated fatty acids (74–76).

This study has several limitations that warrant consideration. Firstly, the study population consisted of individuals of European ancestry, which may limit the generalizability of the findings to other populations. Secondly, the potential for unobserved pleiotropy, which was not fully addressed in the MR analysis, introduces the possibility of bias in the results. Thirdly, we could not further categorize distinct dietary intake types, nor could we discern the effects of different dietary combinations. Furthermore, the dietary factors selected in this study lack substantiation from foundational research and high-quality, large-scale clinical RCTs regarding their association with ASD. Thus, their effectiveness remains to be confirmed.

## Conclusion

In summary, this study revealed potential causal relationships between poultry intake, beef intake, cheese intake, dried fruit intake, and ASD. Poultry and beef intake were associated with a reduced risk of ASD, while cheese and dried fruit intake were associated with an increased risk. Other dietary factors included in this study were not associated with ASD. These findings provide a foundation for reliable clinical nutritional interventions for children with ASD, contributing to primary prevention strategies for ASD.

## Data availability statement

The original contributions presented in the study are included in the article/Supplementary material, further inquiries can be directed to the corresponding author.

## Author contributions

WL and SC designed the study, analyzed the data, interpreted the results, and wrote the manuscript. WL and CL performed the acquisition, screening of the data, and use of the software. SC provided the foundation and support. All authors contributed to the article and approved the submitted version.
