# Supplementary material for: Associations between genetically determined dietary factors and risk of autism spectrum disorder: a Mendelian randomization study
Source: Front Nutr. 2024 Mar 1;11:1210855. doi: 10.3389/fnut.2024.1210855 (PMC10940521; doi:10.3389/fnut.2024.1210855)
Supplement: Supplementary file 2 [file Data_Sheet_2.PDF]

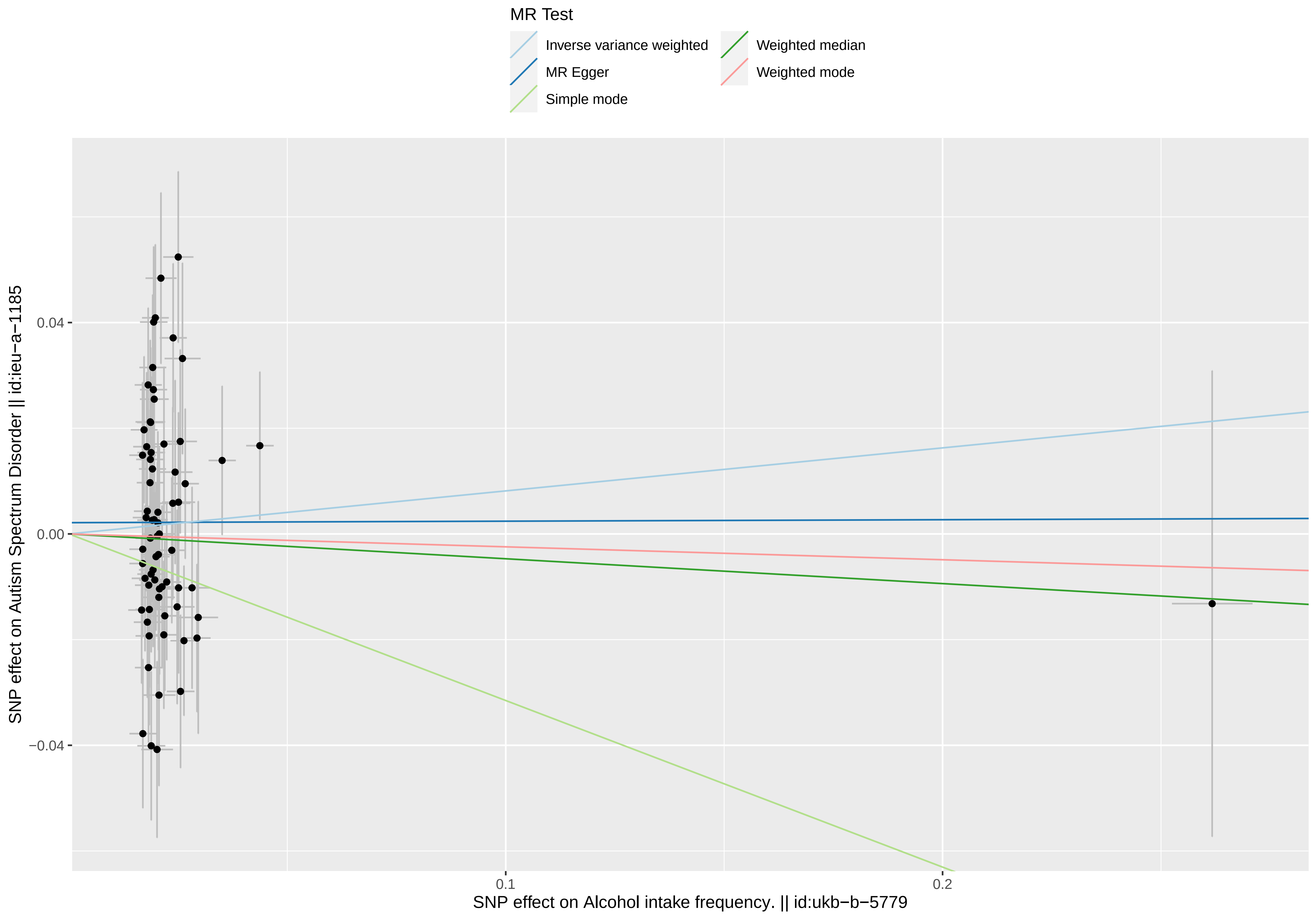
MR Test
Inverse variance weighted
MR Egger
Simple mode
Weighted median
Weighted mode
0.04
0.00
−0.04
0.1
0.2
SNP effect on Autism Spectrum Disorder || id:ieu−a−1185
SNP effect on Alcohol intake frequency. || id:ukb−b−5779

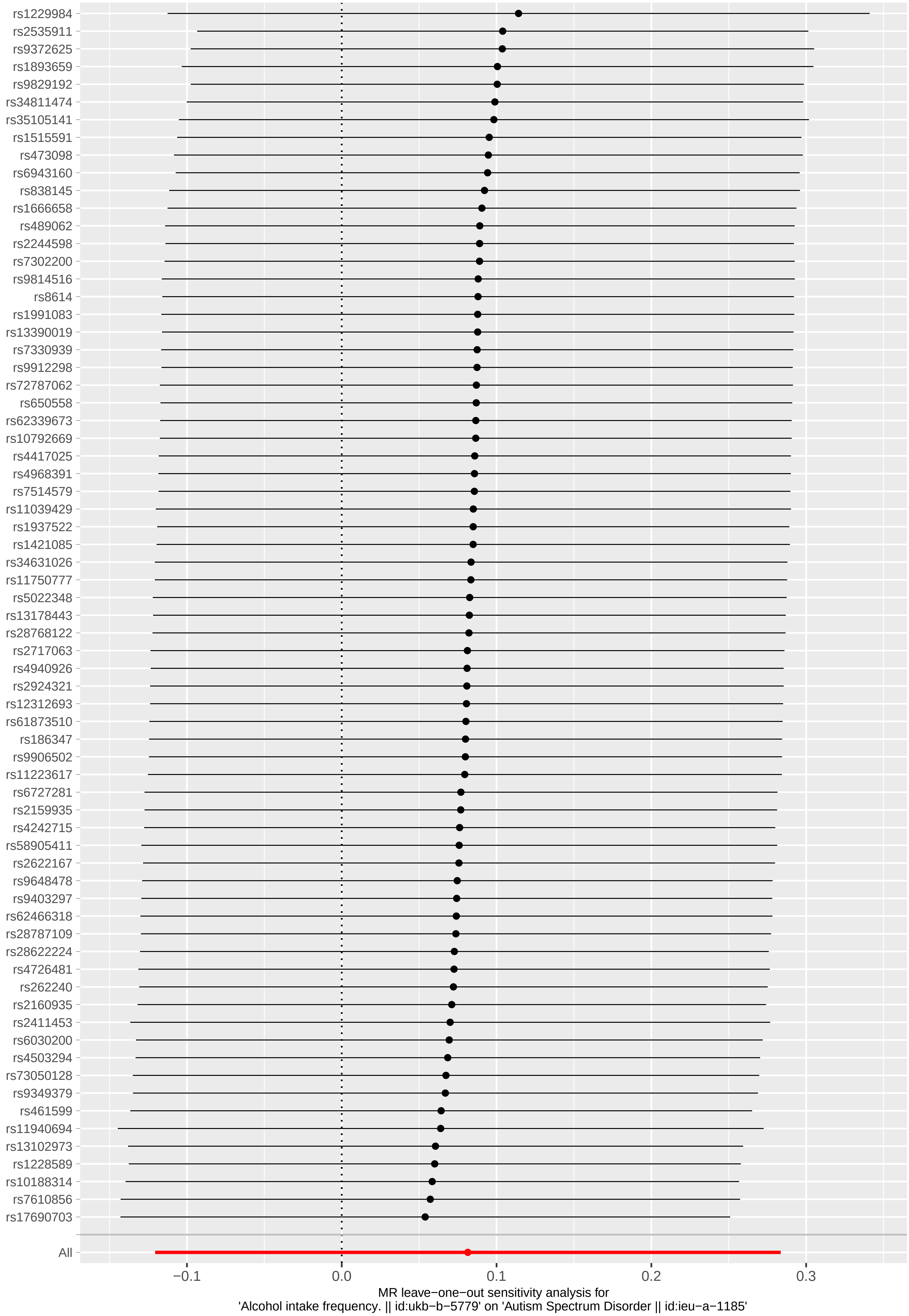
rs1229984
rs2535911
rs9372625
rs1893659
rs9829192
rs34811474
rs35105141
rs1515591
rs473098
rs6943160
rs838145
rs1666658
rs489062
rs2244598
rs7302200
rs9814516
rs8614
rs1991083
rs13390019
rs7330939
rs9912298
rs72787062
rs650558
rs62339673
rs10792669
rs4417025
rs4968391
rs7514579
rs11039429
rs1937522
rs1421085
rs34631026
rs11750777
rs5022348
rs13178443
rs28768122
rs2717063
rs4940926
rs2924321
rs12312693
rs61873510
rs186347
rs9906502
rs11223617
rs6727281
rs2159935
rs4242715
rs58905411
rs2622167
rs9648478
rs9403297
rs62466318
rs28787109
rs28622224
rs4726481
rs262240
rs2160935
rs2411453
rs6030200
rs4503294
rs73050128
rs9349379
rs461599
rs11940694
rs13102973
rs1228589
rs10188314
rs7610856
rs17690703
All
−0.1
0.0
0.1
0.2
0.3
MR leave−one−out sensitivity analysis for
'Alcohol intake frequency. || id:ukb−b−5779' on 'Autism Spectrum Disorder || id:ieu−a−1185'

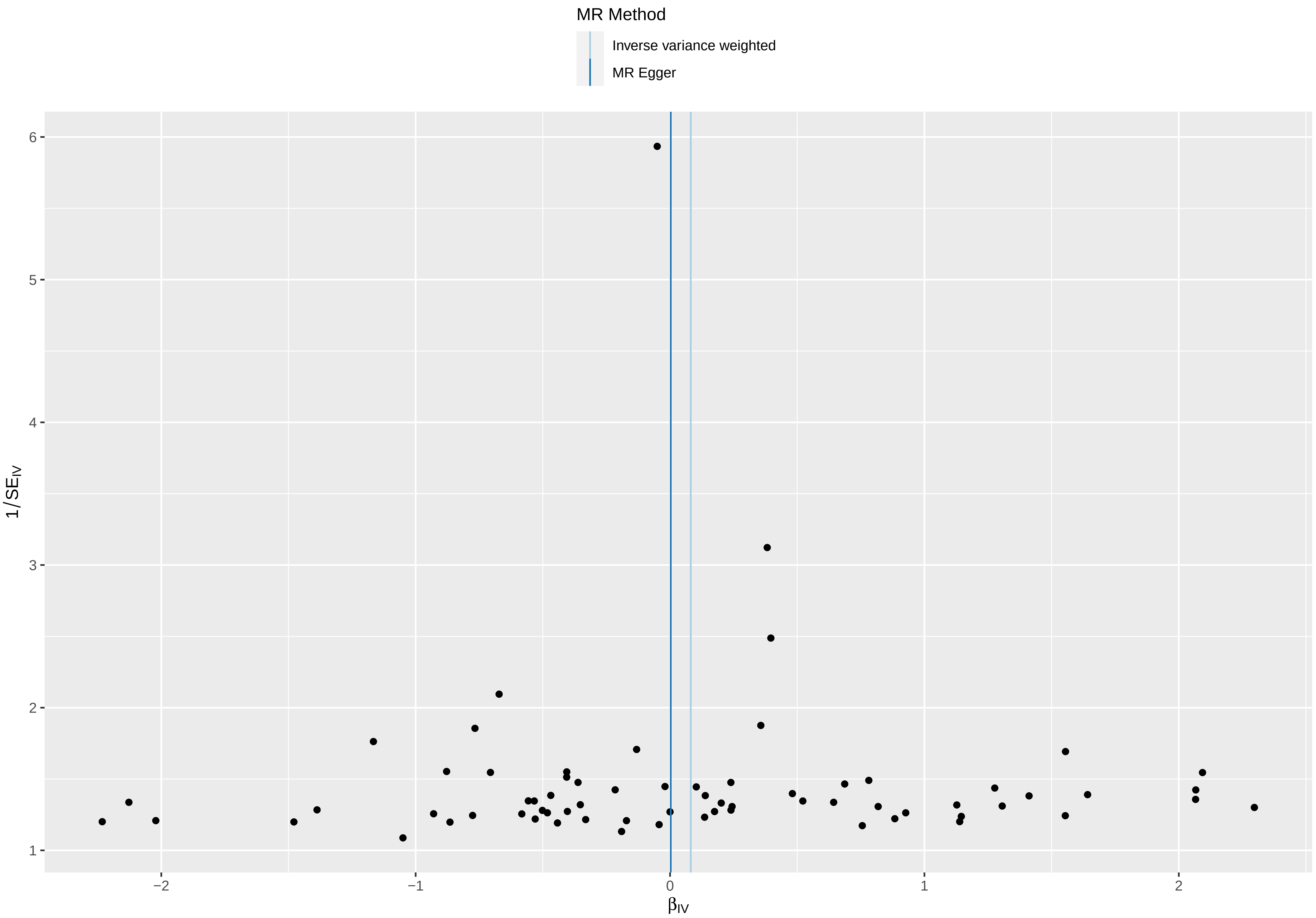
MR Method
Inverse variance weighted
MR Egger
1/SE_{IV}
6
5
4
3
2
1
−2
−1
0
1
2
β_{IV}

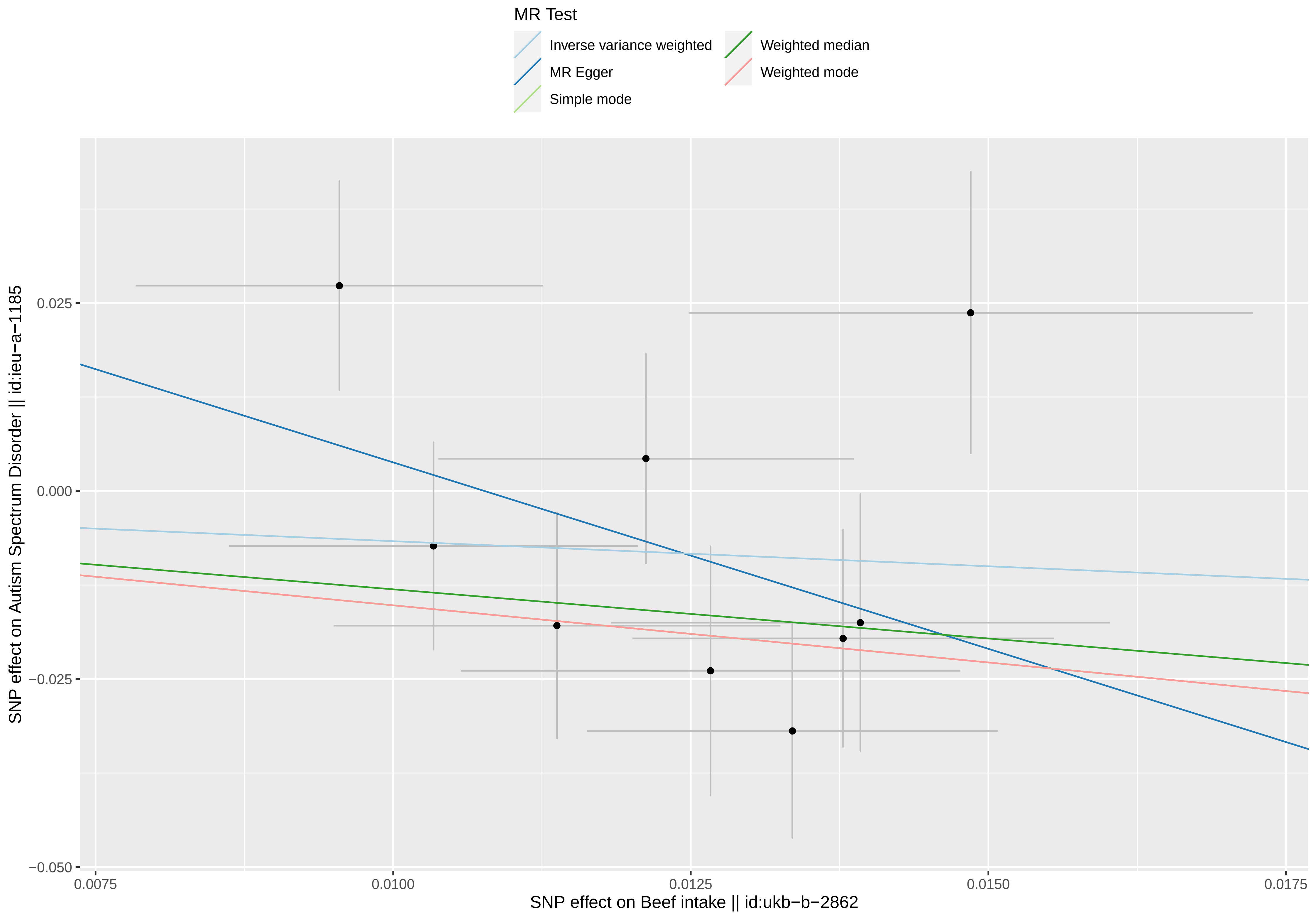
MR Test
Inverse variance weighted
MR Egger
Simple mode
Weighted median
Weighted mode
SNP effect on Autism Spectrum Disorder || id:ieu−a−1185
0.025
0.000
−0.025
−0.050
0.0075
0.0100
0.0125
0.0150
0.0175
SNP effect on Beef intake || id:ukb−b−2862

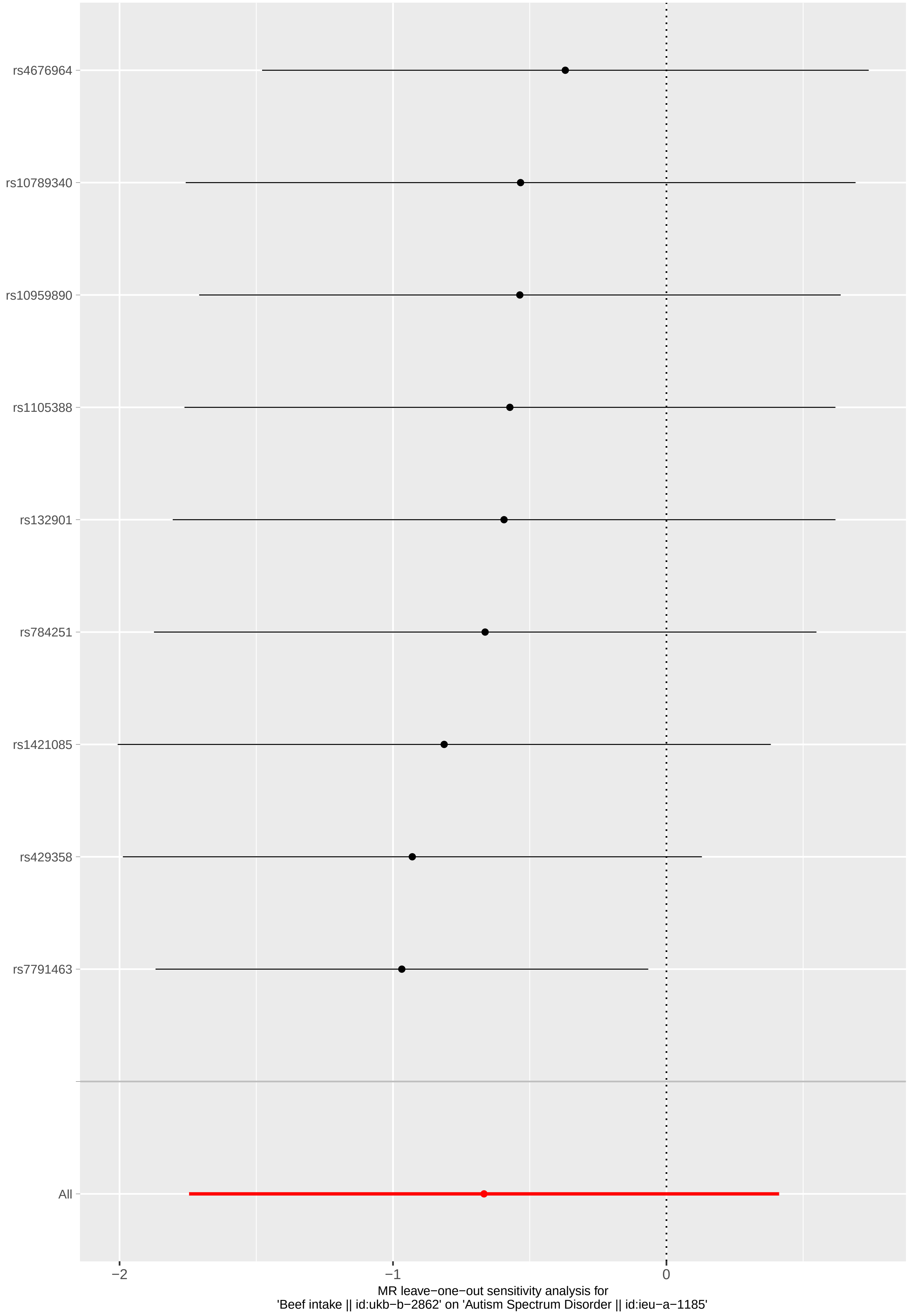
rs4676964
rs10789340
rs10959890
rs1105388
rs132901
rs784251
rs1421085
rs429358
rs7791463
All
−2
−1
0
MR leave−one−out sensitivity analysis for
'Beef intake || id:ukb−b−2862' on 'Autism Spectrum Disorder || id:ieu−a−1185'

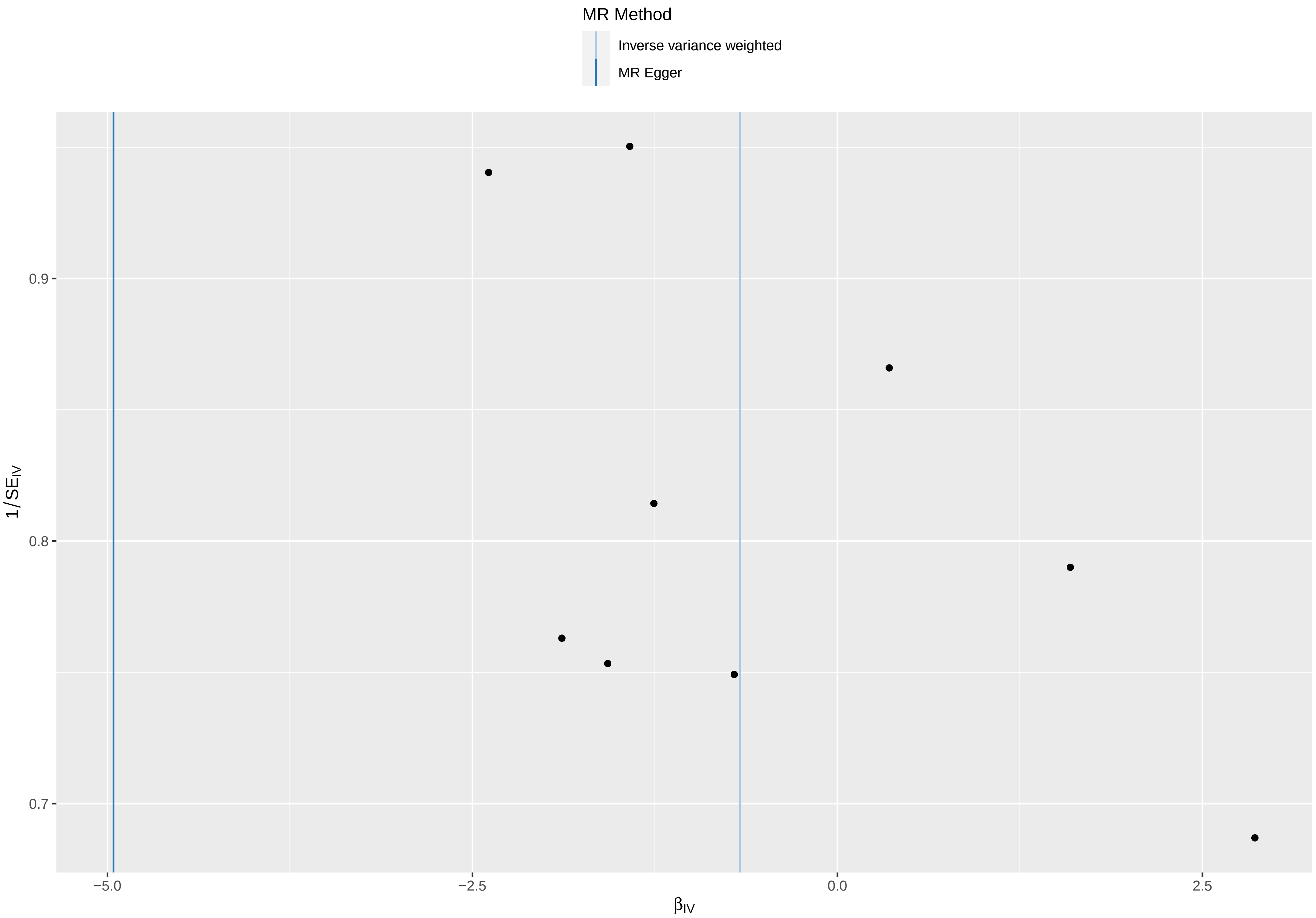
MR Method
Inverse variance weighted
MR Egger
0.9
0.8
0.7
1/SE_IV
−5.0
−2.5
0.0
2.5
β_IV

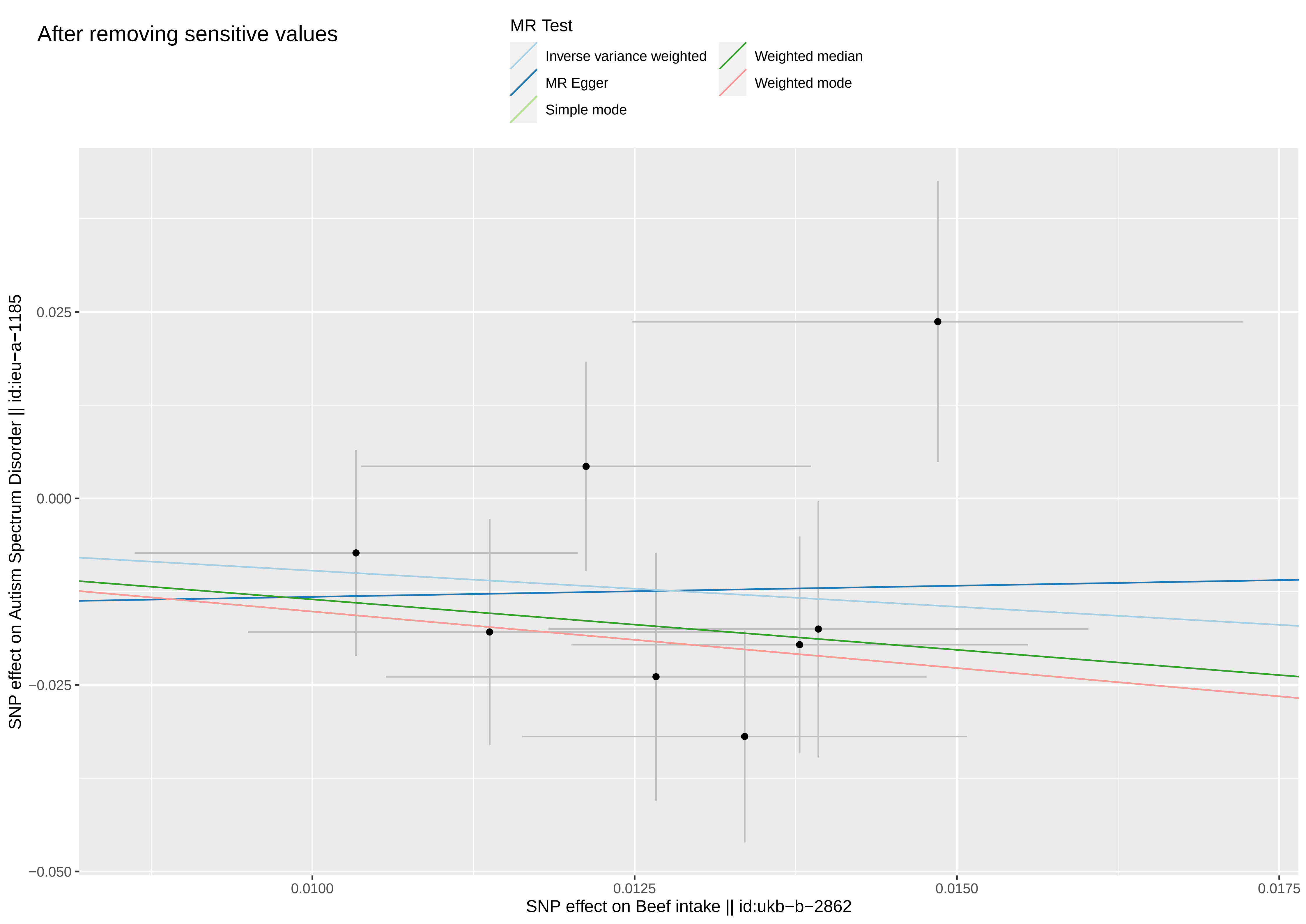

After removing sensitive values
MR Test
Inverse variance weighted
MR Egger
Simple mode
Weighted median
Weighted mode
0.025
0.000
−0.025
−0.050
0.0100
0.0125
0.0150
0.0175
SNP effect on Beef intake || id:ukb−b−2862
SNP effect on Autism Spectrum Disorder || id:ieu−a−1185

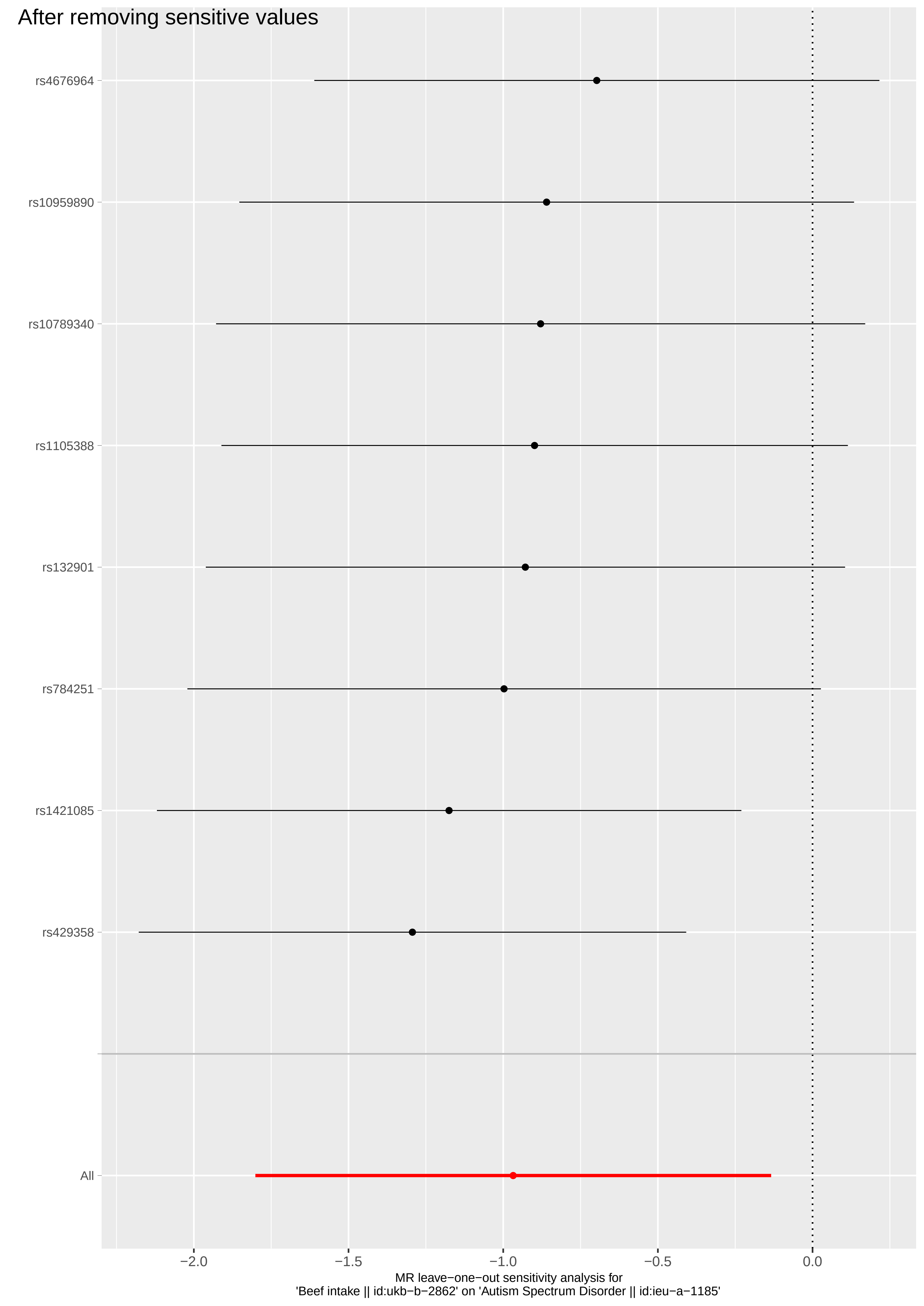
After removing sensitive values
rs4676964
rs10959890
rs10789340
rs1105388
rs132901
rs784251
rs1421085
rs429358
All
−2.0
−1.5
−1.0
−0.5
0.0
MR leave−one−out sensitivity analysis for
'Beef intake || id:ukb−b−2862' on 'Autism Spectrum Disorder || id:ieu−a−1185'

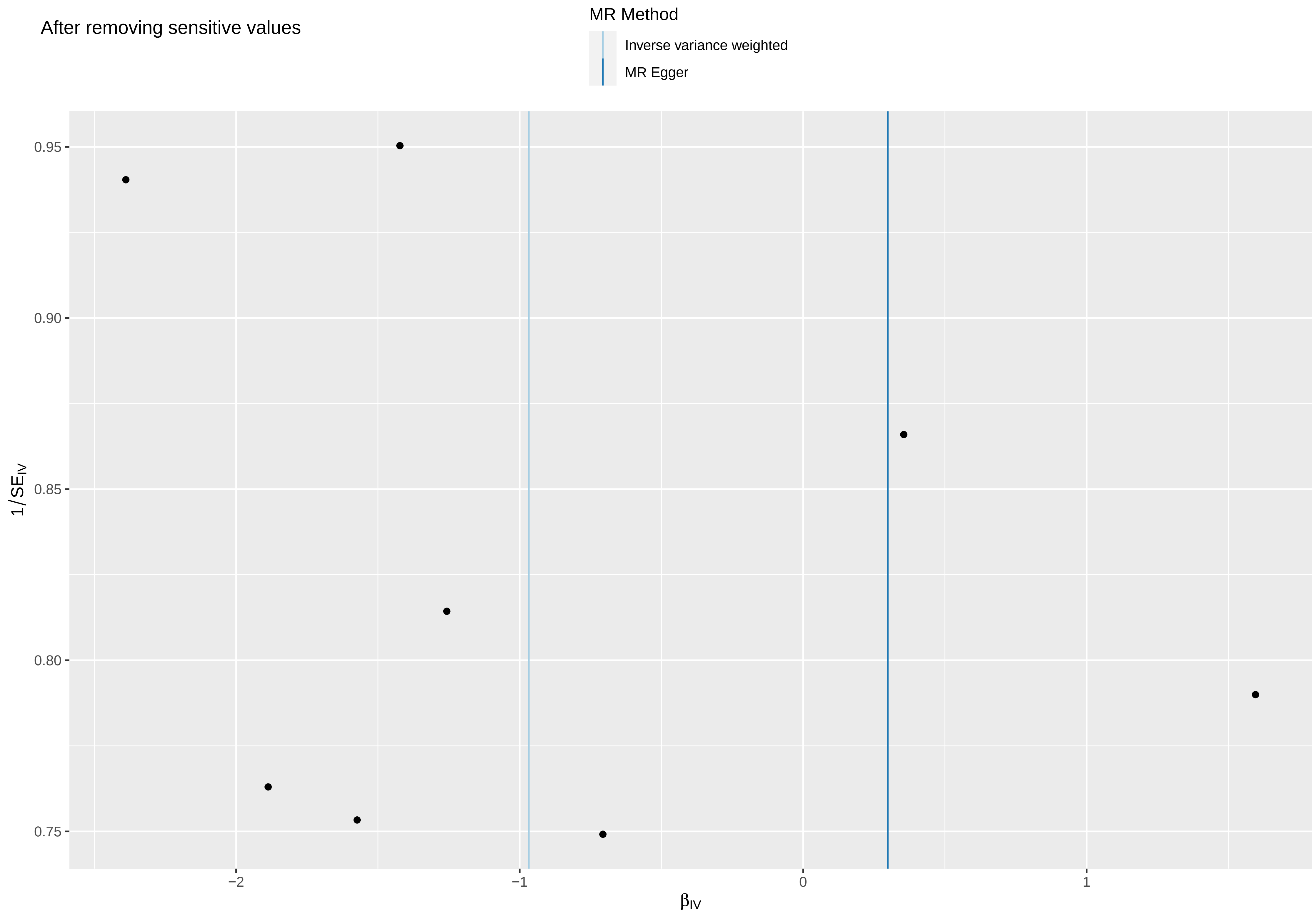

After removing sensitive values
MR Method
Inverse variance weighted
MR Egger
0.95
0.90
0.85
0.80
0.75
1/SE$_{IV}$
−2
−1
0
1
β$_{IV}$

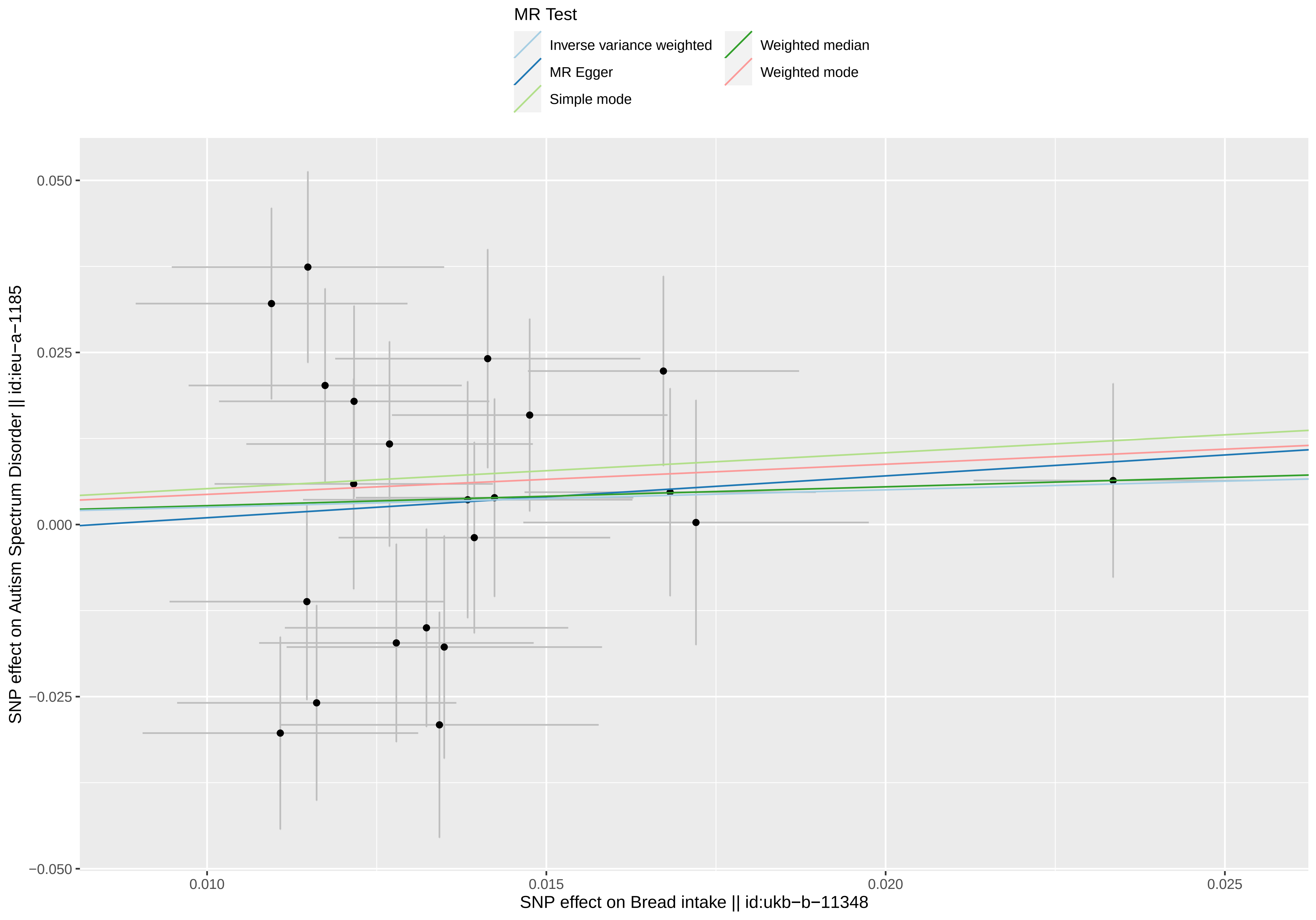
MR Test
Inverse variance weighted
MR Egger
Simple mode
Weighted median
Weighted mode
0.050
0.025
0.000
−0.025
−0.050
0.010
0.015
0.020
0.025
SNP effect on Autism Spectrum Disorder || id:ieu−a−1185
SNP effect on Bread intake || id:ukb−b−11348

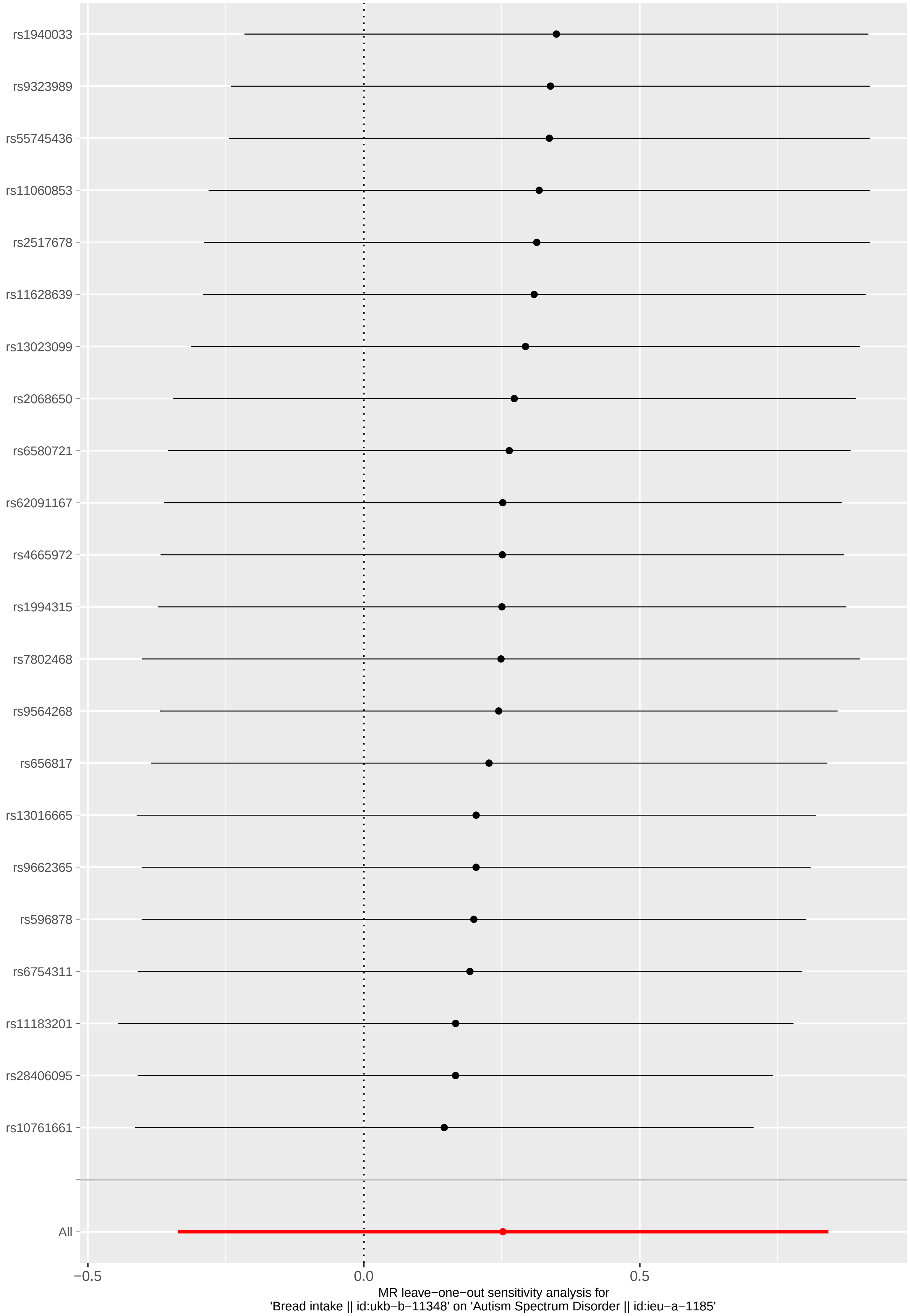

rs1940033
rs9323989
rs55745436
rs11060853
rs2517678
rs11628639
rs13023099
rs2068650
rs6580721
rs62091167
rs4665972
rs1994315
rs7802468
rs9564268
rs656817
rs13016665
rs9662365
rs596878
rs6754311
rs11183201
rs28406095
rs10761661
All
−0.5
0.0
0.5
MR leave−one−out sensitivity analysis for
'Bread intake || id:ukb−b−11348' on 'Autism Spectrum Disorder || id:ieu−a−1185'

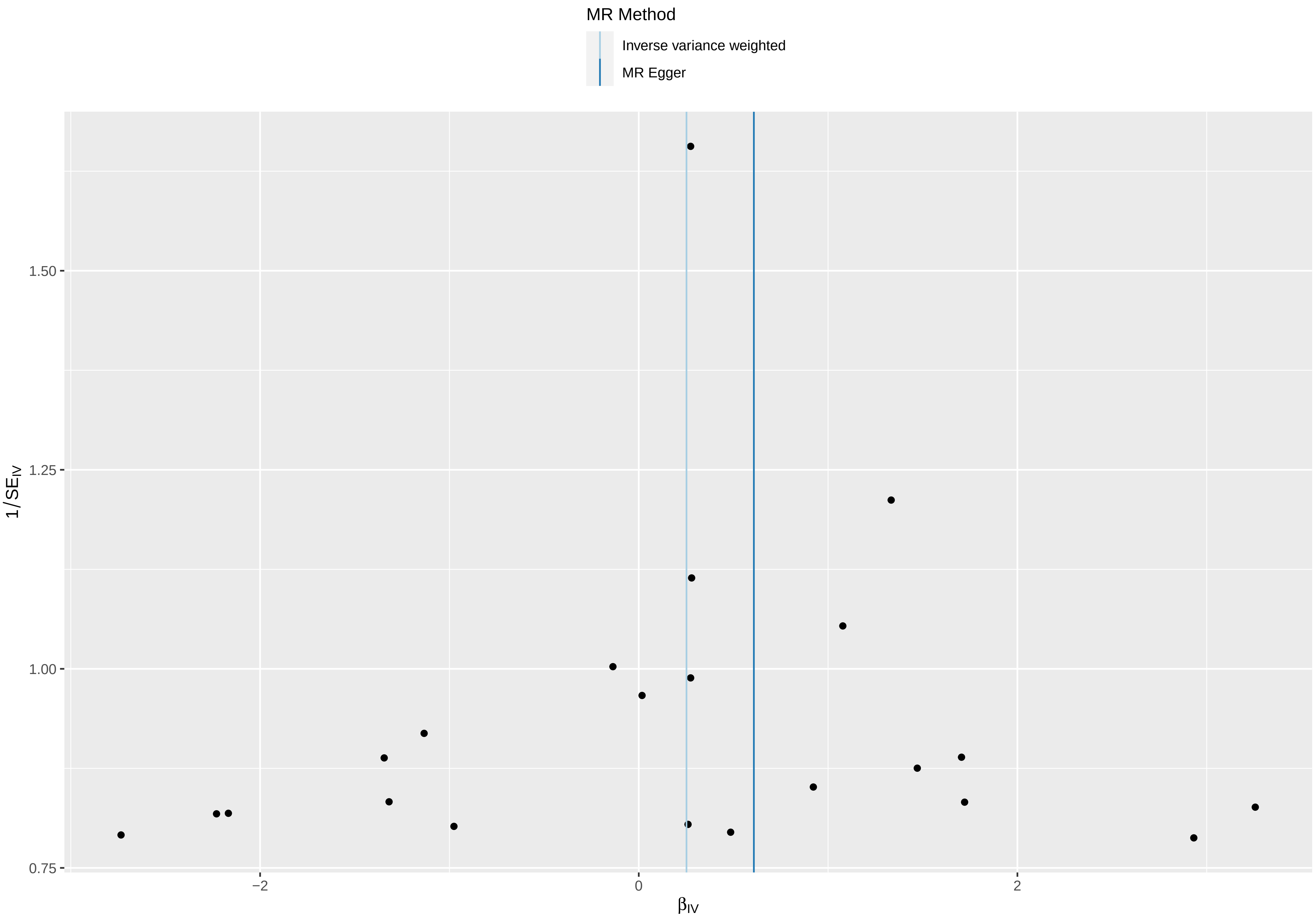
MR Method
Inverse variance weighted
MR Egger
1.50
1.25
1.00
0.75
1/SE$_{IV}$
−2
0
2
β$_{IV}$

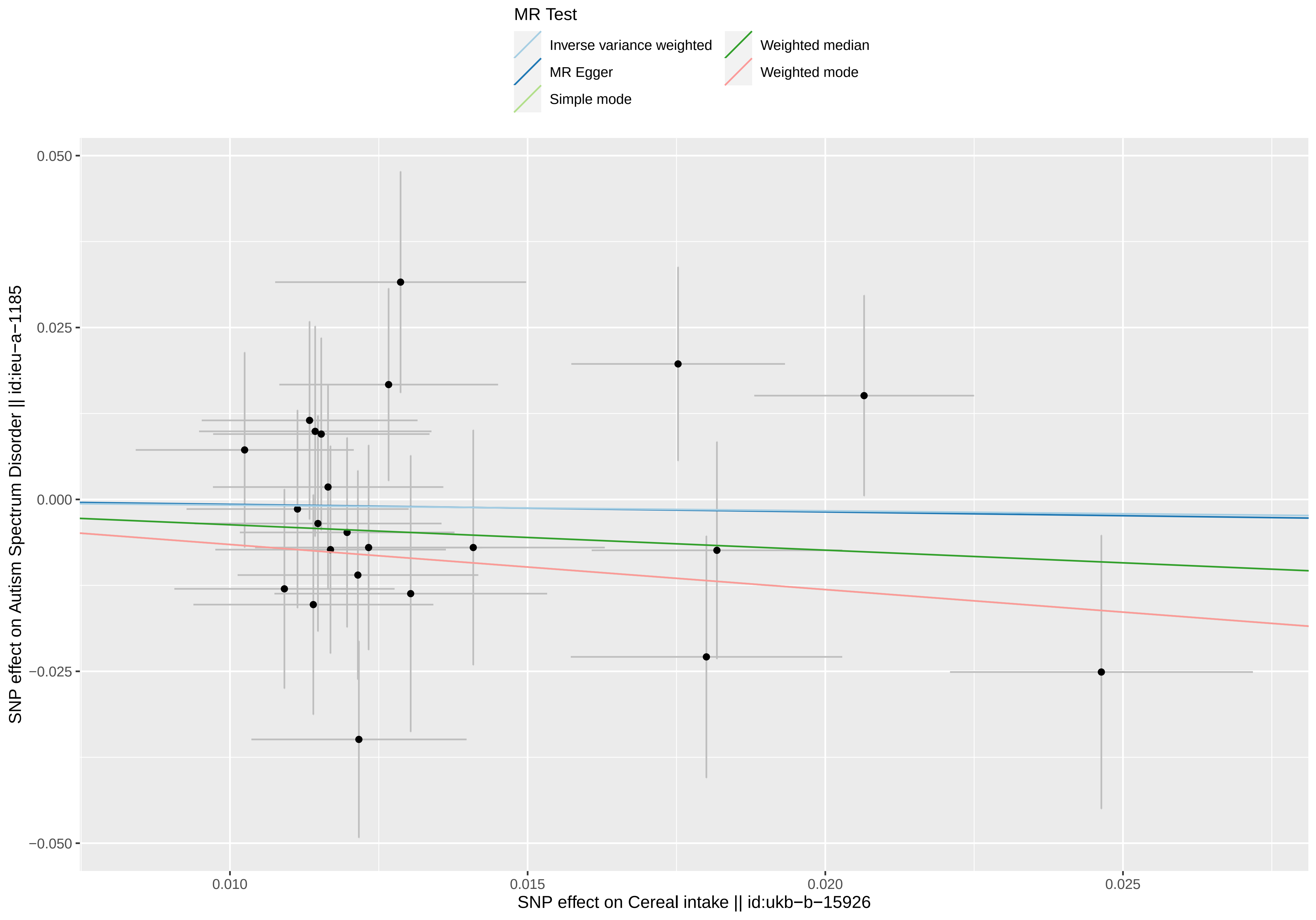

MR Test
Inverse variance weighted
MR Egger
Simple mode
Weighted median
Weighted mode
0.050
0.025
0.000
−0.025
−0.050
0.010
0.015
0.020
0.025
SNP effect on Autism Spectrum Disorder || id:ieu−a−1185
SNP effect on Cereal intake || id:ukb−b−15926

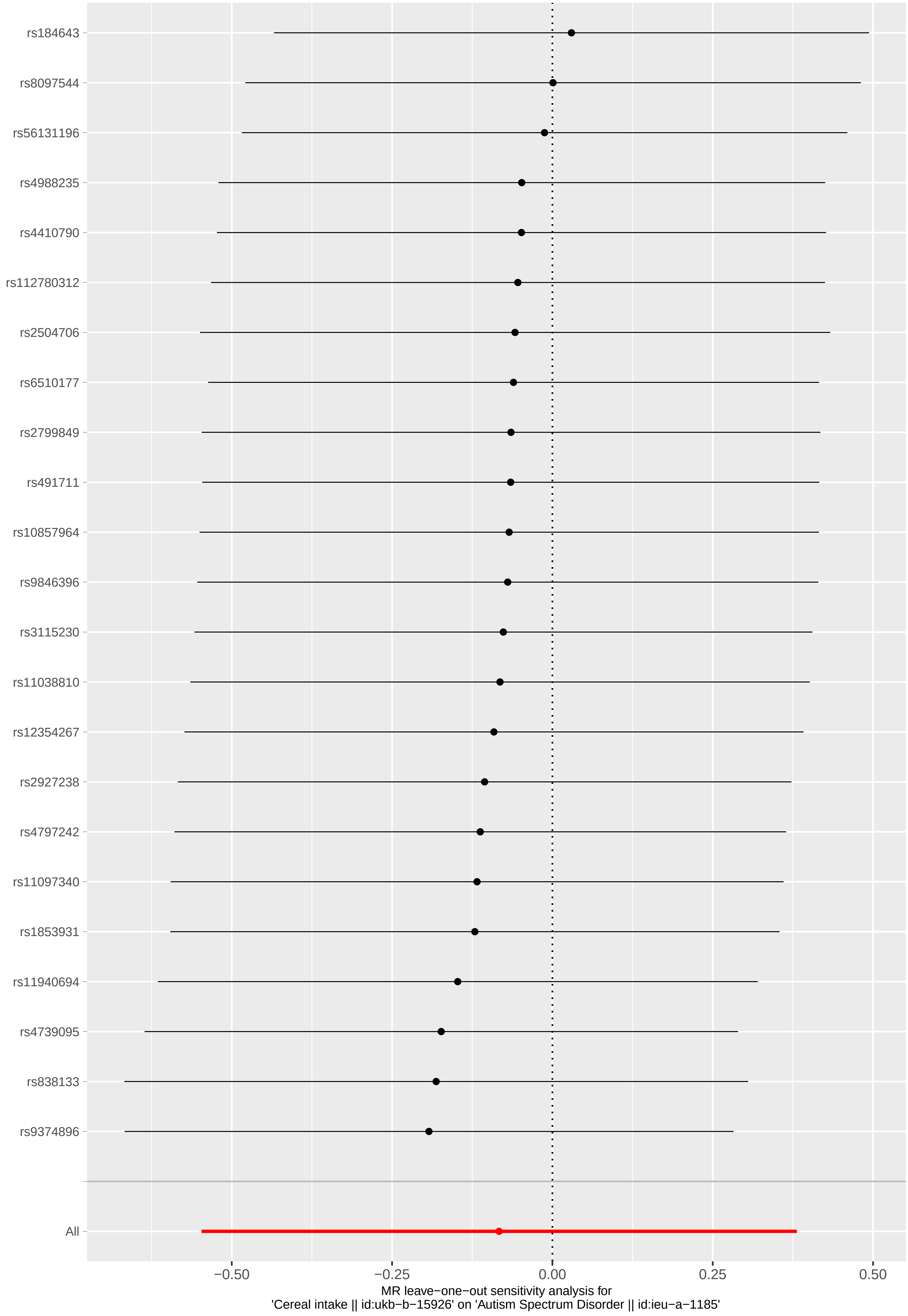
rs184643
rs8097544
rs56131196
rs4988235
rs4410790
rs112780312
rs2504706
rs6510177
rs2799849
rs491711
rs10857964
rs9846396
rs3115230
rs11038810
rs12354267
rs2927238
rs4797242
rs11097340
rs1853931
rs11940694
rs4739095
rs838133
rs9374896
All
−0.50
−0.25
0.00
0.25
0.50
MR leave−one−out sensitivity analysis for
'Cereal intake || id:ukb−b−15926' on 'Autism Spectrum Disorder || id:ieu−a−1185'

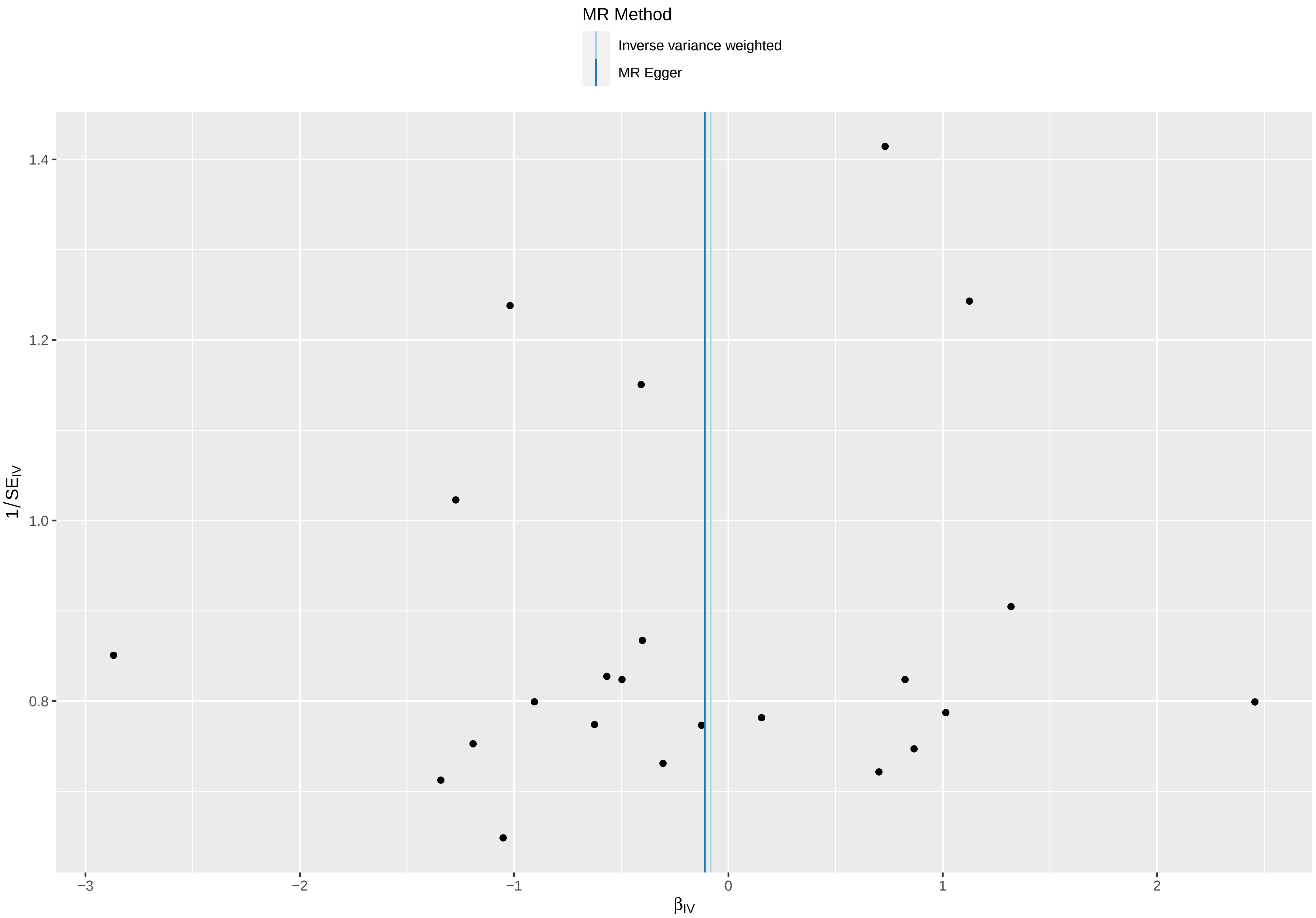

MR Method
Inverse variance weighted
MR Egger
1.4
1.2
1.0
0.8
1/SE_IV
−3
−2
−1
0
1
2
β_IV

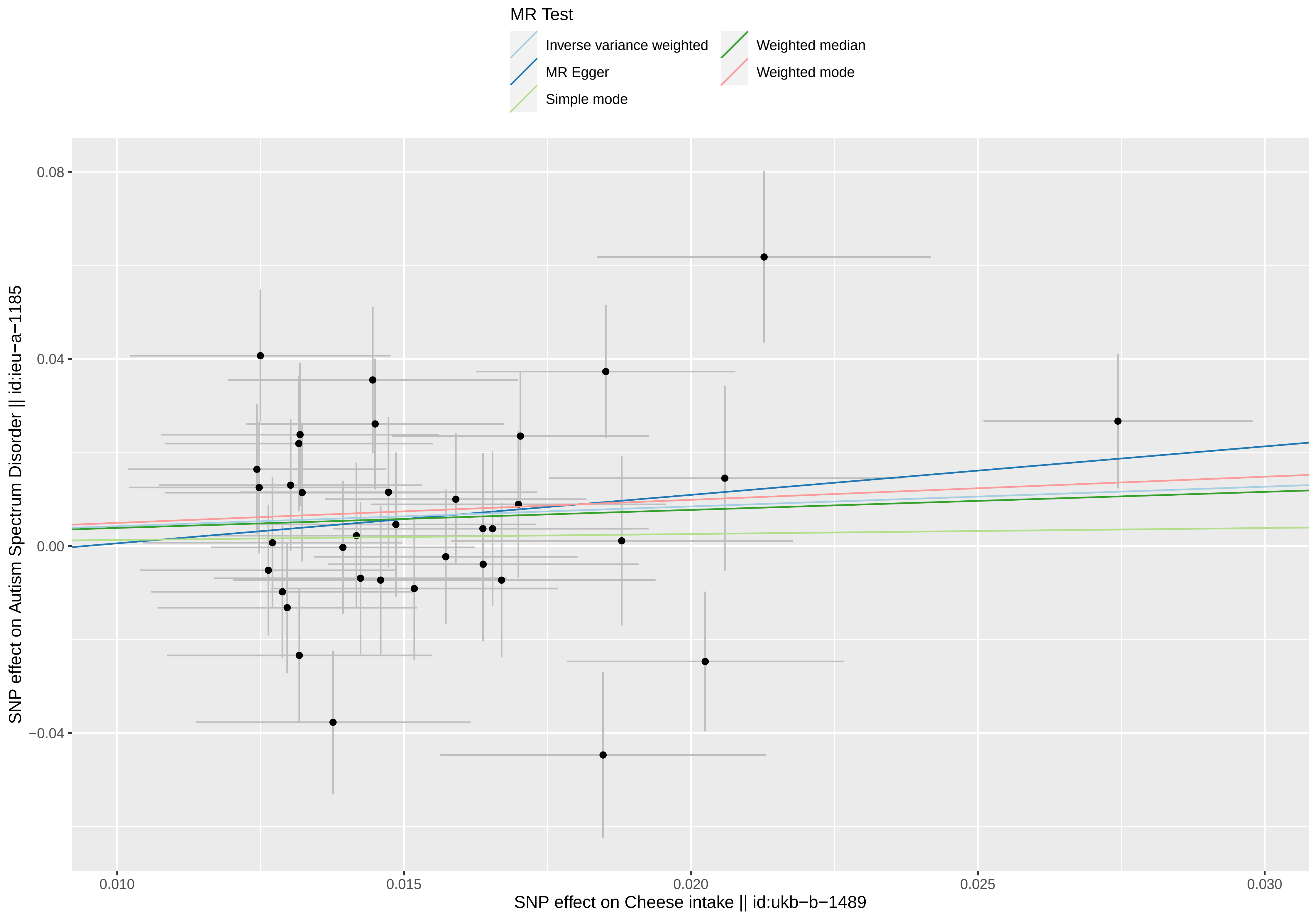
MR Test
Inverse variance weighted
MR Egger
Simple mode
Weighted median
Weighted mode
0.08
0.04
0.00
−0.04
0.010
0.015
0.020
0.025
0.030
SNP effect on Cheese intake || id:ukb−b−1489
SNP effect on Autism Spectrum Disorder || id:ieu−a−1185

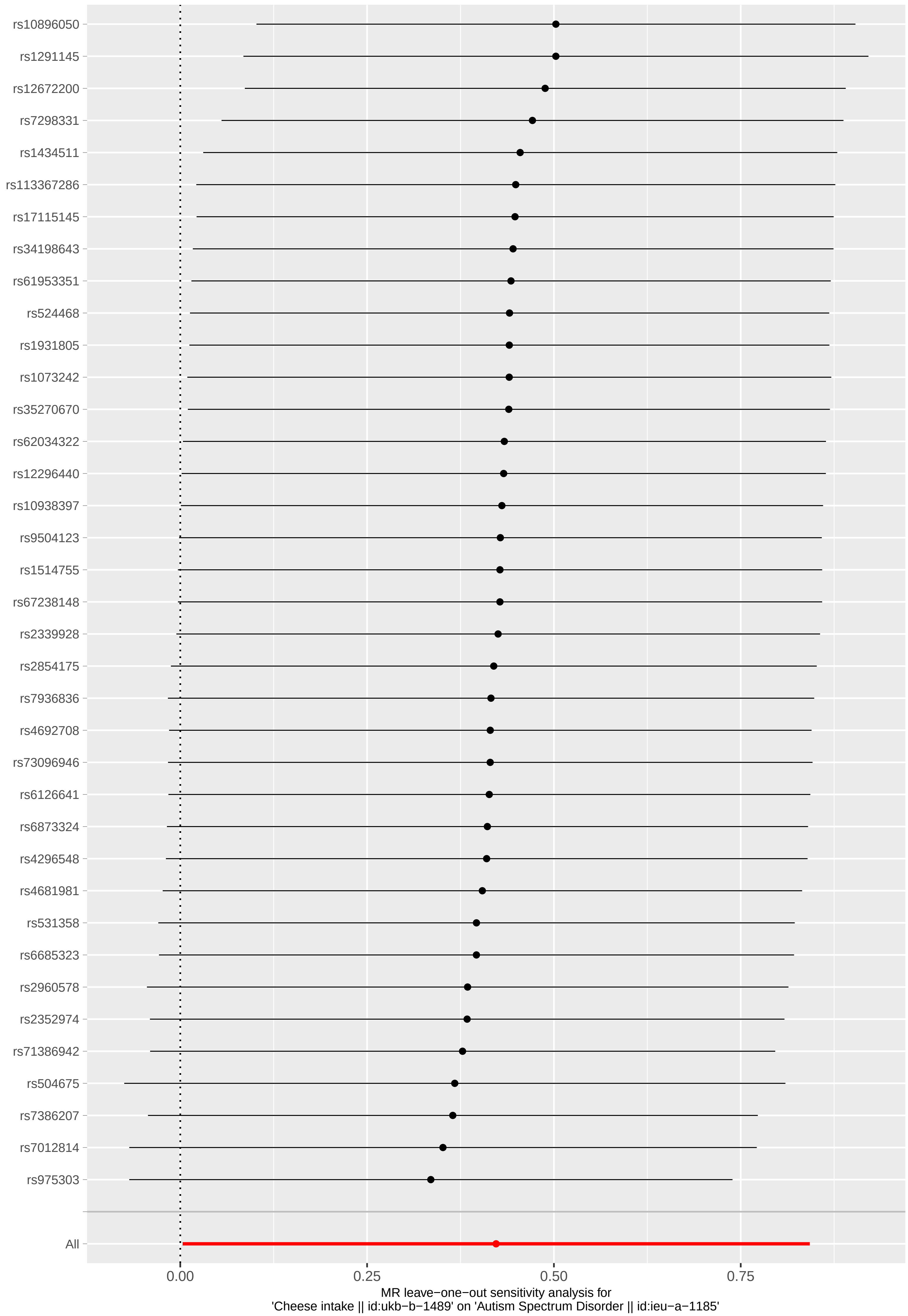
rs10896050
rs1291145
rs12672200
rs7298331
rs1434511
rs113367286
rs17115145
rs34198643
rs61953351
rs524468
rs1931805
rs1073242
rs35270670
rs62034322
rs12296440
rs10938397
rs9504123
rs1514755
rs67238148
rs2339928
rs2854175
rs7936836
rs4692708
rs73096946
rs6126641
rs6873324
rs4296548
rs4681981
rs531358
rs6685323
rs2960578
rs2352974
rs71386942
rs504675
rs7386207
rs7012814
rs975303
All
0.00
0.25
0.50
0.75
MR leave−one−out sensitivity analysis for
'Cheese intake || id:ukb−b−1489' on 'Autism Spectrum Disorder || id:ieu−a−1185'

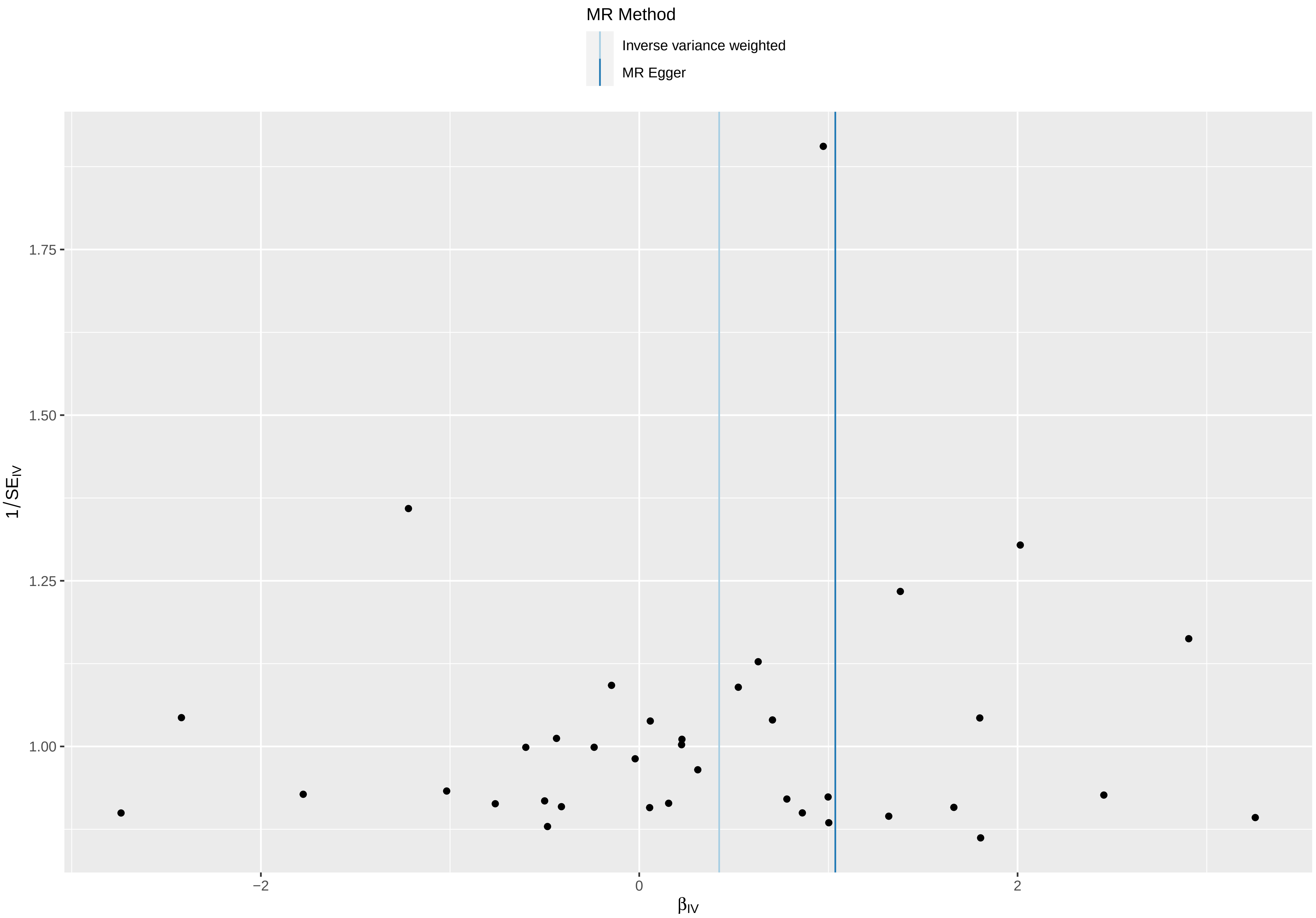

MR Method
Inverse variance weighted
MR Egger
1.75
1.50
1.25
1.00
$1/SE_{IV}$
−2
0
2
β_{IV}

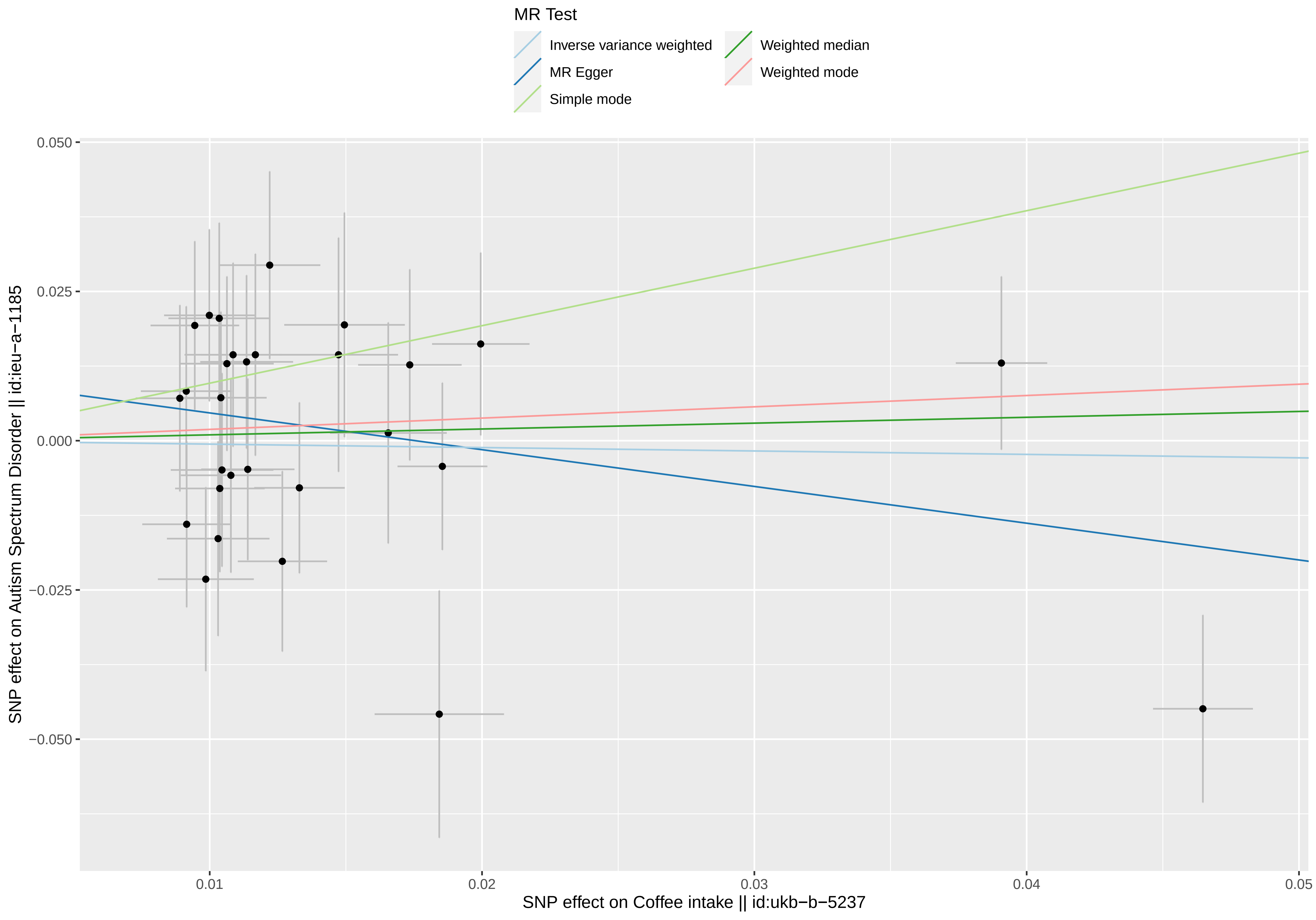
MR Test
Inverse variance weighted
MR Egger
Simple mode
Weighted median
Weighted mode
0.050
0.025
0.000
−0.025
−0.050
SNP effect on Autism Spectrum Disorder || id:ieu−a−1185
0.01
0.02
0.03
0.04
0.05
SNP effect on Coffee intake || id:ukb−b−5237

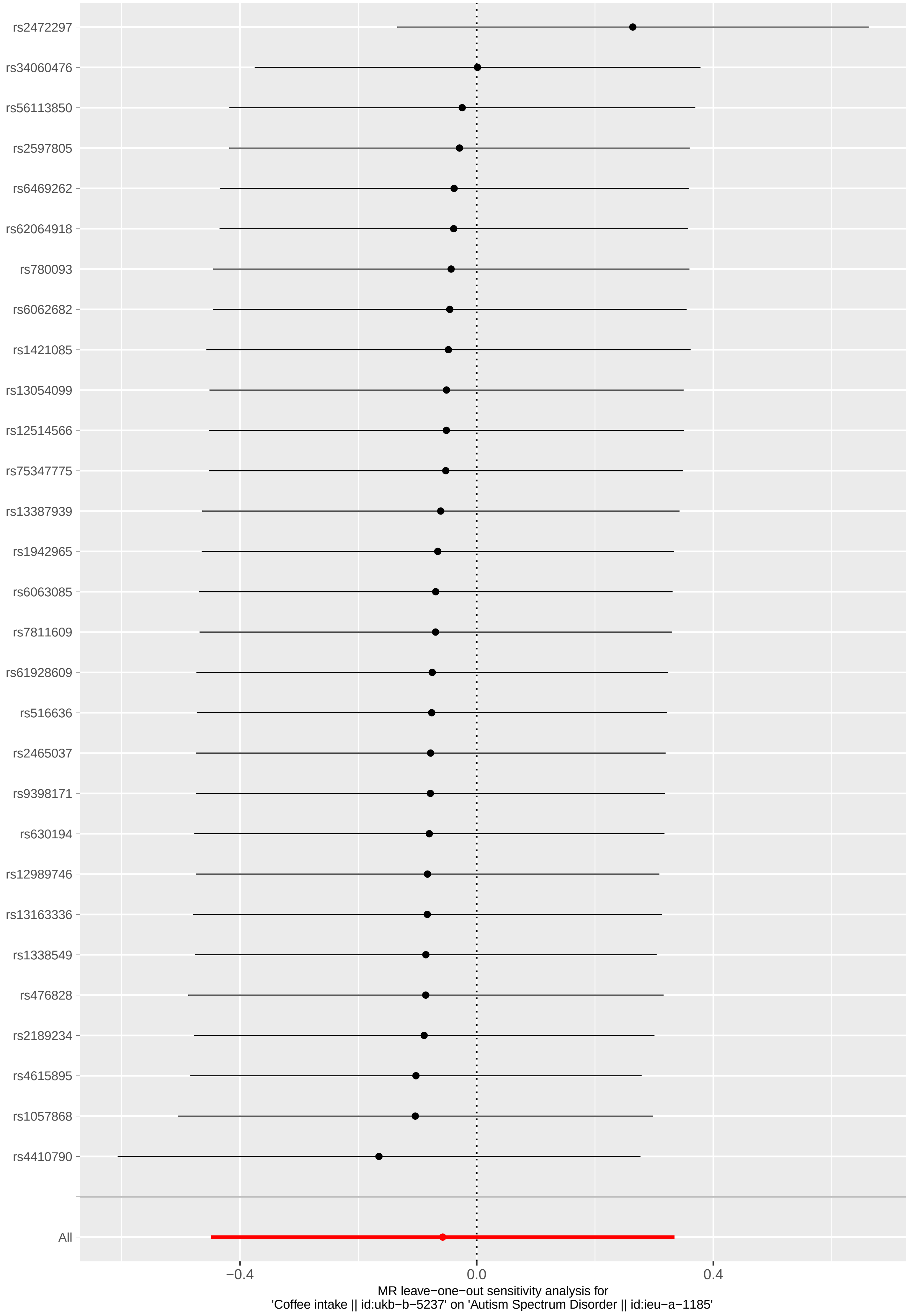

rs2472297
rs34060476
rs56113850
rs2597805
rs6469262
rs62064918
rs780093
rs6062682
rs1421085
rs13054099
rs12514566
rs75347775
rs13387939
rs1942965
rs6063085
rs7811609
rs61928609
rs516636
rs2465037
rs9398171
rs630194
rs12989746
rs13163336
rs1338549
rs476828
rs2189234
rs4615895
rs1057868
rs4410790
All
−0.4
0.0
0.4
MR leave−one−out sensitivity analysis for
'Coffee intake || id:ukb−b−5237' on 'Autism Spectrum Disorder || id:ieu−a−1185'

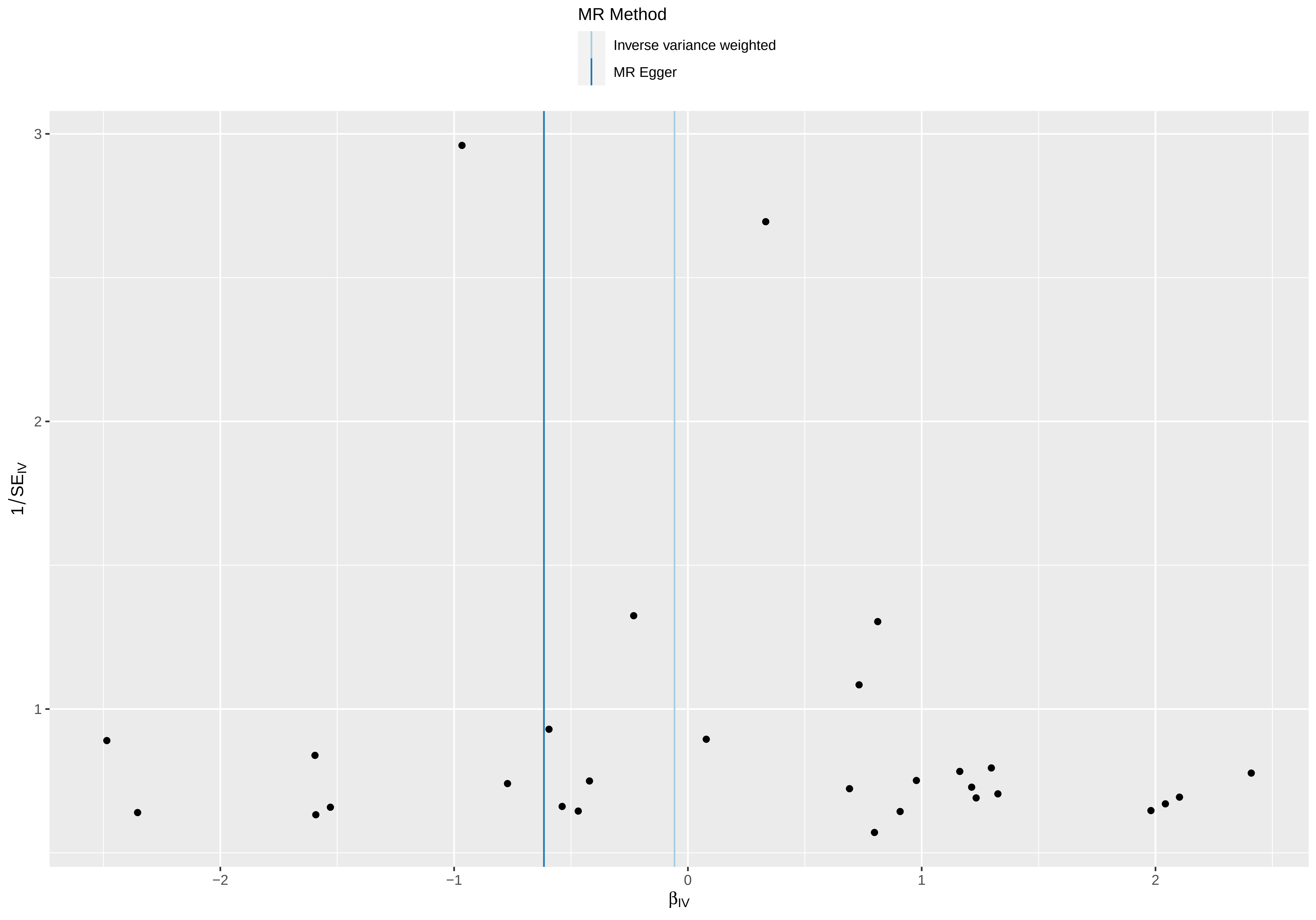

MR Method
Inverse variance weighted
MR Egger
3
2
1
1/SE_IV
−2
−1
0
1
2
β_IV

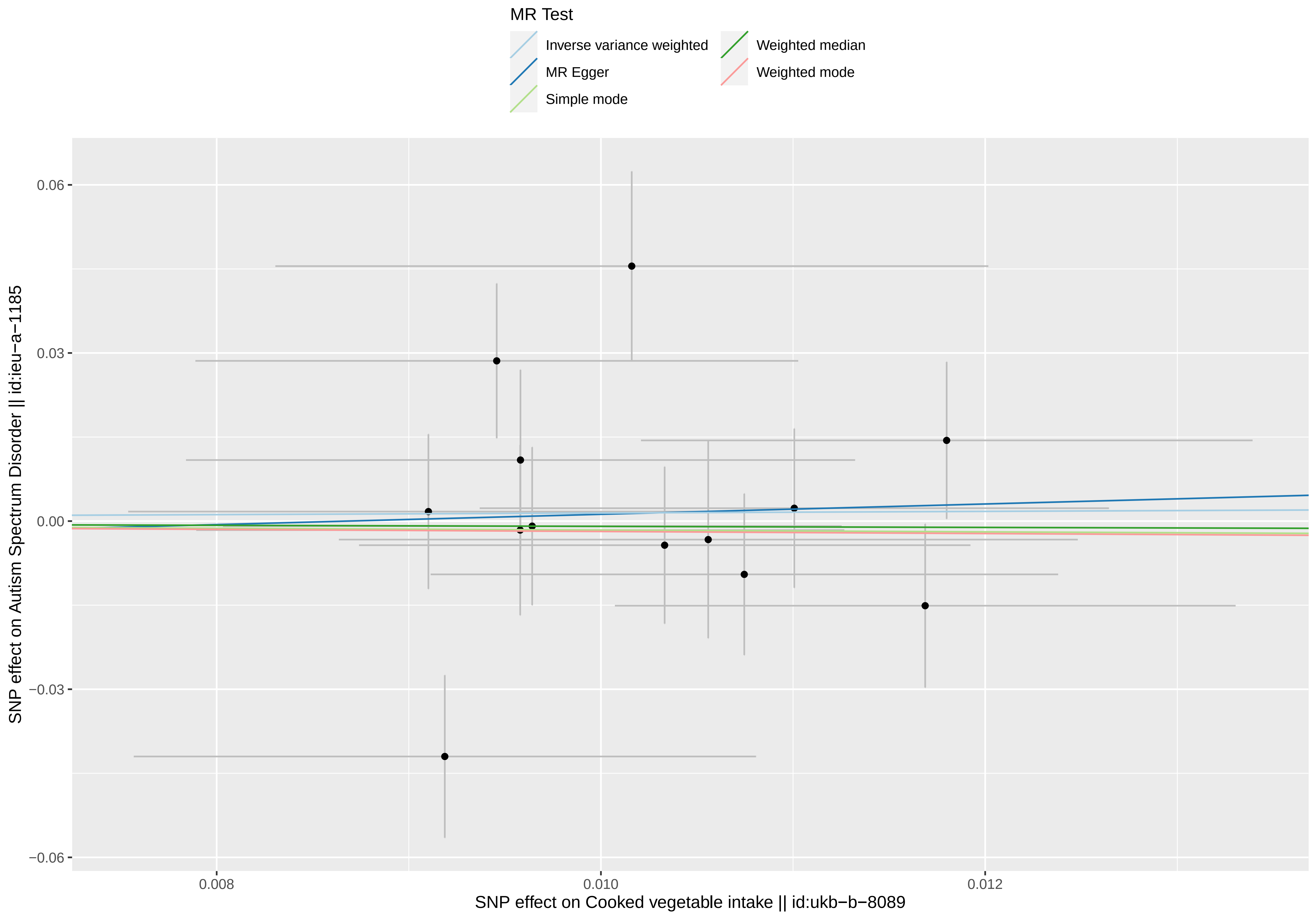

MR Test
Inverse variance weighted
MR Egger
Simple mode
Weighted median
Weighted mode
0.06
0.03
0.00
−0.03
−0.06
0.008
0.010
0.012
SNP effect on Cooked vegetable intake || id:ukb−b−8089
SNP effect on Autism Spectrum Disorder || id:ieu−a−1185

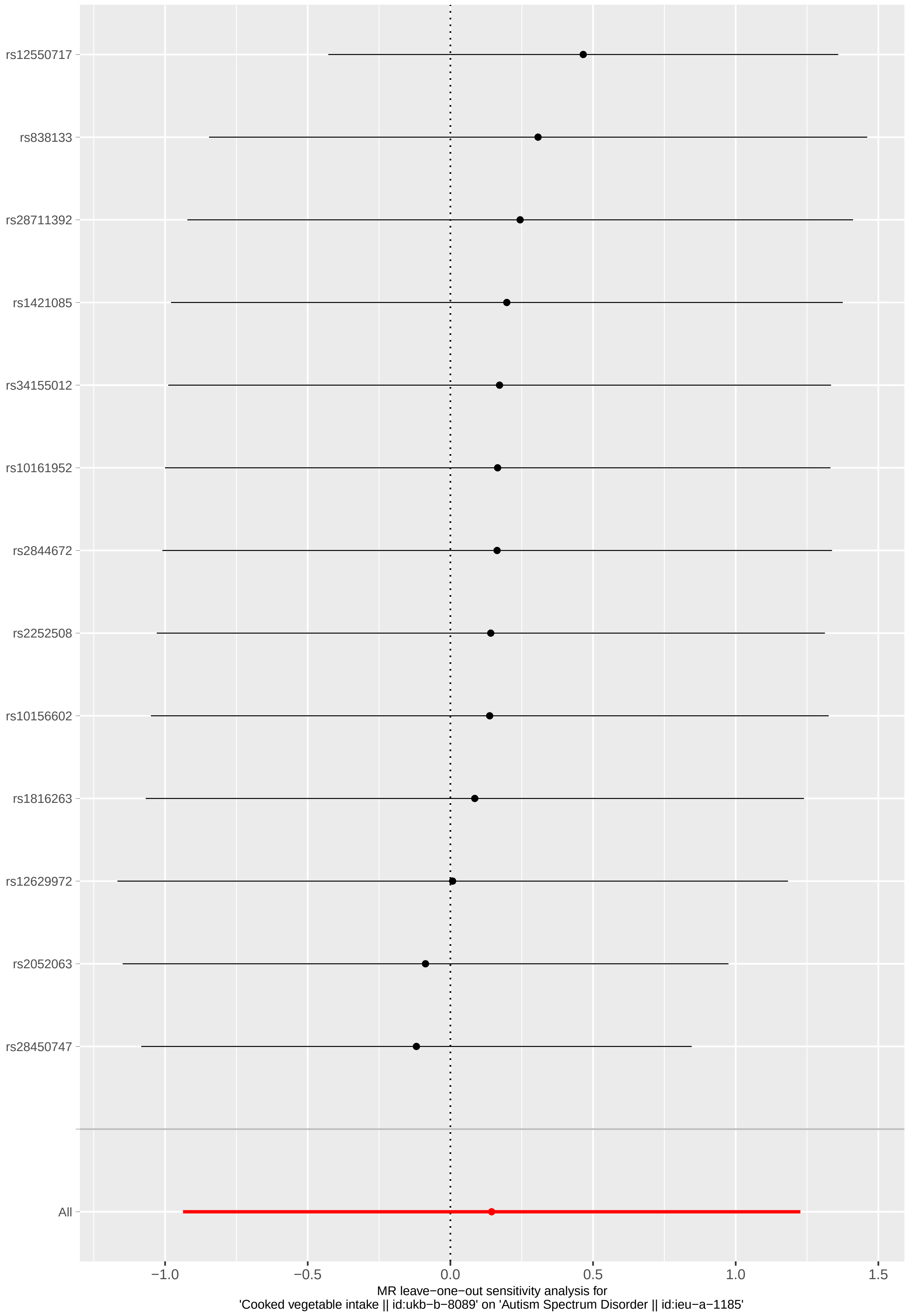
rs12550717
rs838133
rs28711392
rs1421085
rs34155012
rs10161952
rs2844672
rs2252508
rs10156602
rs1816263
rs12629972
rs2052063
rs28450747
All
−1.0
−0.5
0.0
0.5
1.0
1.5
MR leave−one−out sensitivity analysis for
'Cooked vegetable intake || id:ukb−b−8089' on 'Autism Spectrum Disorder || id:ieu−a−1185'

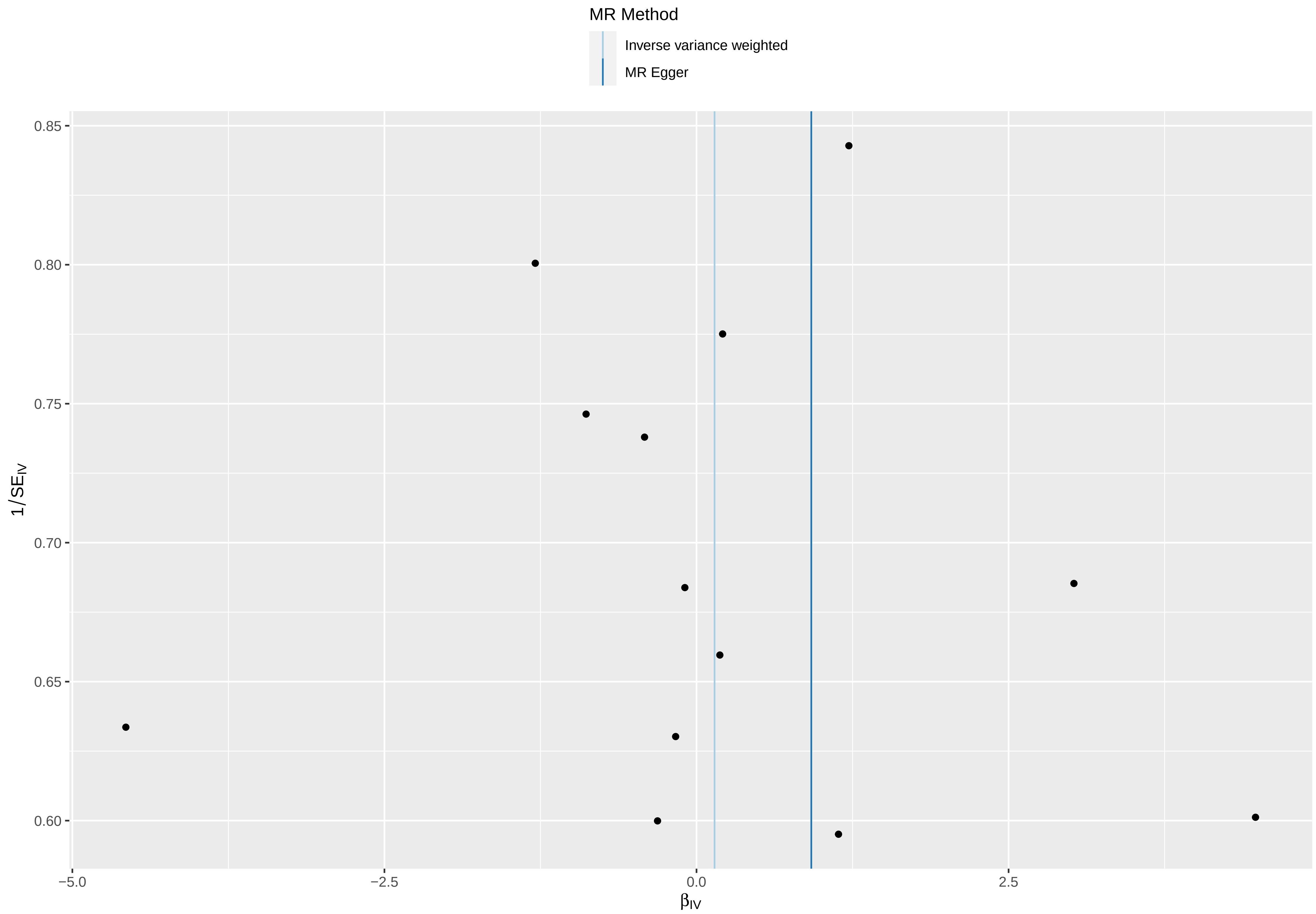
MR Method
Inverse variance weighted
MR Egger
0.85
0.80
0.75
0.70
0.65
0.60
1/SE_IV
−5.0
−2.5
0.0
2.5
β_IV

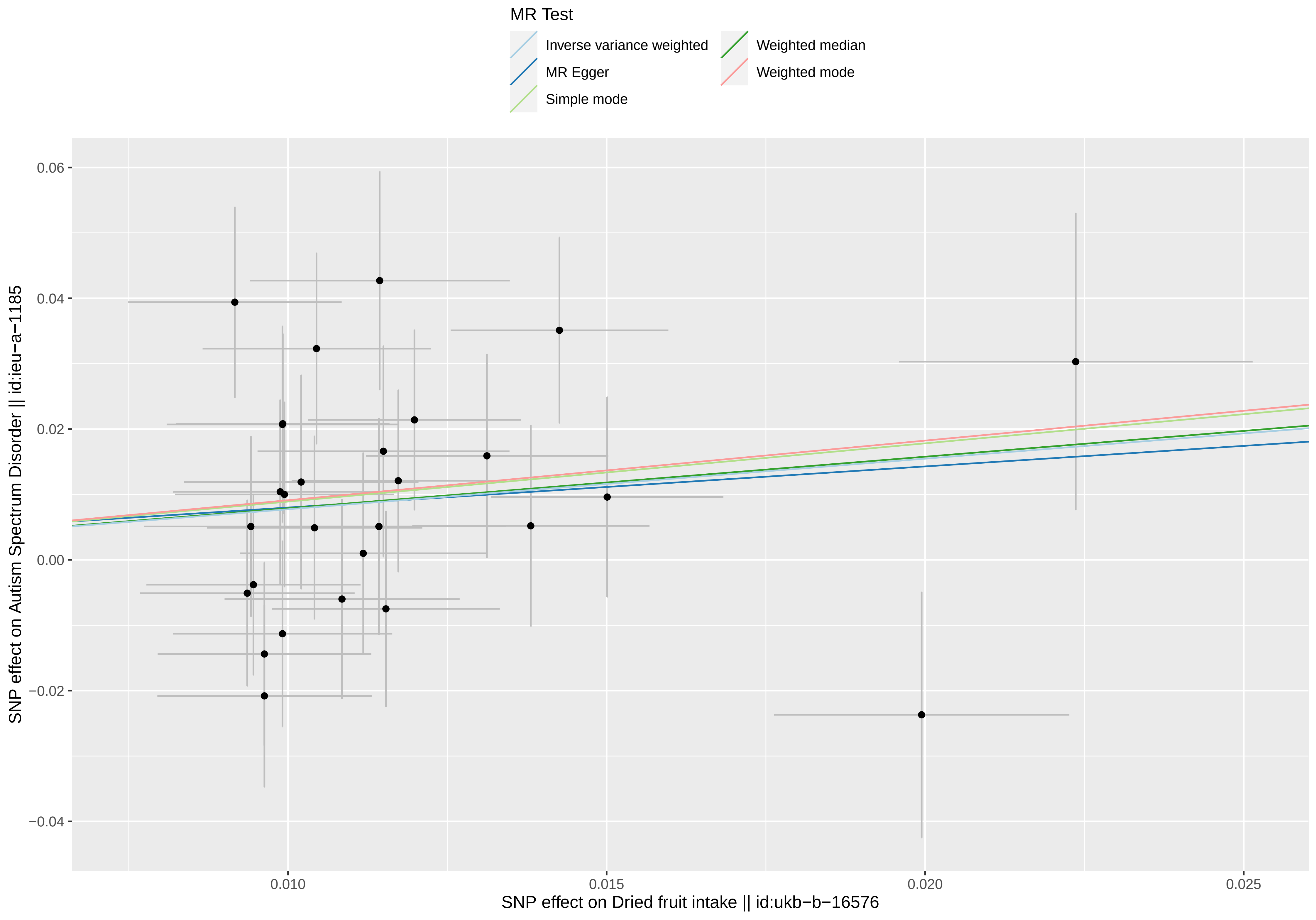
MR Test
Inverse variance weighted
MR Egger
Simple mode
Weighted median
Weighted mode
SNP effect on Autism Spectrum Disorder || id:ieu−a−1185
0.06
0.04
0.02
0.00
−0.02
−0.04
0.010
0.015
0.020
0.025
SNP effect on Dried fruit intake || id:ukb−b−16576

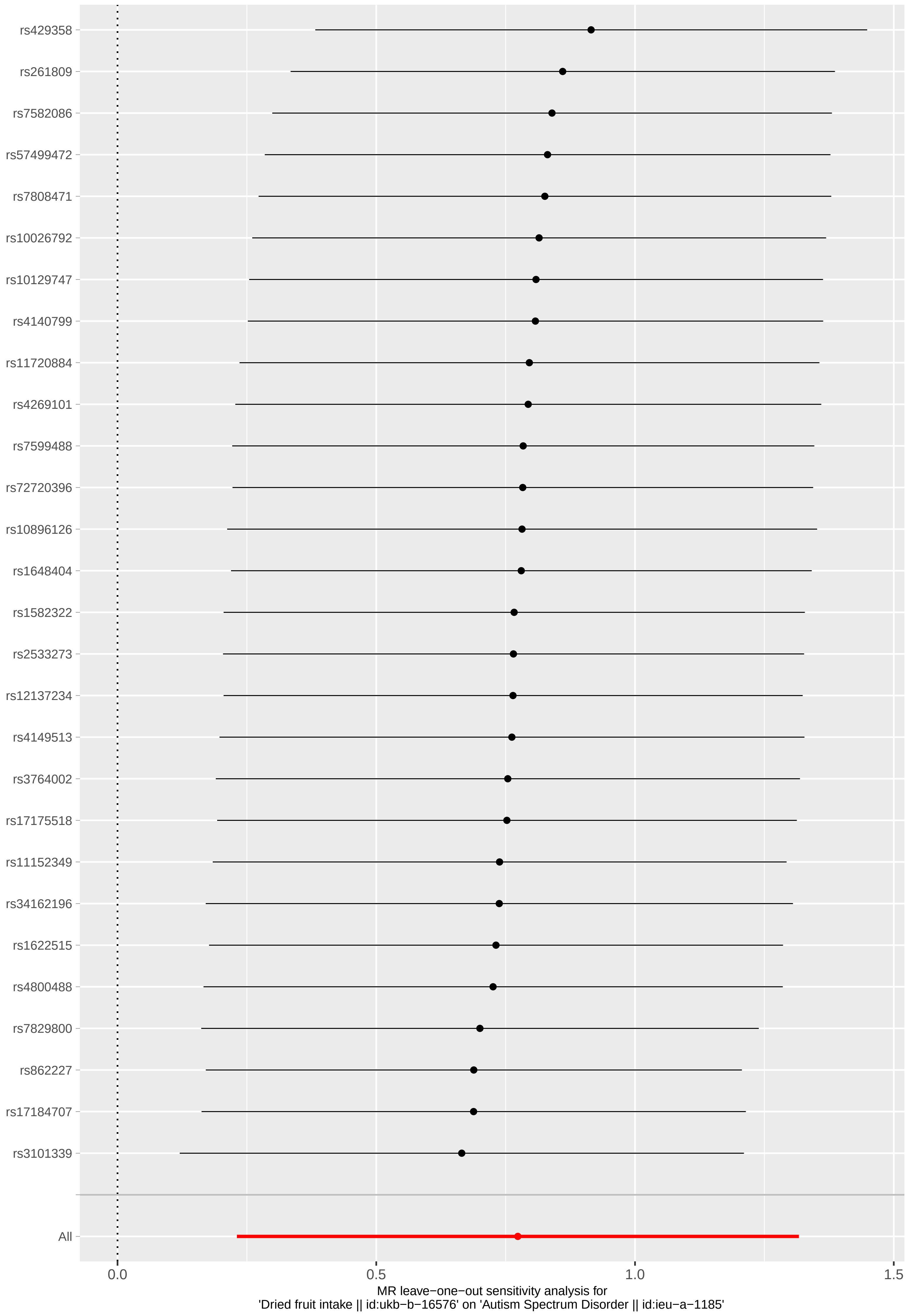
rs429358
rs261809
rs7582086
rs57499472
rs7808471
rs10026792
rs10129747
rs4140799
rs11720884
rs4269101
rs7599488
rs72720396
rs10896126
rs1648404
rs1582322
rs2533273
rs12137234
rs4149513
rs3764002
rs17175518
rs11152349
rs34162196
rs1622515
rs4800488
rs7829800
rs862227
rs17184707
rs3101339
All
0.0
0.5
1.0
1.5
MR leave−one−out sensitivity analysis for
'Dried fruit intake || id:ukb−b−16576' on 'Autism Spectrum Disorder || id:ieu−a−1185'

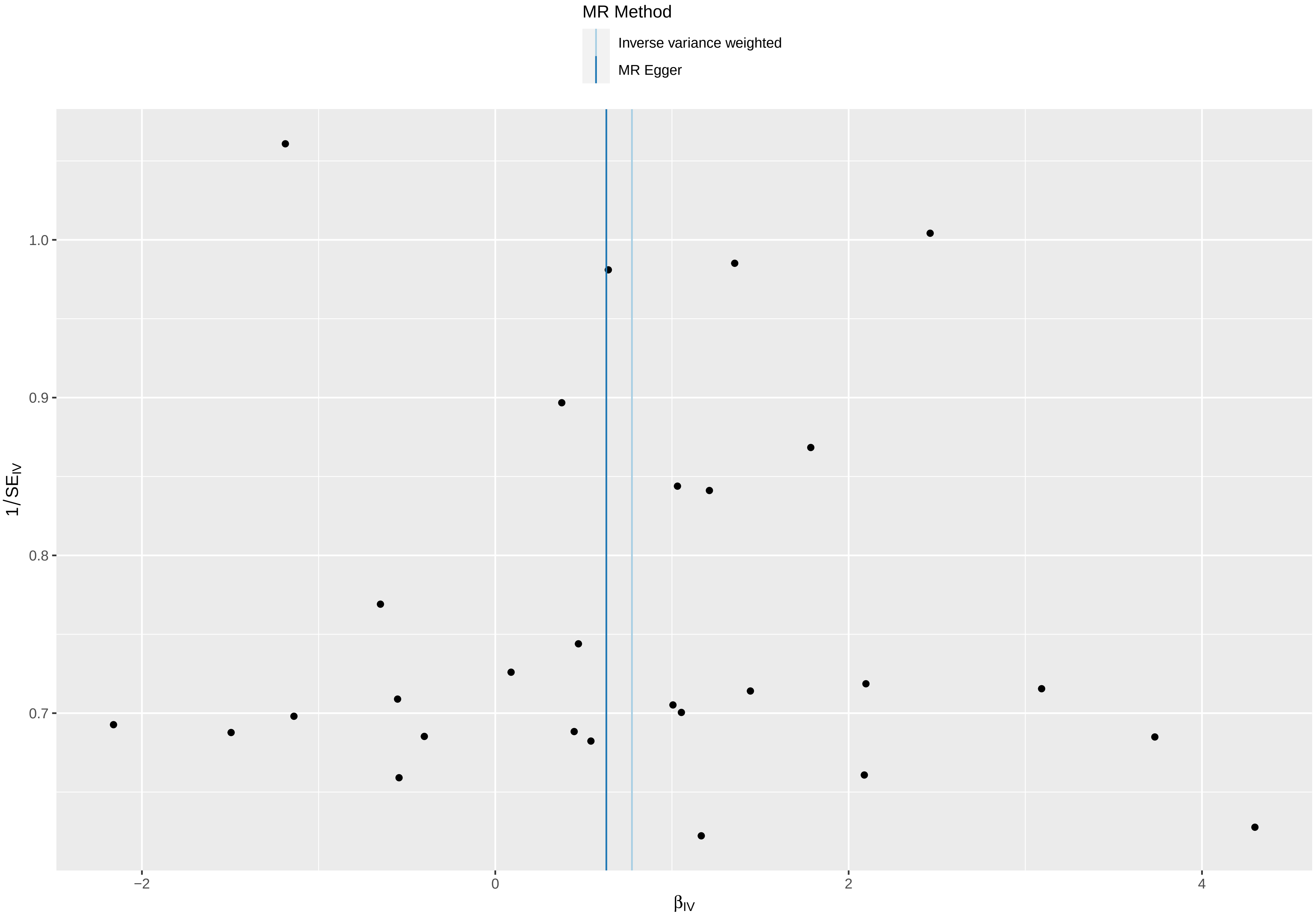
MR Method
Inverse variance weighted
MR Egger
1/SE$_{IV}$
1.0
0.9
0.8
0.7
−2
0
2
4
β$_{IV}$

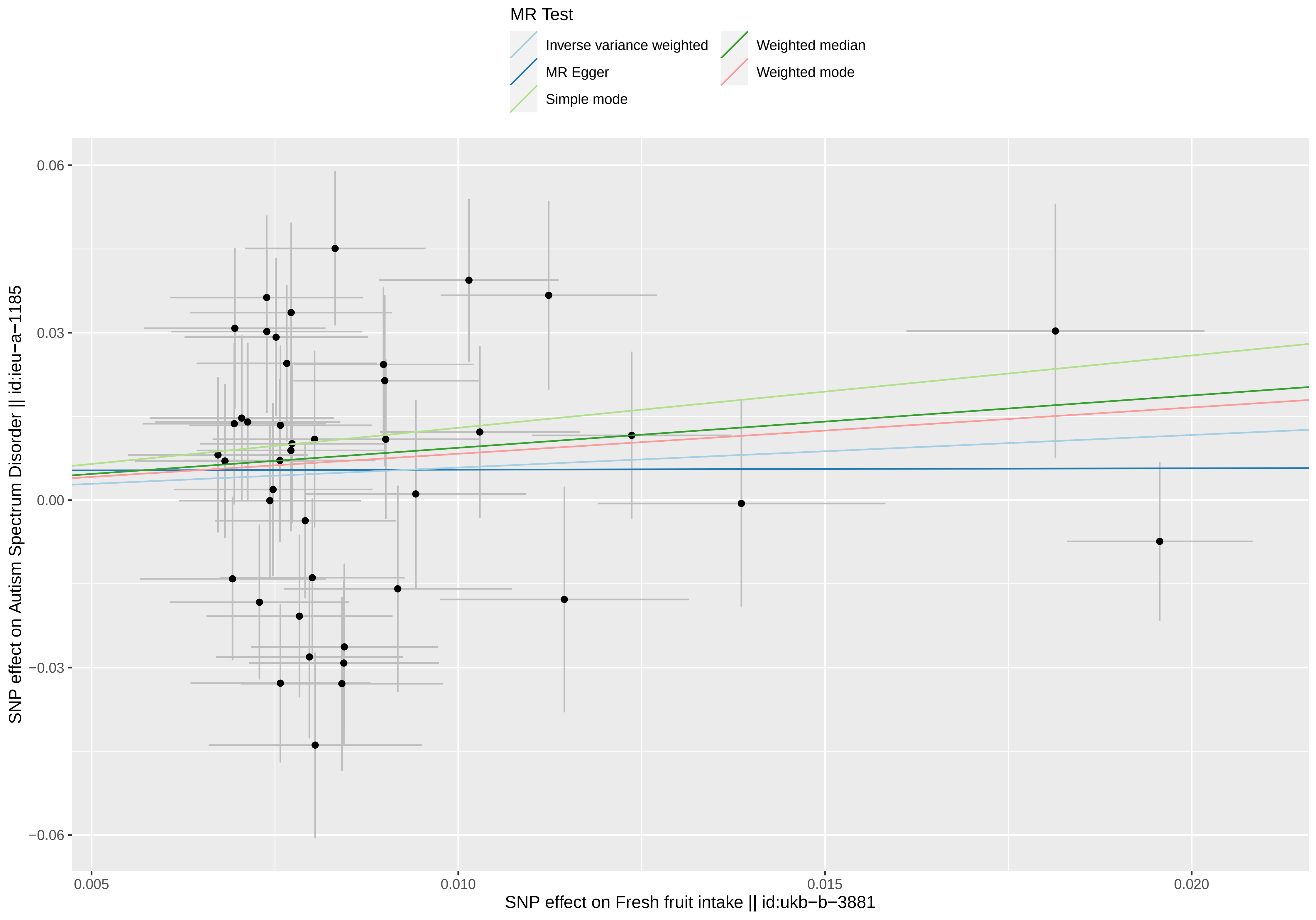

MR Test
Inverse variance weighted
MR Egger
Simple mode
Weighted median
Weighted mode
0.06
0.03
0.00
−0.03
−0.06
0.005
0.010
0.015
0.020
SNP effect on Fresh fruit intake || id:ukb−b−3881
SNP effect on Autism Spectrum Disorder || id:ieu−a−1185

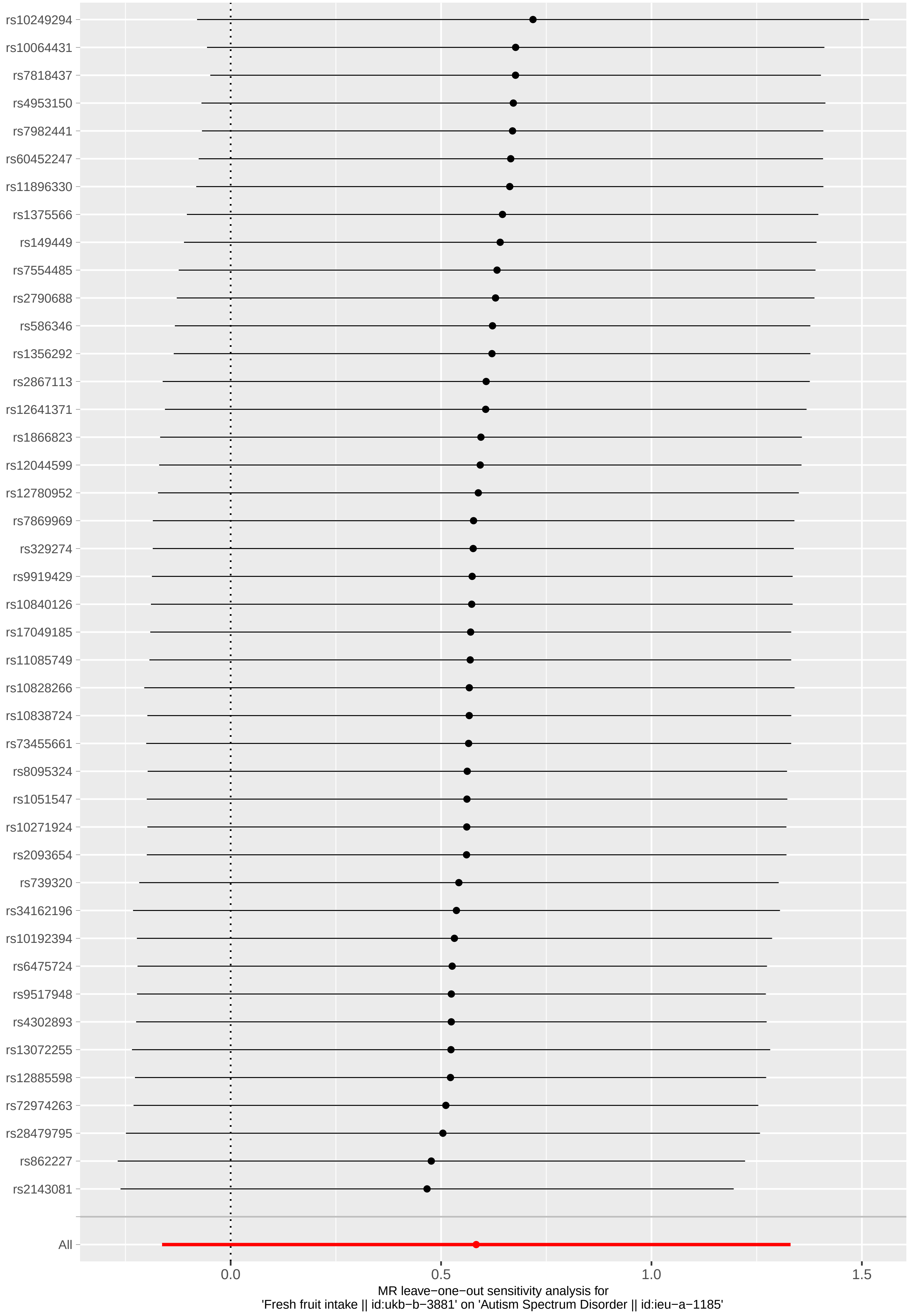

rs10249294
rs10064431
rs7818437
rs4953150
rs7982441
rs60452247
rs11896330
rs1375566
rs149449
rs7554485
rs2790688
rs586346
rs1356292
rs2867113
rs12641371
rs1866823
rs12044599
rs12780952
rs7869969
rs329274
rs9919429
rs10840126
rs17049185
rs11085749
rs10828266
rs10838724
rs73455661
rs8095324
rs1051547
rs10271924
rs2093654
rs739320
rs34162196
rs10192394
rs6475724
rs9517948
rs4302893
rs13072255
rs12885598
rs72974263
rs28479795
rs862227
rs2143081
All
0.0
0.5
1.0
1.5
MR leave−one−out sensitivity analysis for
'Fresh fruit intake || id:ukb−b−3881' on 'Autism Spectrum Disorder || id:ieu−a−1185'

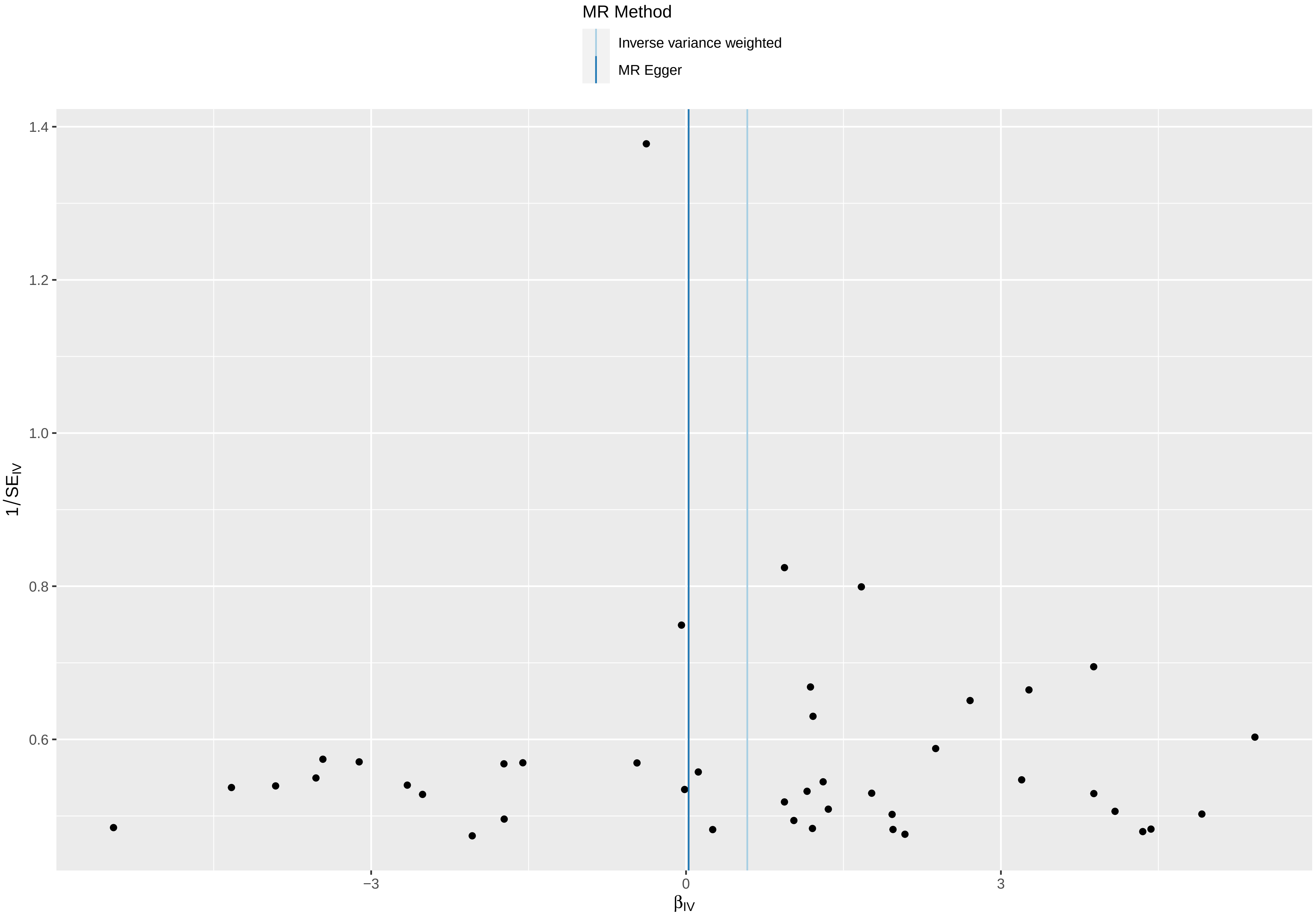
MR Method
Inverse variance weighted
MR Egger
1.4
1.2
1.0
0.8
0.6
$1/SE_{IV}$
−3
0
3
β_{IV}

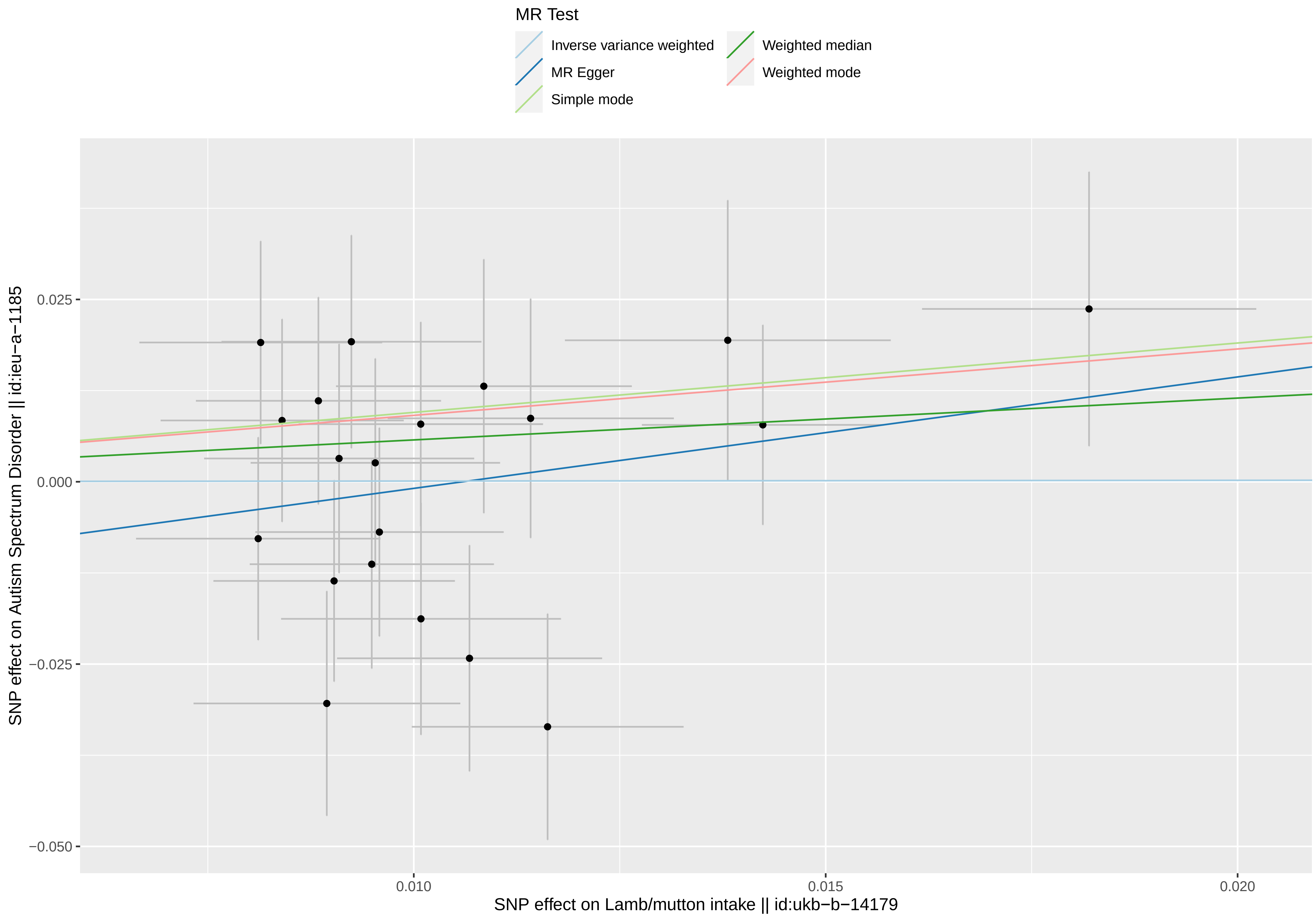
MR Test
Inverse variance weighted
MR Egger
Simple mode
Weighted median
Weighted mode
0.025
0.000
−0.025
−0.050
0.010
0.015
0.020
SNP effect on Autism Spectrum Disorder || id:ieu−a−1185
SNP effect on Lamb/mutton intake || id:ukb−b−14179

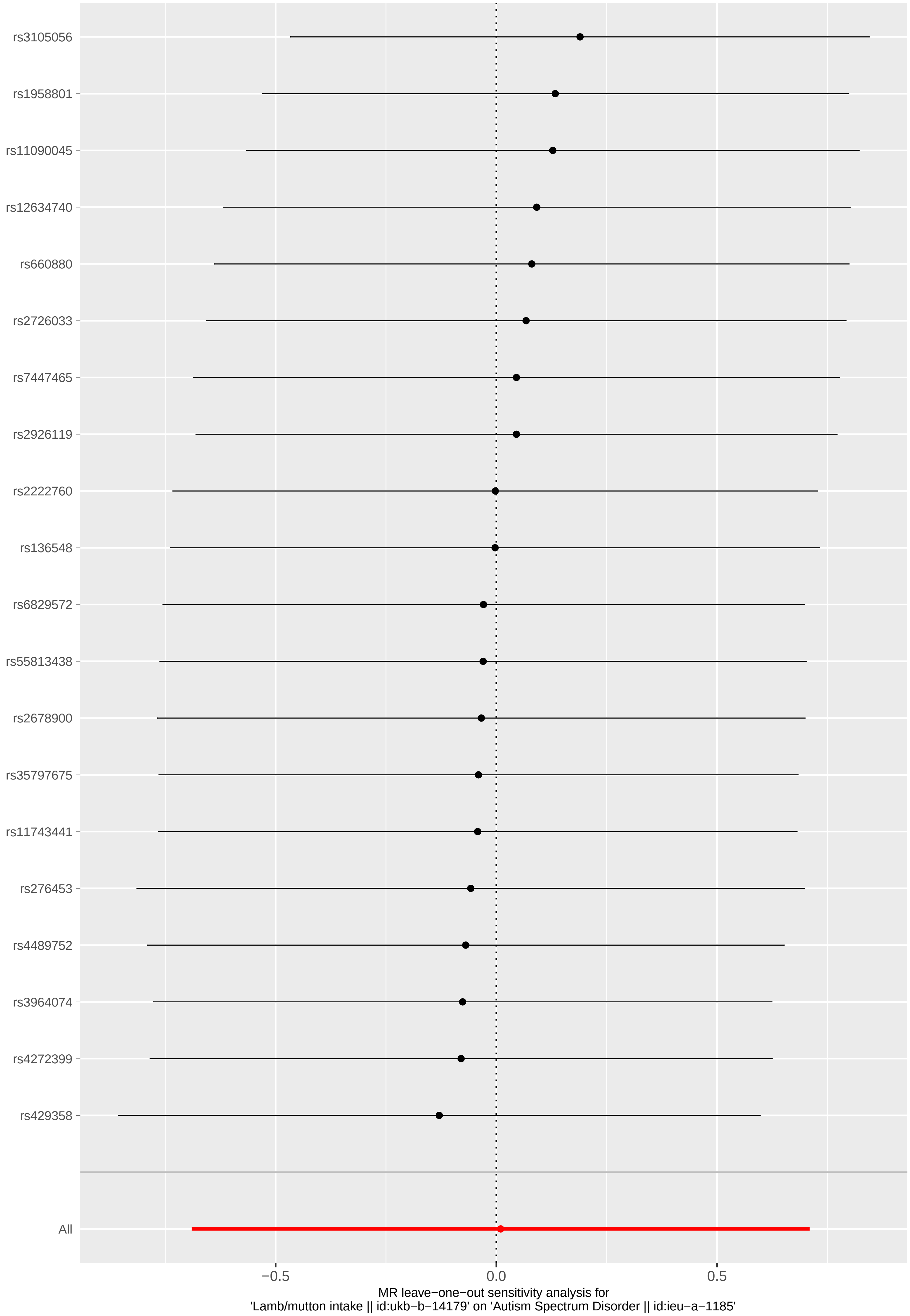

rs3105056
rs1958801
rs11090045
rs12634740
rs660880
rs2726033
rs7447465
rs2926119
rs2222760
rs136548
rs6829572
rs55813438
rs2678900
rs35797675
rs11743441
rs276453
rs4489752
rs3964074
rs4272399
rs429358
All
−0.5
0.0
0.5
MR leave−one−out sensitivity analysis for
'Lamb/mutton intake || id:ukb−b−14179' on 'Autism Spectrum Disorder || id:ieu−a−1185'

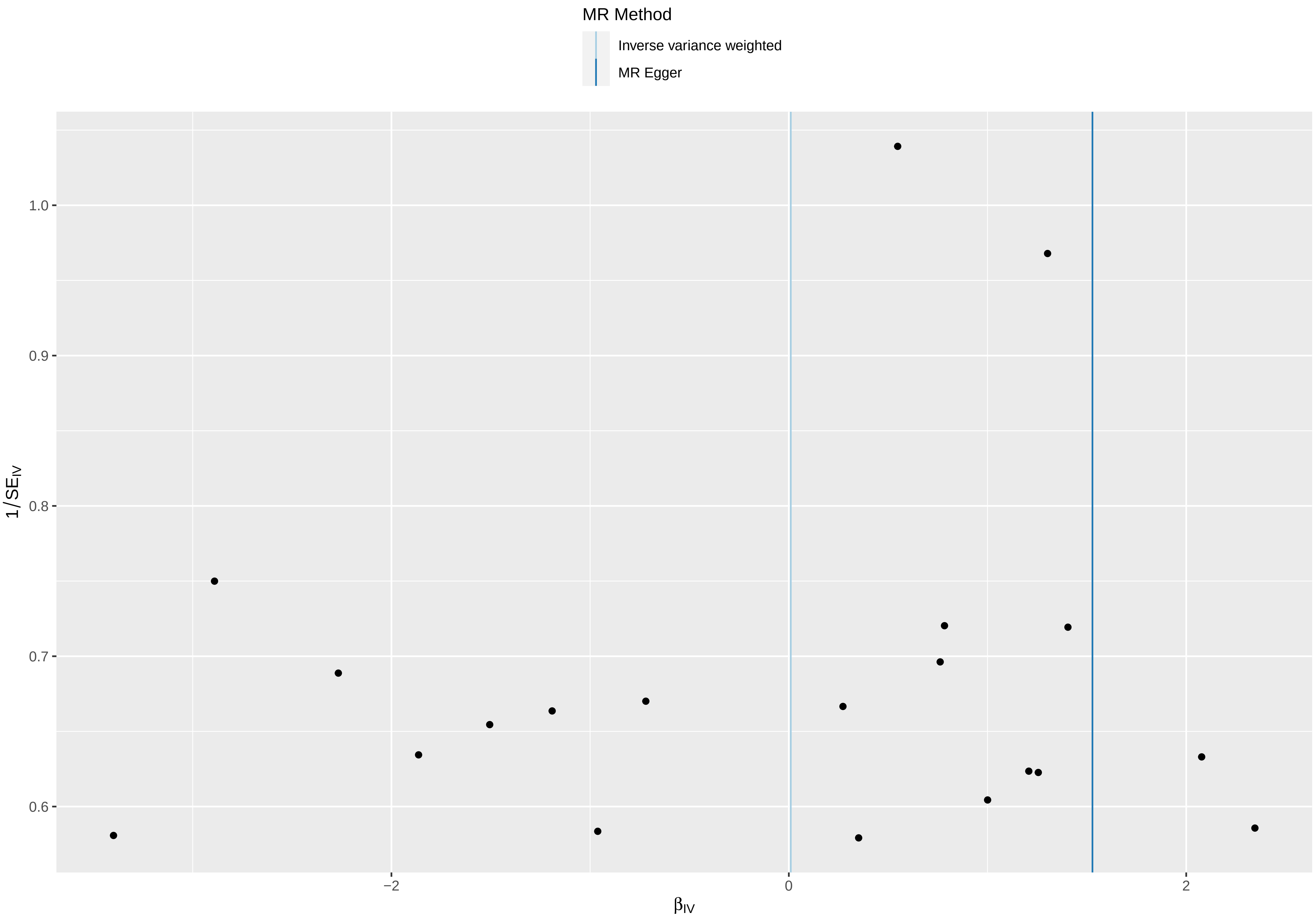
MR Method
Inverse variance weighted
MR Egger
1.0
0.9
0.8
0.7
0.6
−2
0
2
$1/SE_{IV}$
β_{IV}

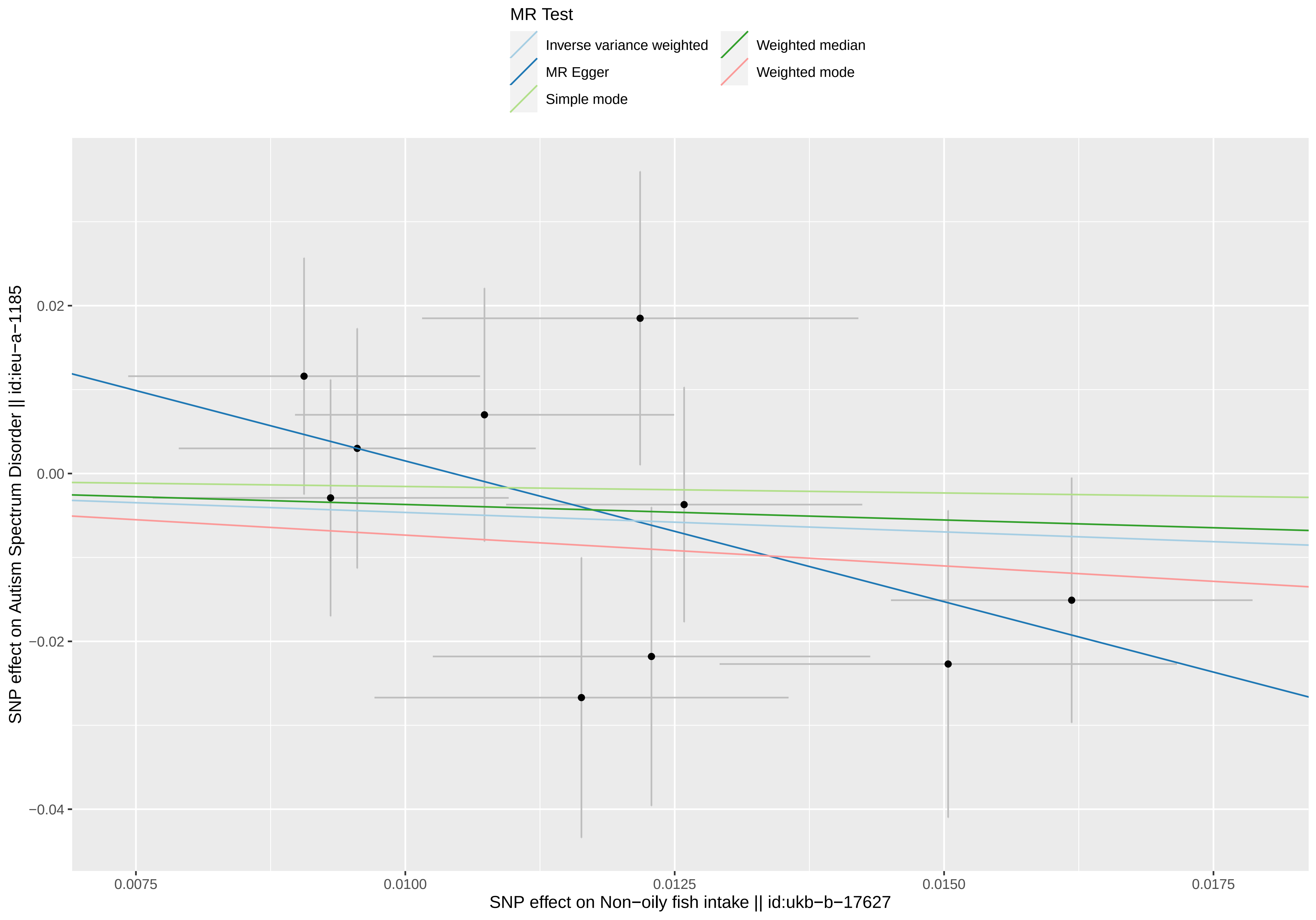

MR Test
Inverse variance weighted
MR Egger
Simple mode
Weighted median
Weighted mode
0.02
0.00
−0.02
−0.04
SNP effect on Autism Spectrum Disorder || id:ieu−a−1185
0.0075
0.0100
0.0125
0.0150
0.0175
SNP effect on Non−oily fish intake || id:ukb−b−17627

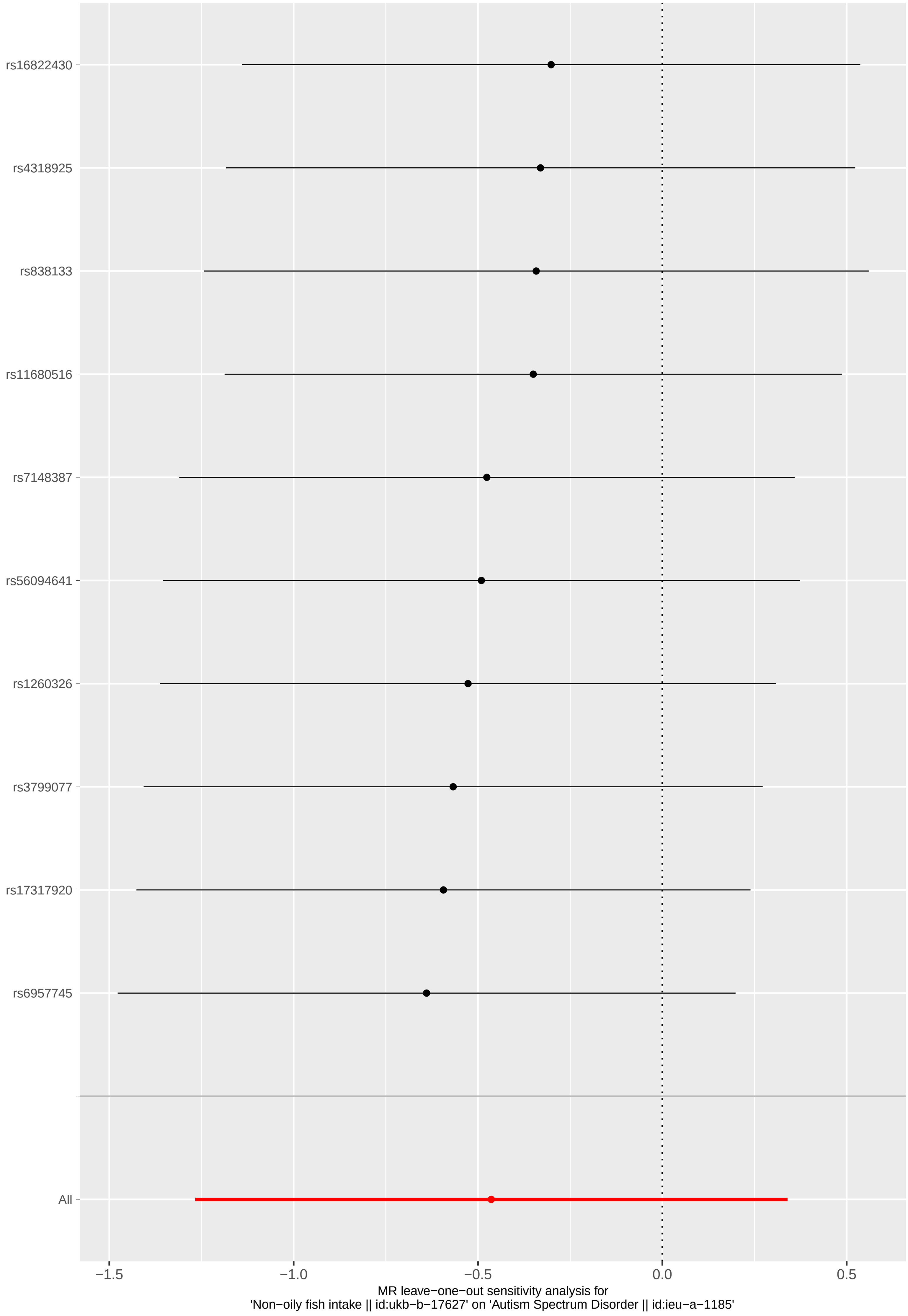

rs16822430
rs4318925
rs838133
rs11680516
rs7148387
rs56094641
rs1260326
rs3799077
rs17317920
rs6957745
All
−1.5
−1.0
−0.5
0.0
0.5
MR leave−one−out sensitivity analysis for
'Non−oily fish intake || id:ukb−b−17627' on 'Autism Spectrum Disorder || id:ieu−a−1185'

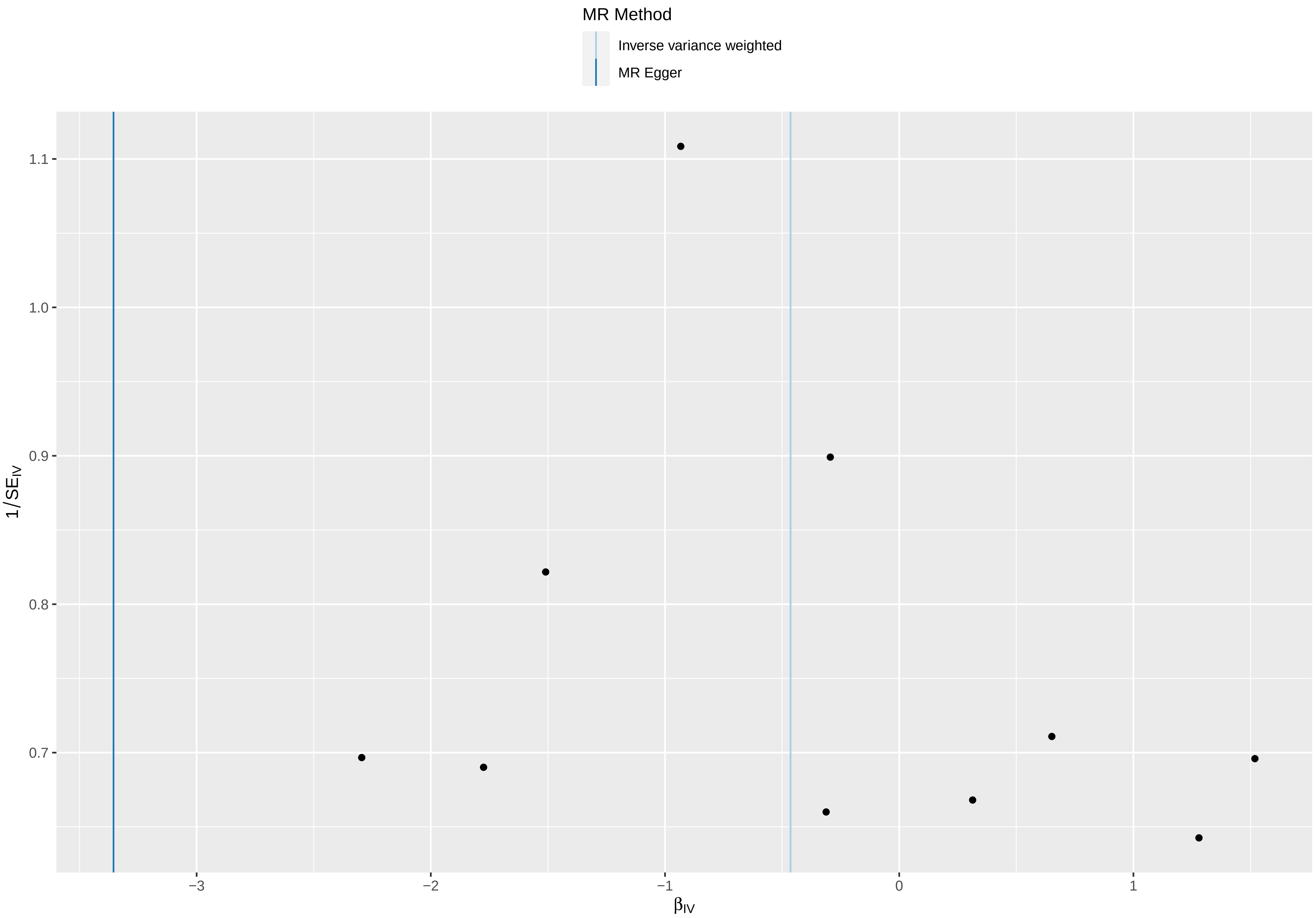
MR Method
Inverse variance weighted
MR Egger
1.1
1.0
0.9
0.8
0.7
1/SE_IV
−3
−2
−1
0
1
β_IV

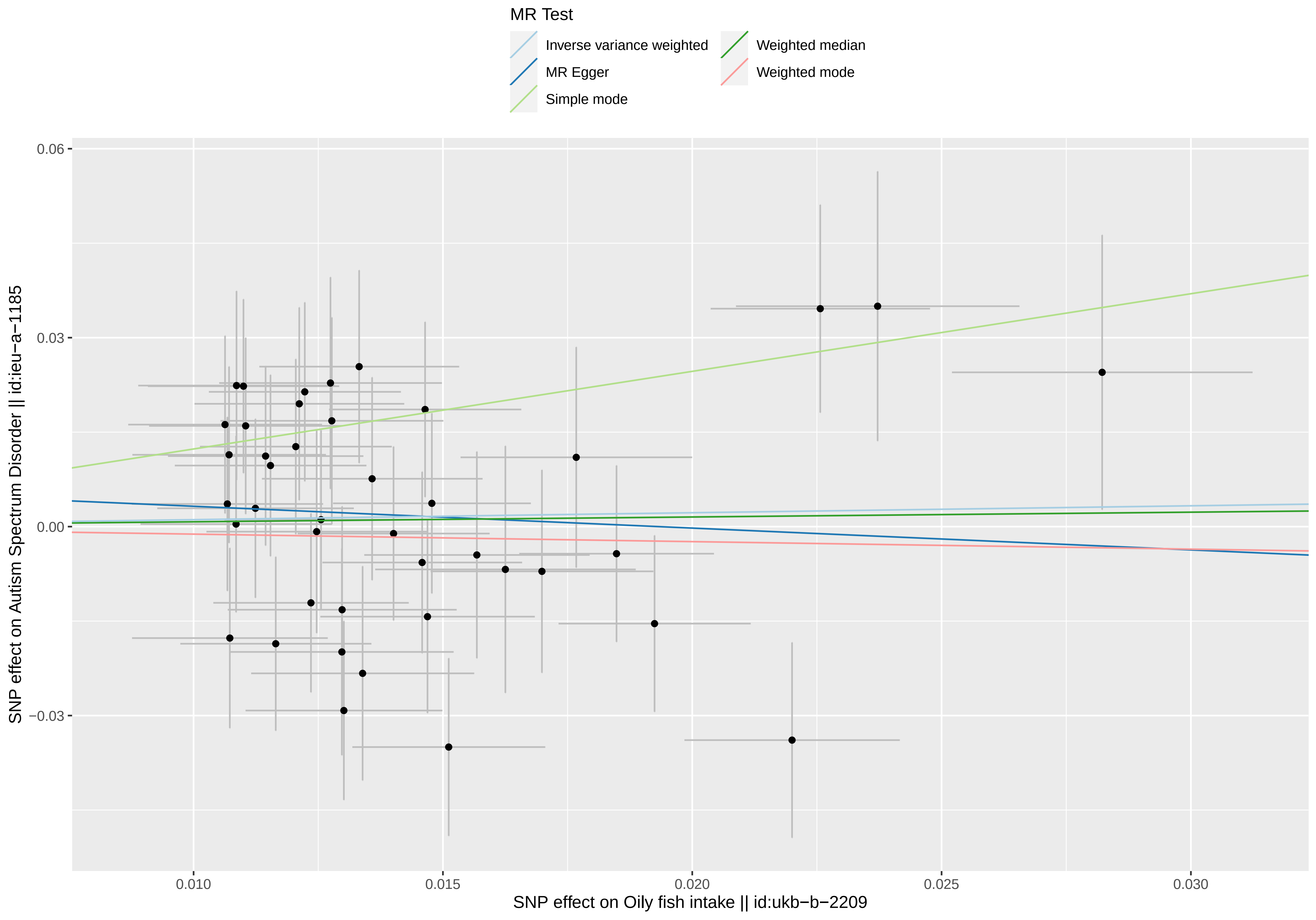
MR Test
Inverse variance weighted
MR Egger
Simple mode
Weighted median
Weighted mode
0.06
0.03
0.00
−0.03
0.010
0.015
0.020
0.025
0.030
SNP effect on Autism Spectrum Disorder || id:ieu−a−1185
SNP effect on Oily fish intake || id:ukb−b−2209

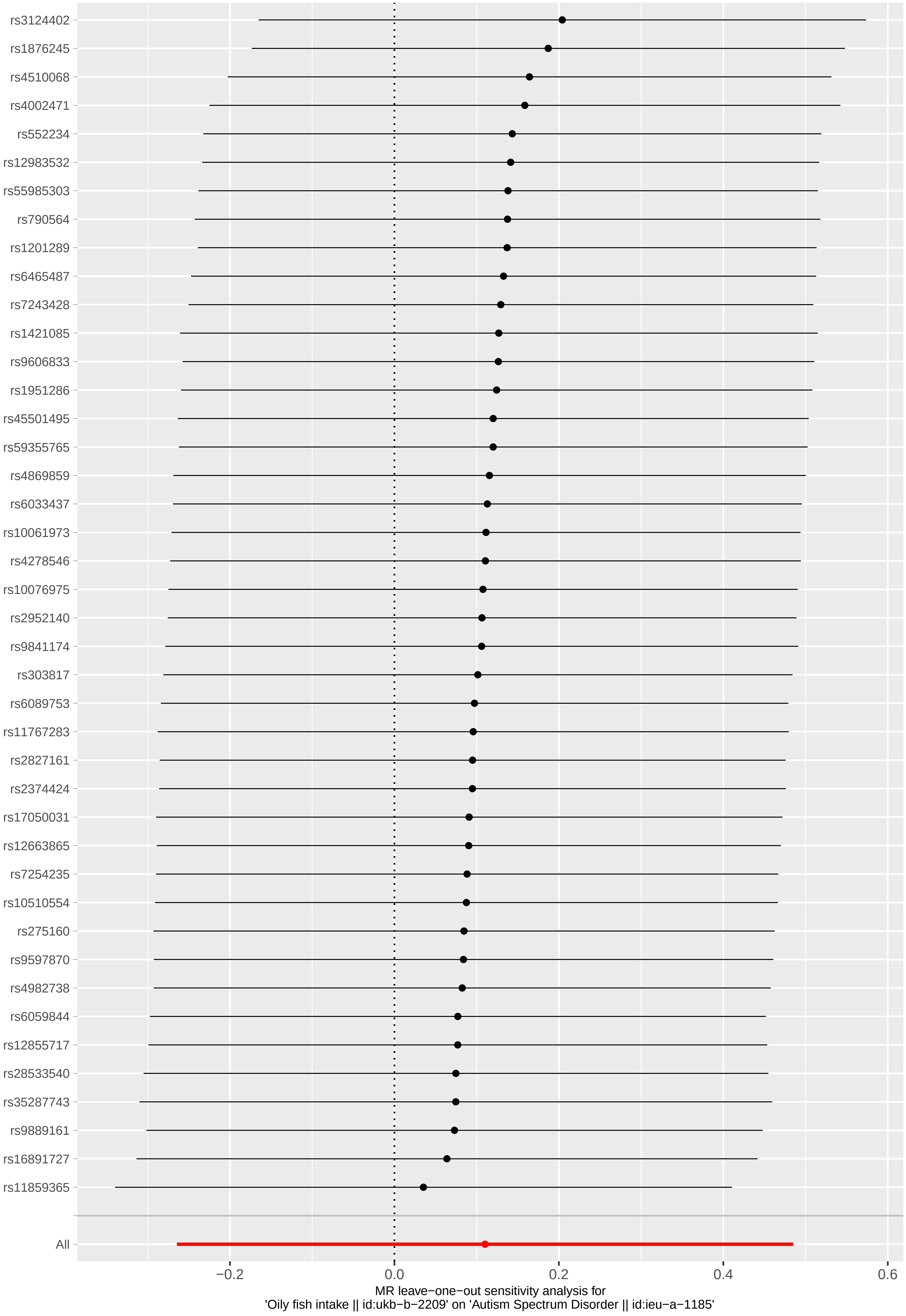
rs3124402
rs1876245
rs4510068
rs4002471
rs552234
rs12983532
rs55985303
rs790564
rs1201289
rs6465487
rs7243428
rs1421085
rs9606833
rs1951286
rs45501495
rs59355765
rs4869859
rs6033437
rs10061973
rs4278546
rs10076975
rs2952140
rs9841174
rs303817
rs6089753
rs11767283
rs2827161
rs2374424
rs17050031
rs12663865
rs7254235
rs10510554
rs275160
rs9597870
rs4982738
rs6059844
rs12855717
rs28533540
rs35287743
rs9889161
rs16891727
rs11859365
All
−0.2
0.0
0.2
0.4
0.6
MR leave−one−out sensitivity analysis for
'Oily fish intake || id:ukb−b−2209' on 'Autism Spectrum Disorder || id:ieu−a−1185'

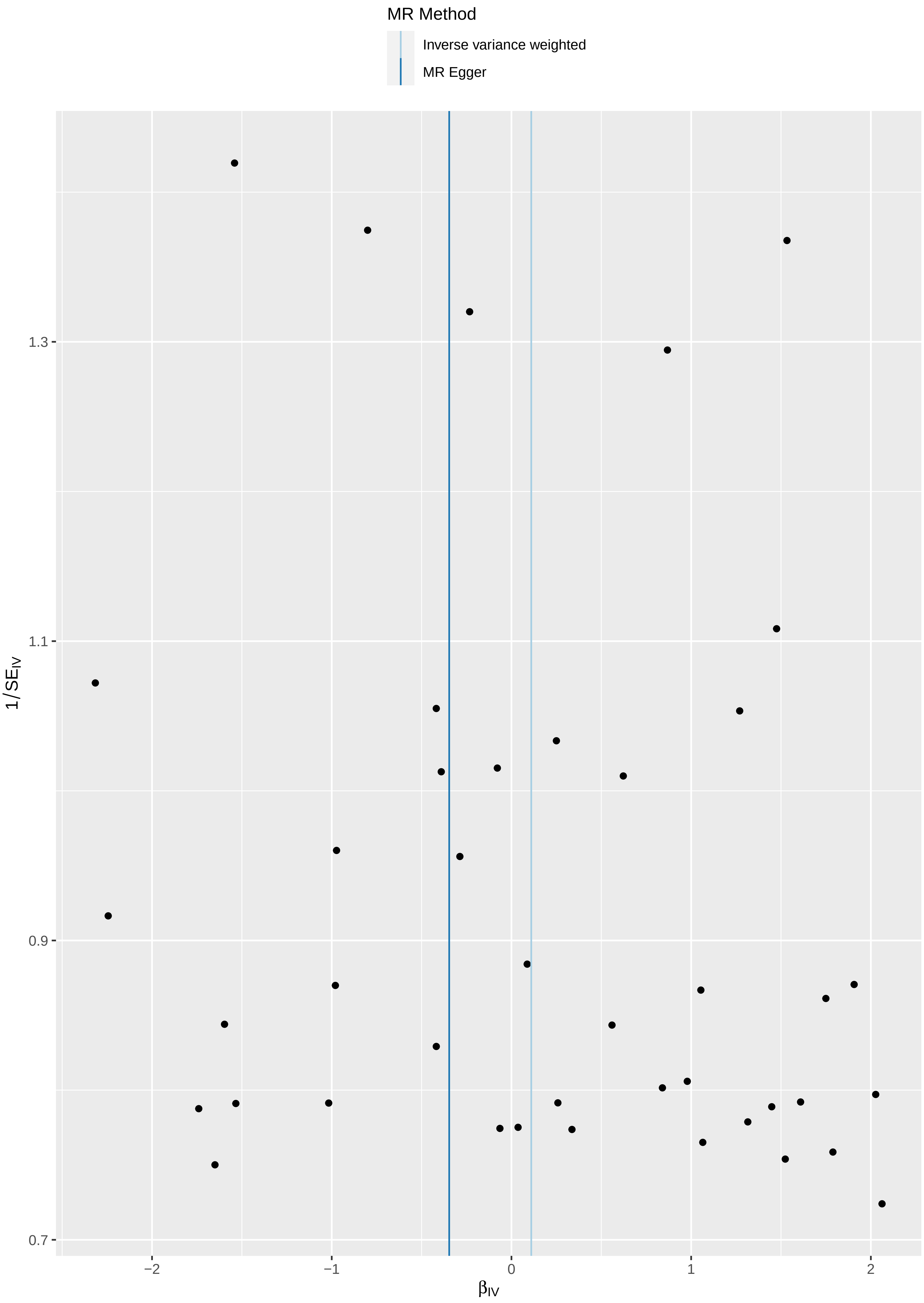
MR Method
Inverse variance weighted
MR Egger
1.3
1.1
0.9
0.7
1/SE$_{IV}$
−2
−1
0
1
2
β$_{IV}$

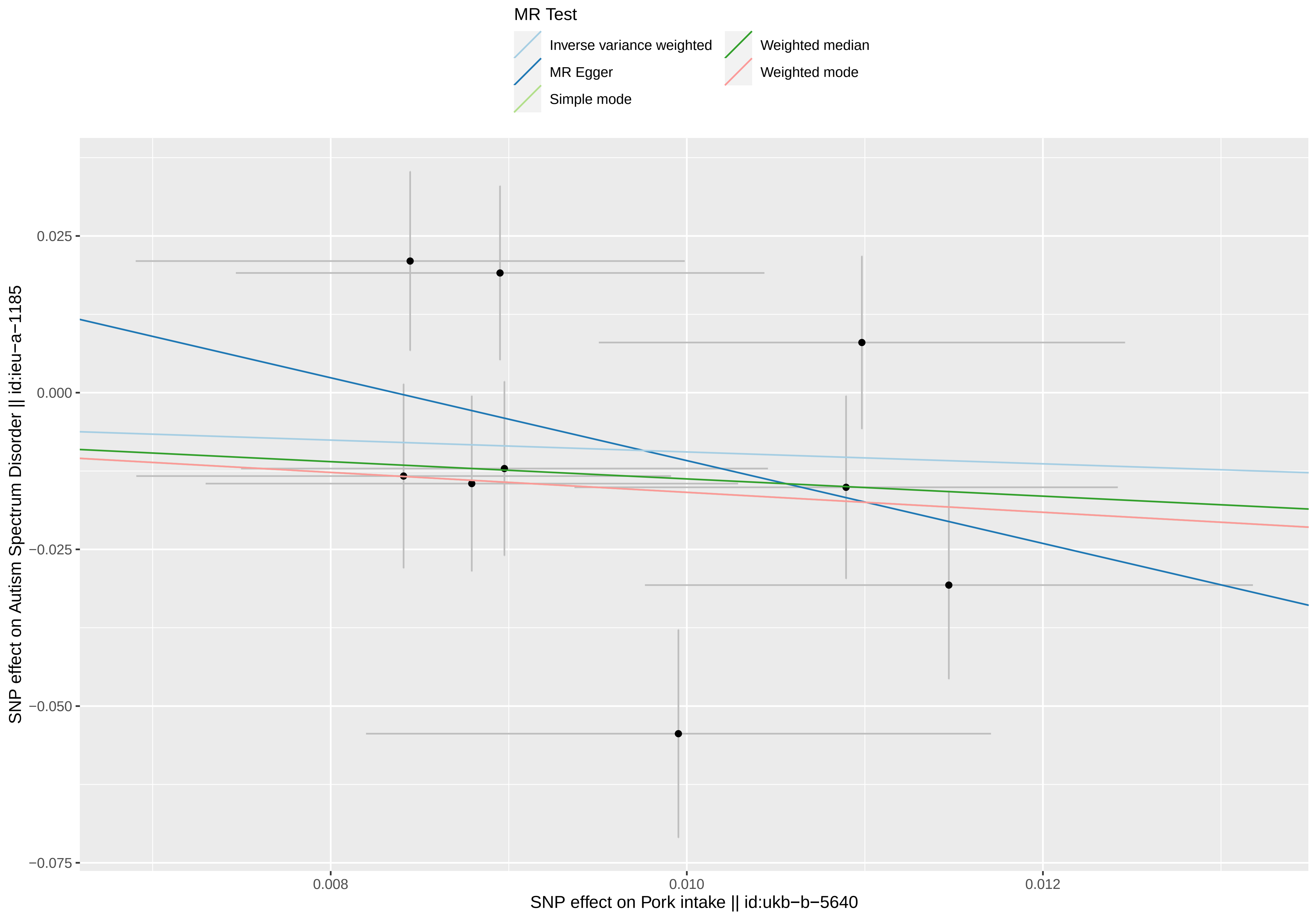

MR Test
Inverse variance weighted
MR Egger
Simple mode
Weighted median
Weighted mode
0.025
0.000
−0.025
−0.050
−0.075
0.008
0.010
0.012
SNP effect on Autism Spectrum Disorder || id:ieu−a−1185
SNP effect on Pork intake || id:ukb−b−5640

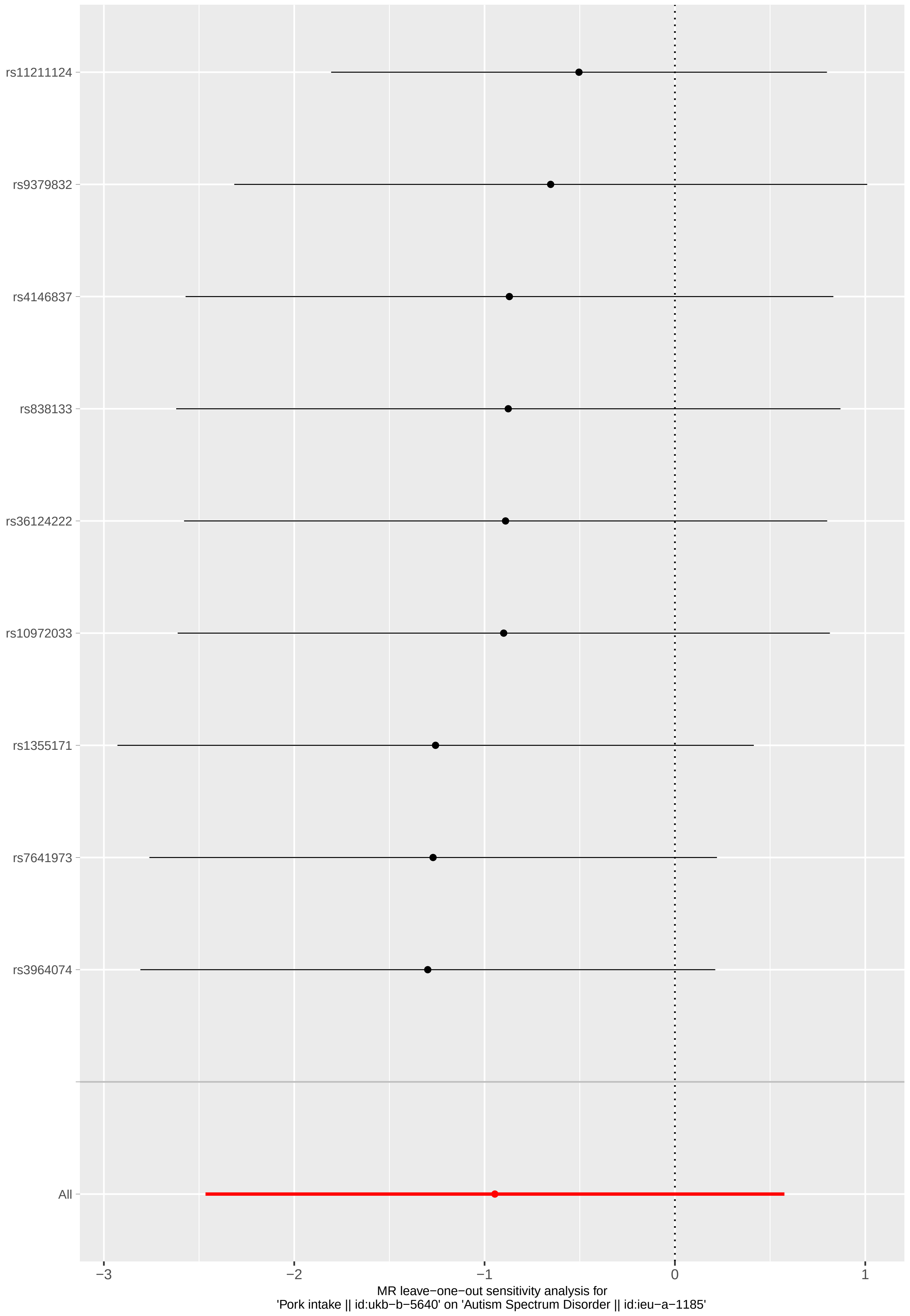
rs11211124
rs9379832
rs4146837
rs838133
rs36124222
rs10972033
rs1355171
rs7641973
rs3964074
All
−3
−2
−1
0
1
MR leave−one−out sensitivity analysis for
'Pork intake || id:ukb−b−5640' on 'Autism Spectrum Disorder || id:ieu−a−1185'

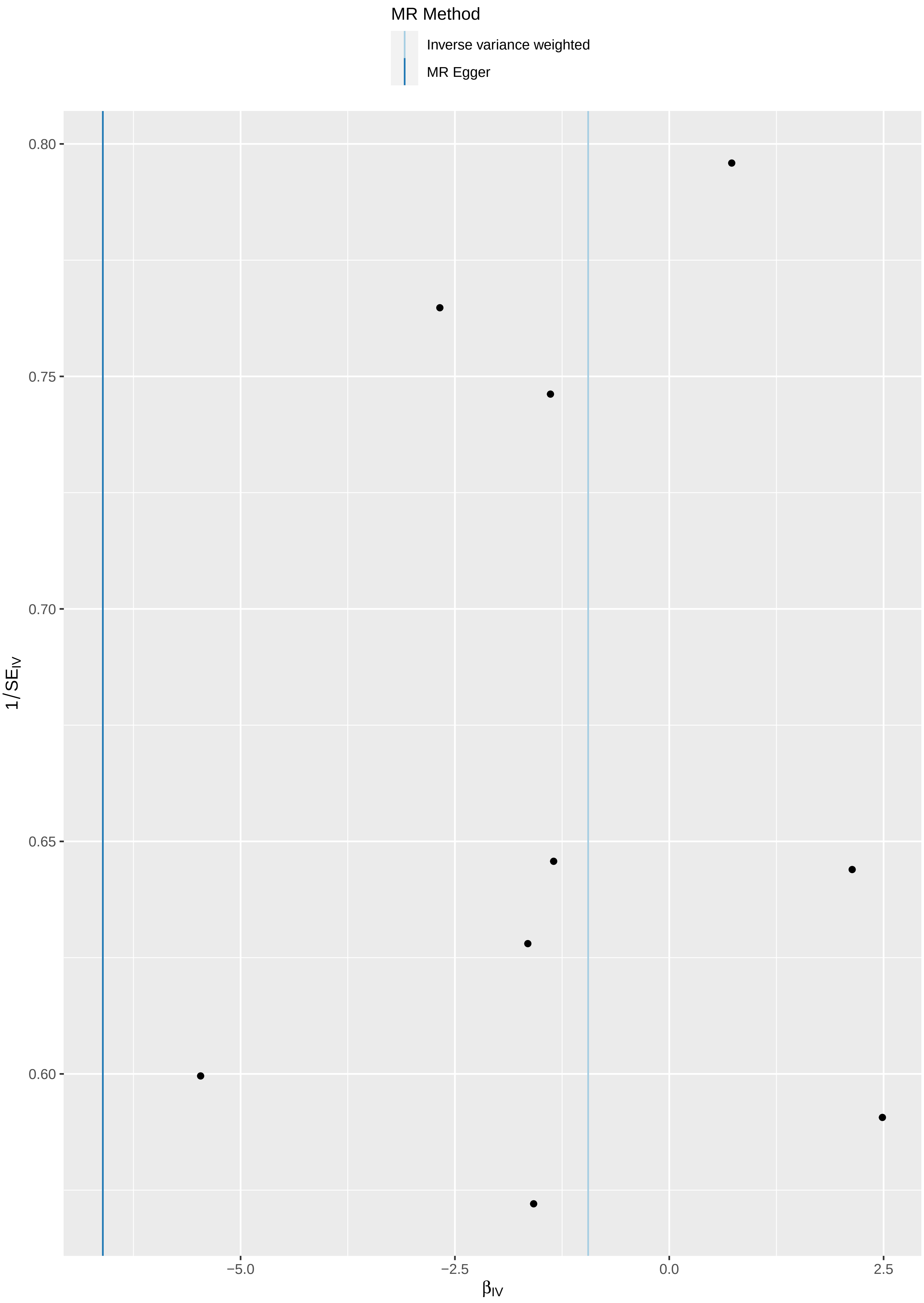
MR Method
Inverse variance weighted
MR Egger
0.80
0.75
0.70
0.65
0.60
1/SE_IV
−5.0
−2.5
0.0
2.5
β_IV

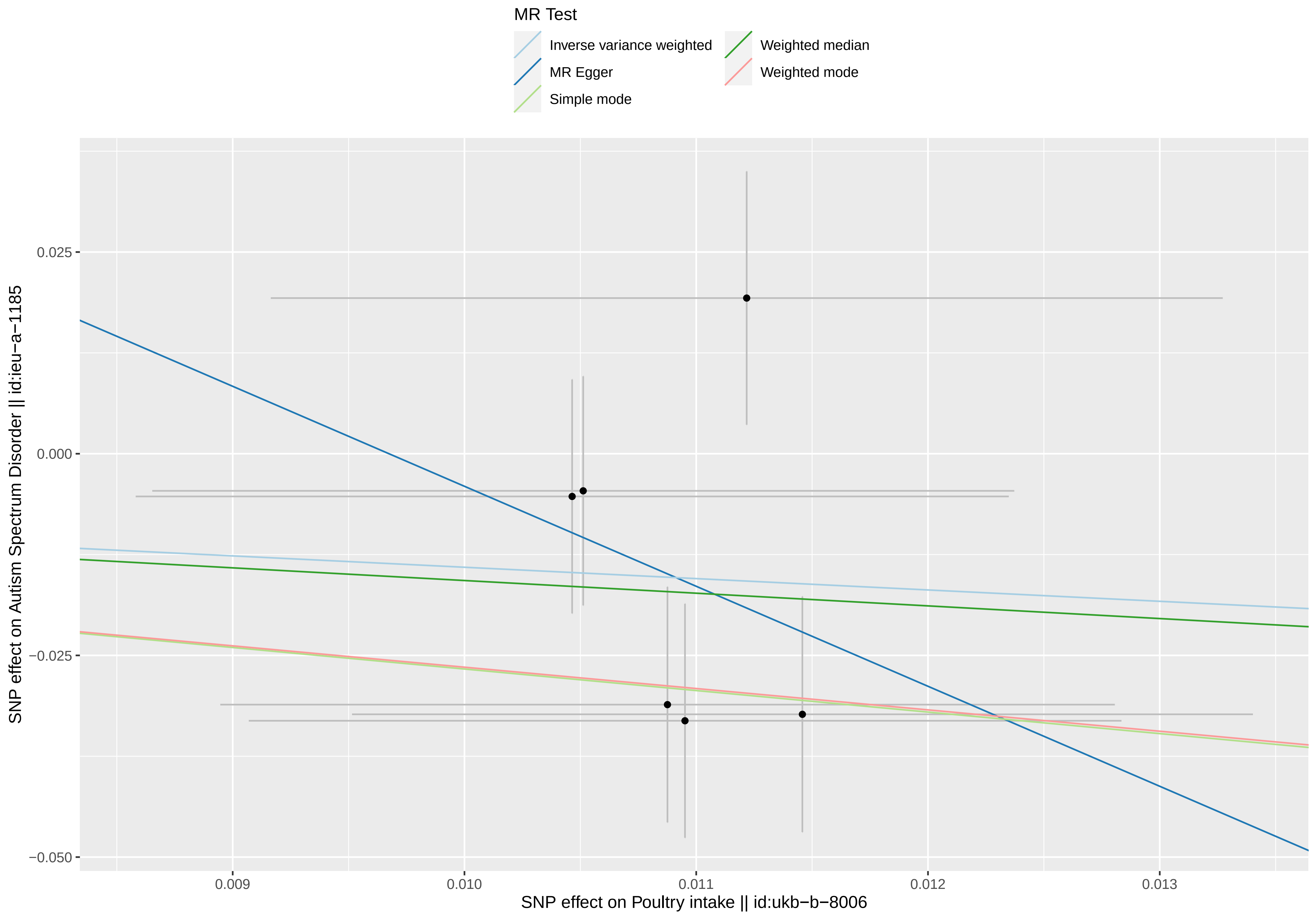

MR Test
Inverse variance weighted
MR Egger
Simple mode
Weighted median
Weighted mode
0.025
0.000
−0.025
−0.050
0.009
0.010
0.011
0.012
0.013
SNP effect on Autism Spectrum Disorder || id:ieu−a−1185
SNP effect on Poultry intake || id:ukb−b−8006

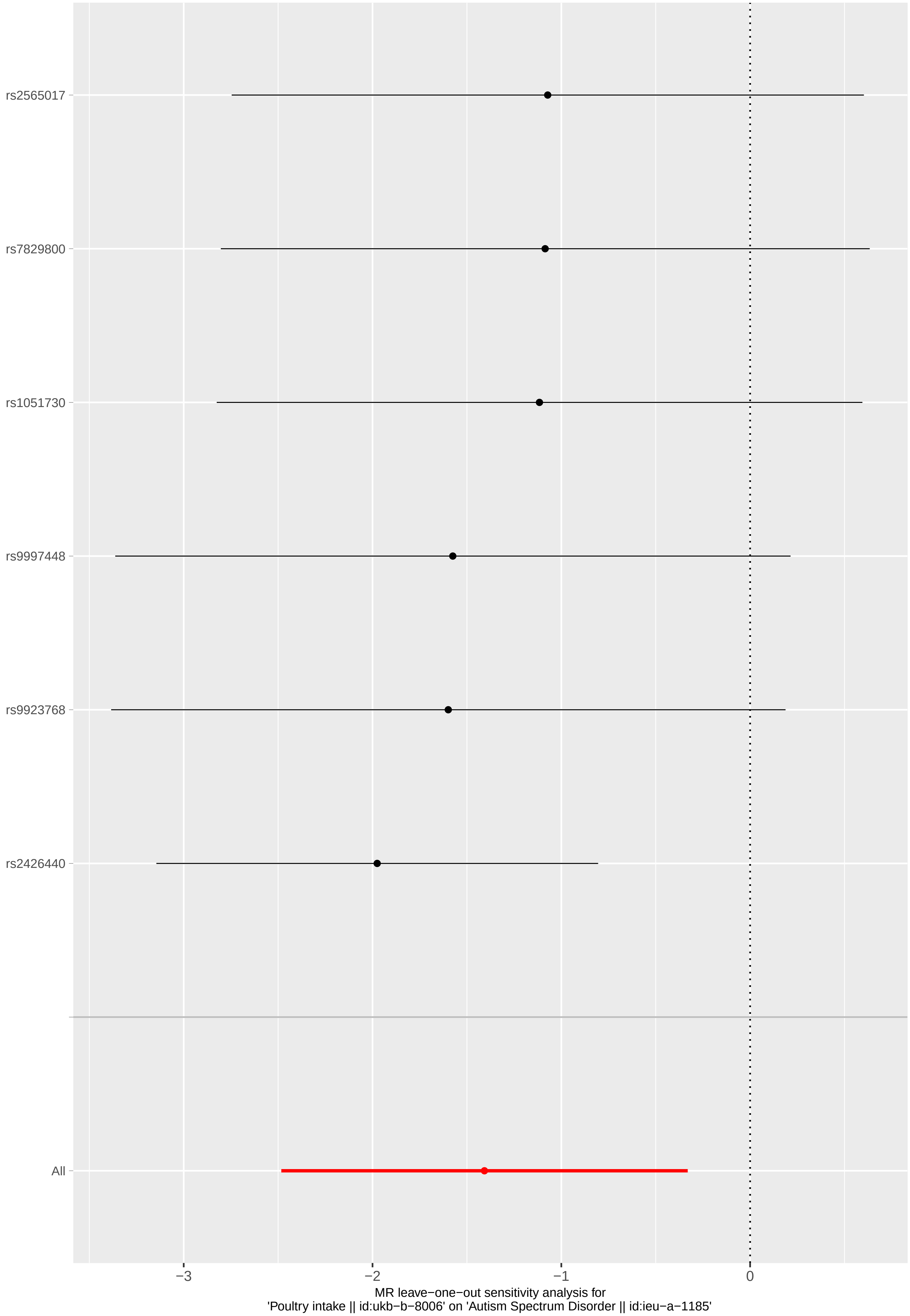
rs2565017
rs7829800
rs1051730
rs9997448
rs9923768
rs2426440
All
−3
−2
−1
0
MR leave−one−out sensitivity analysis for
'Poultry intake || id:ukb−b−8006' on 'Autism Spectrum Disorder || id:ieu−a−1185'

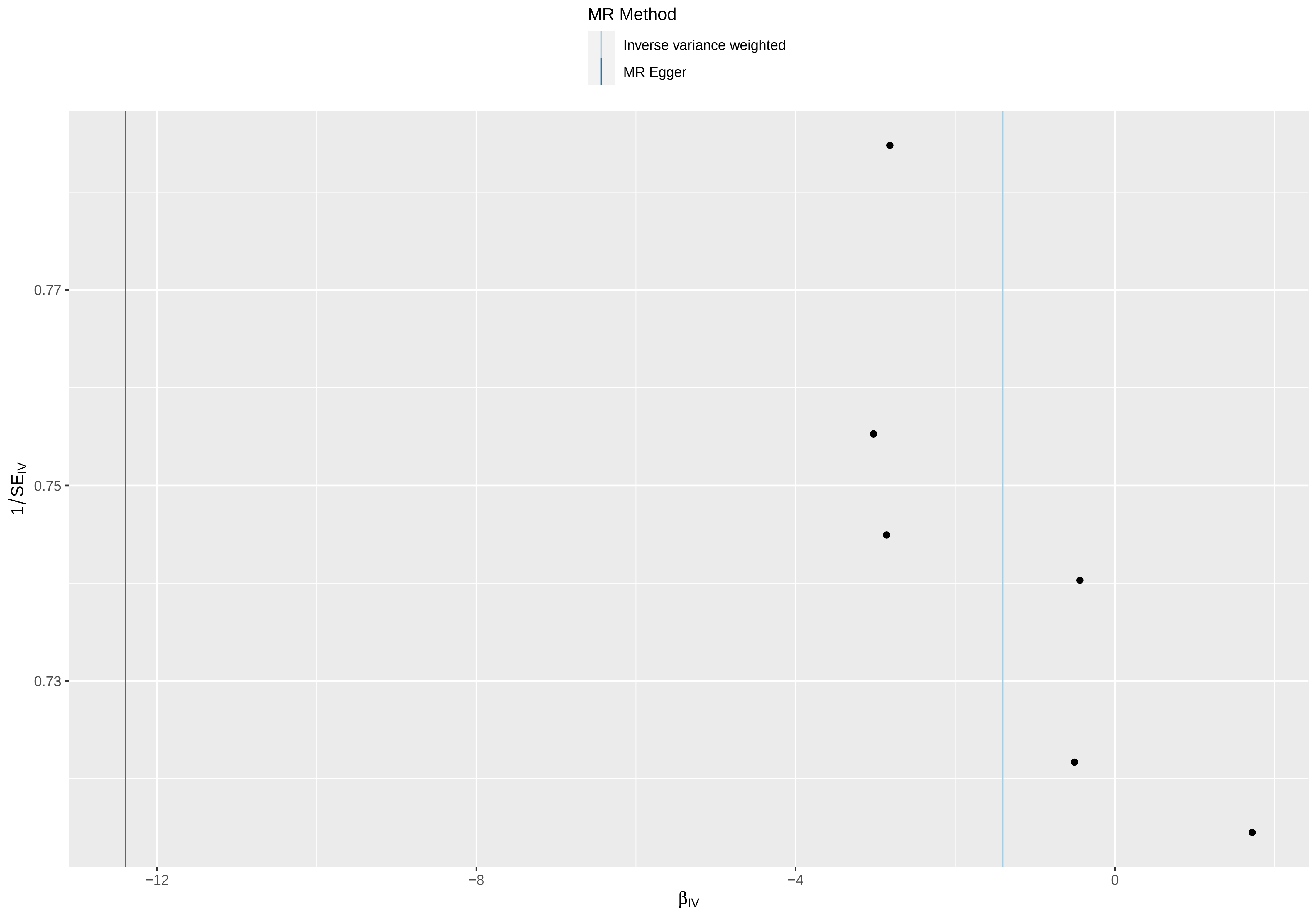

MR Method
Inverse variance weighted
MR Egger
0.77
0.75
0.73
1/SE_IV
−12
−8
−4
0
β_IV

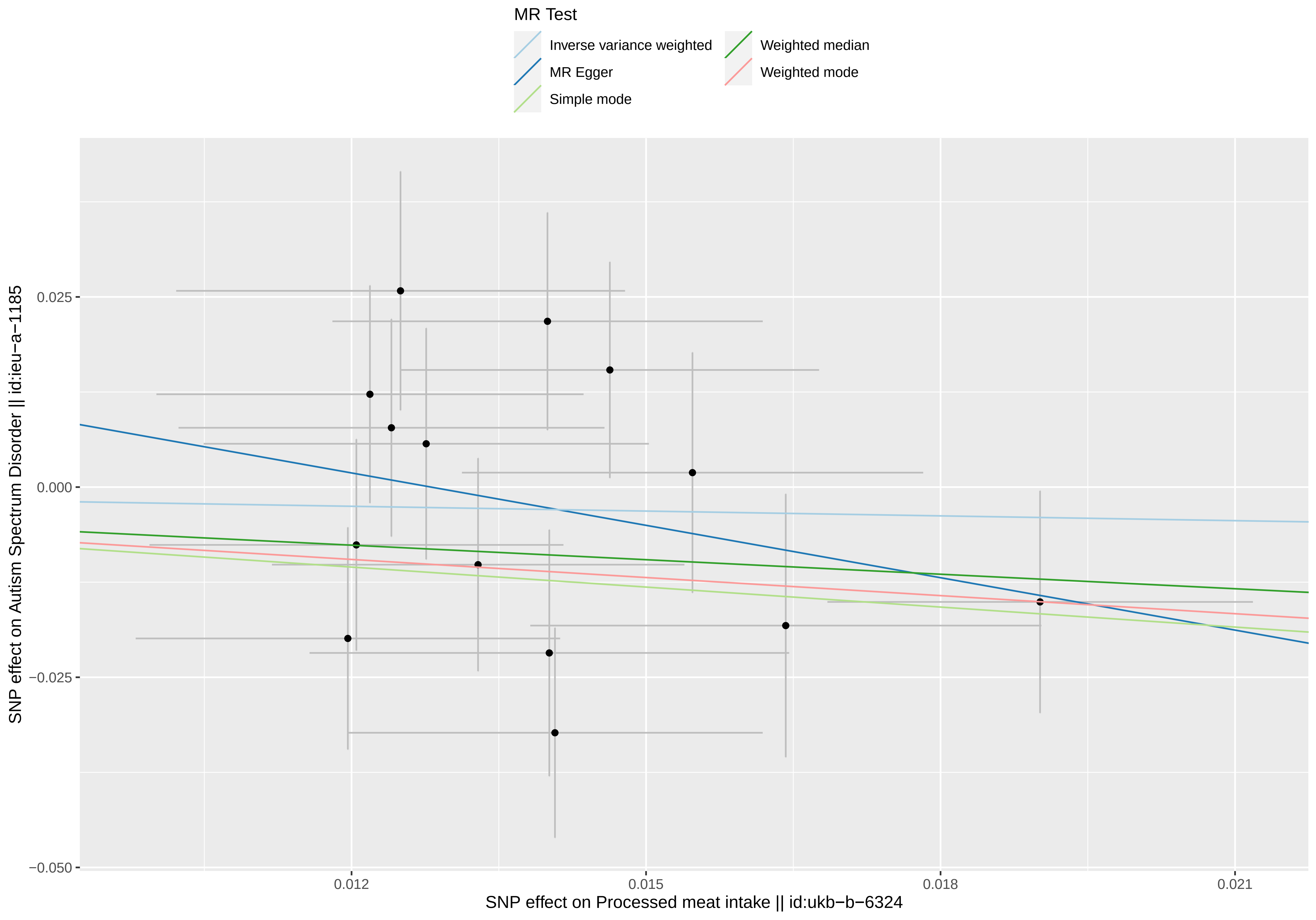
MR Test
Inverse variance weighted
MR Egger
Simple mode
Weighted median
Weighted mode
SNP effect on Autism Spectrum Disorder || id:ieu−a−1185
0.025
0.000
−0.025
−0.050
0.012
0.015
0.018
0.021
SNP effect on Processed meat intake || id:ukb−b−6324

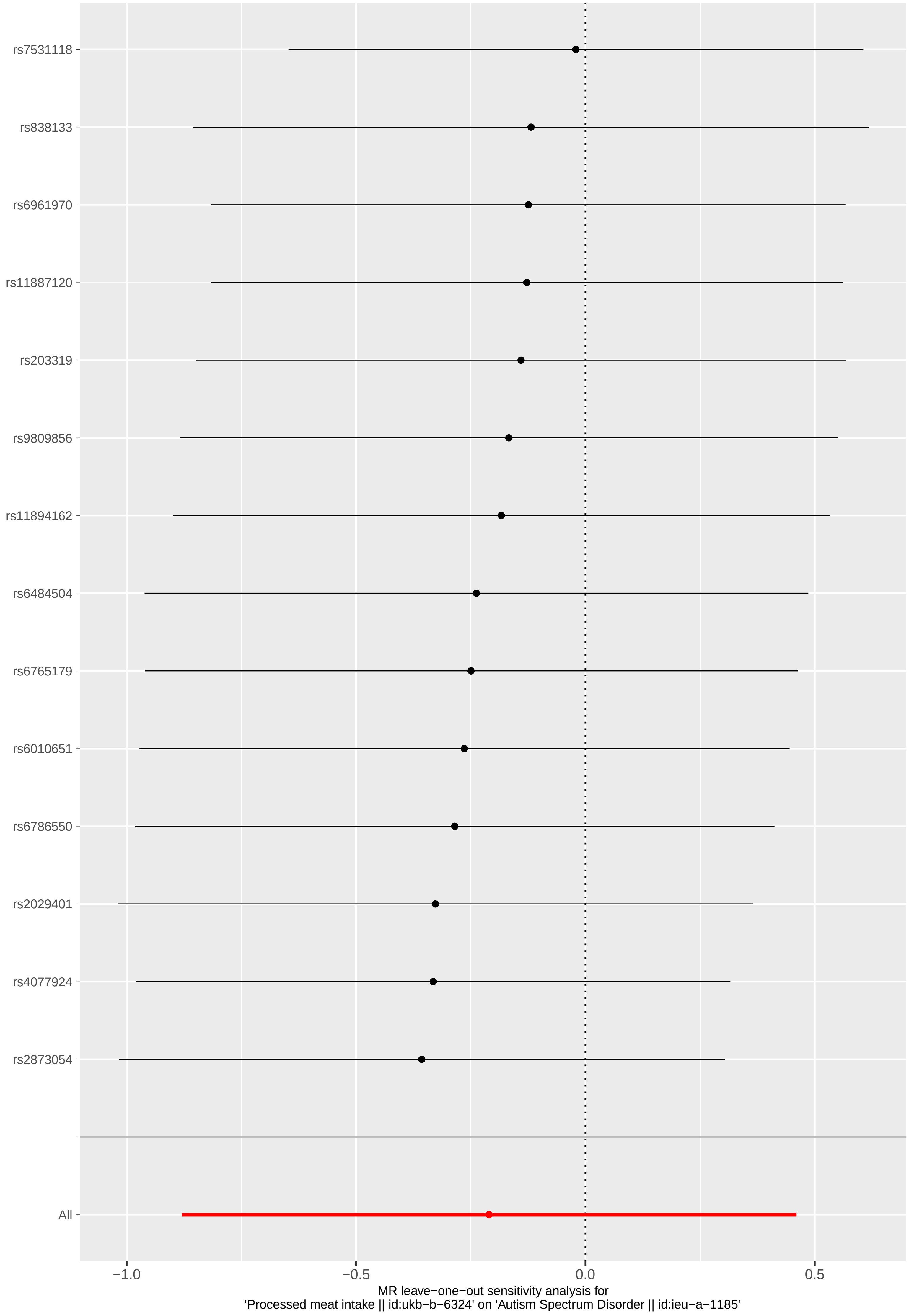
rs7531118
rs838133
rs6961970
rs11887120
rs203319
rs9809856
rs11894162
rs6484504
rs6765179
rs6010651
rs6786550
rs2029401
rs4077924
rs2873054
All
−1.0
−0.5
0.0
0.5
MR leave−one−out sensitivity analysis for
'Processed meat intake || id:ukb−b−6324' on 'Autism Spectrum Disorder || id:ieu−a−1185'

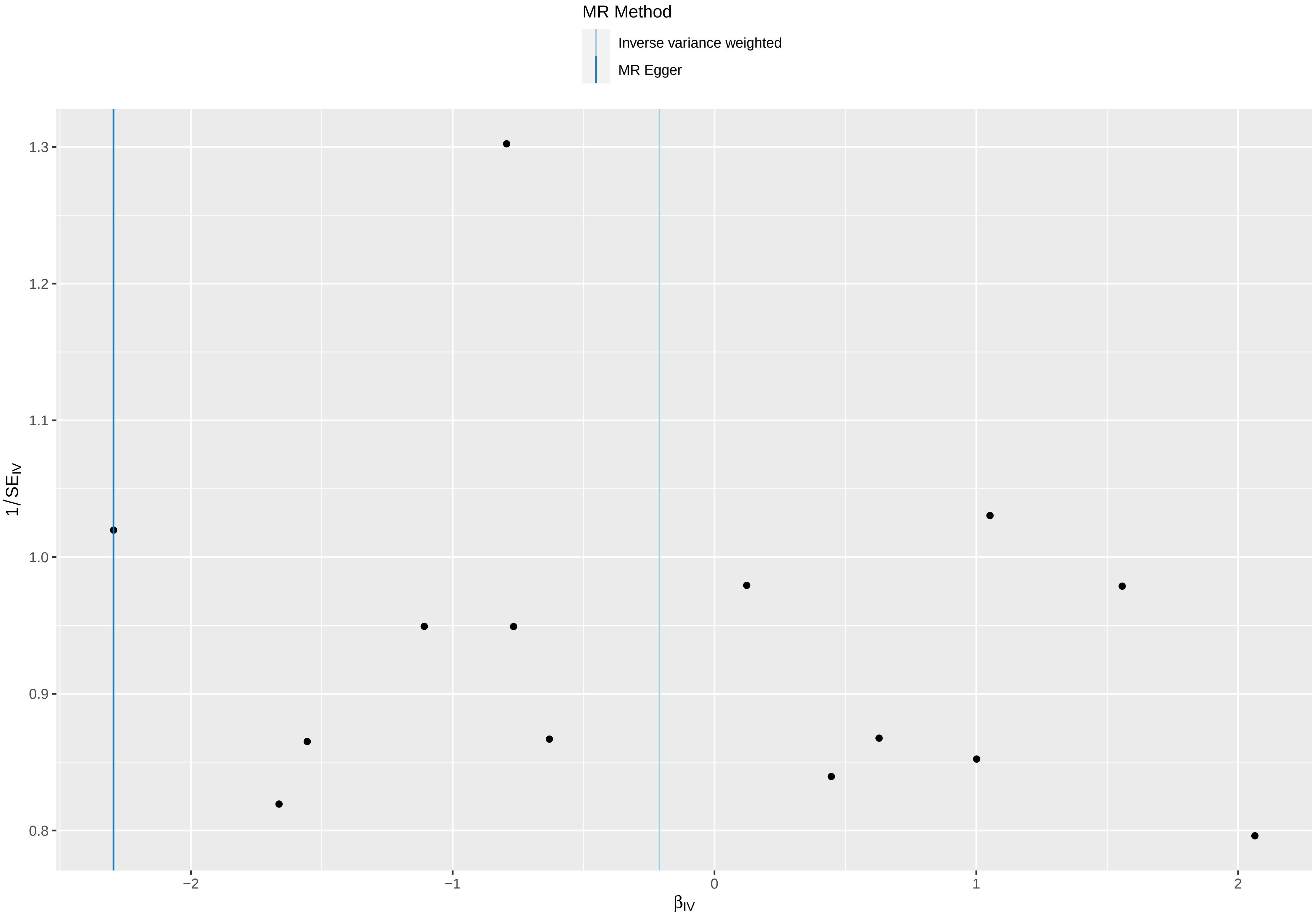

MR Method
Inverse variance weighted
MR Egger
1.3
1.2
1.1
1.0
0.9
0.8
$1/SE_{IV}$
−2
−1
0
1
2
β_{IV}

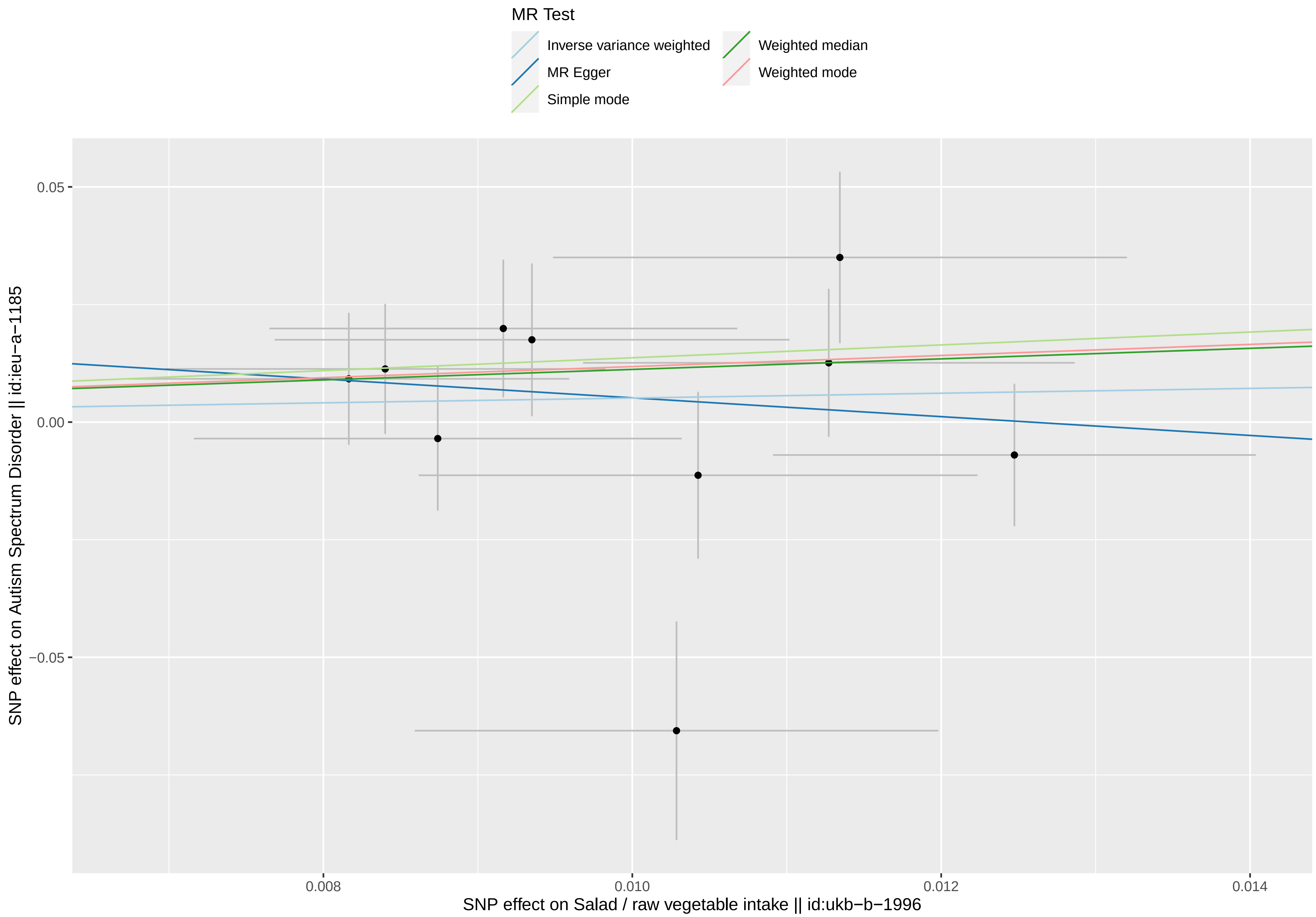
MR Test
Inverse variance weighted
MR Egger
Simple mode
Weighted median
Weighted mode
0.05
0.00
−0.05
0.008
0.010
0.012
0.014
SNP effect on Salad / raw vegetable intake || id:ukb−b−1996
SNP effect on Autism Spectrum Disorder || id:ieu−a−1185

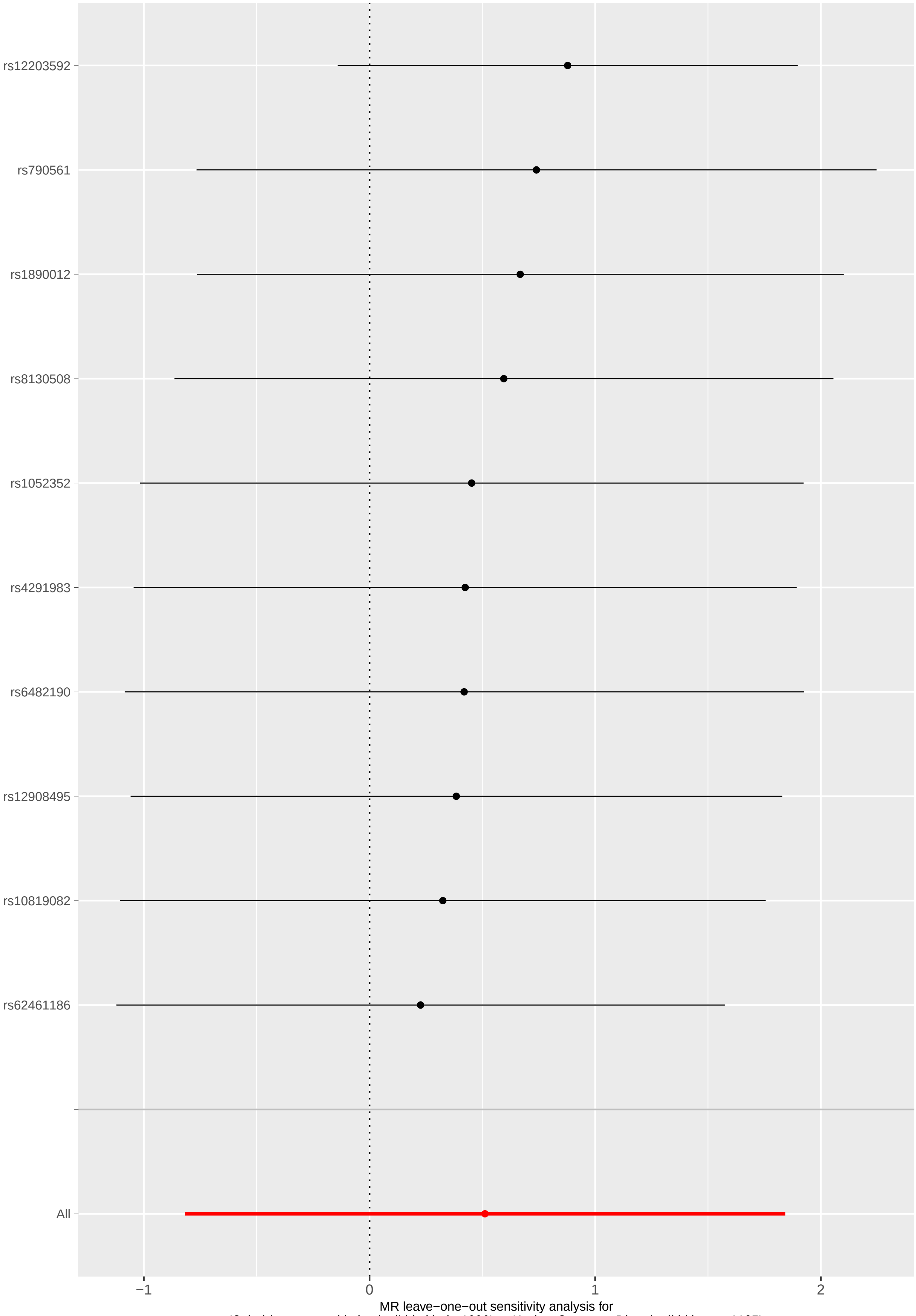

MR leave−one−out sensitivity analysis for
'Salad / raw vegetable intake || id:ukb−b−1996' on 'Autism Spectrum Disorder || id:ieu−a−1185'

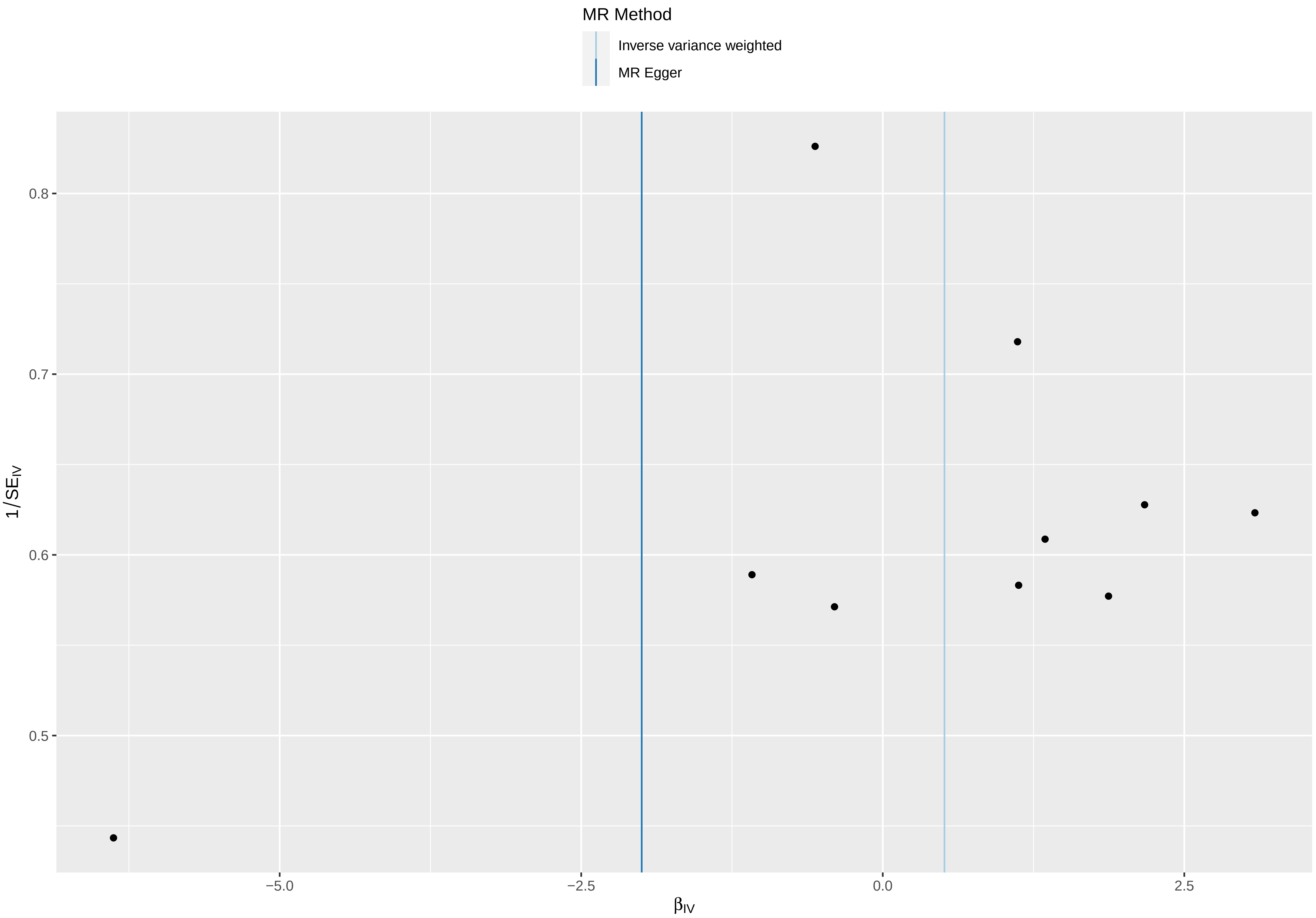

MR Method
Inverse variance weighted
MR Egger
0.8
0.7
0.6
0.5
1/SE$_{IV}$
−5.0
−2.5
0.0
2.5
β$_{IV}$

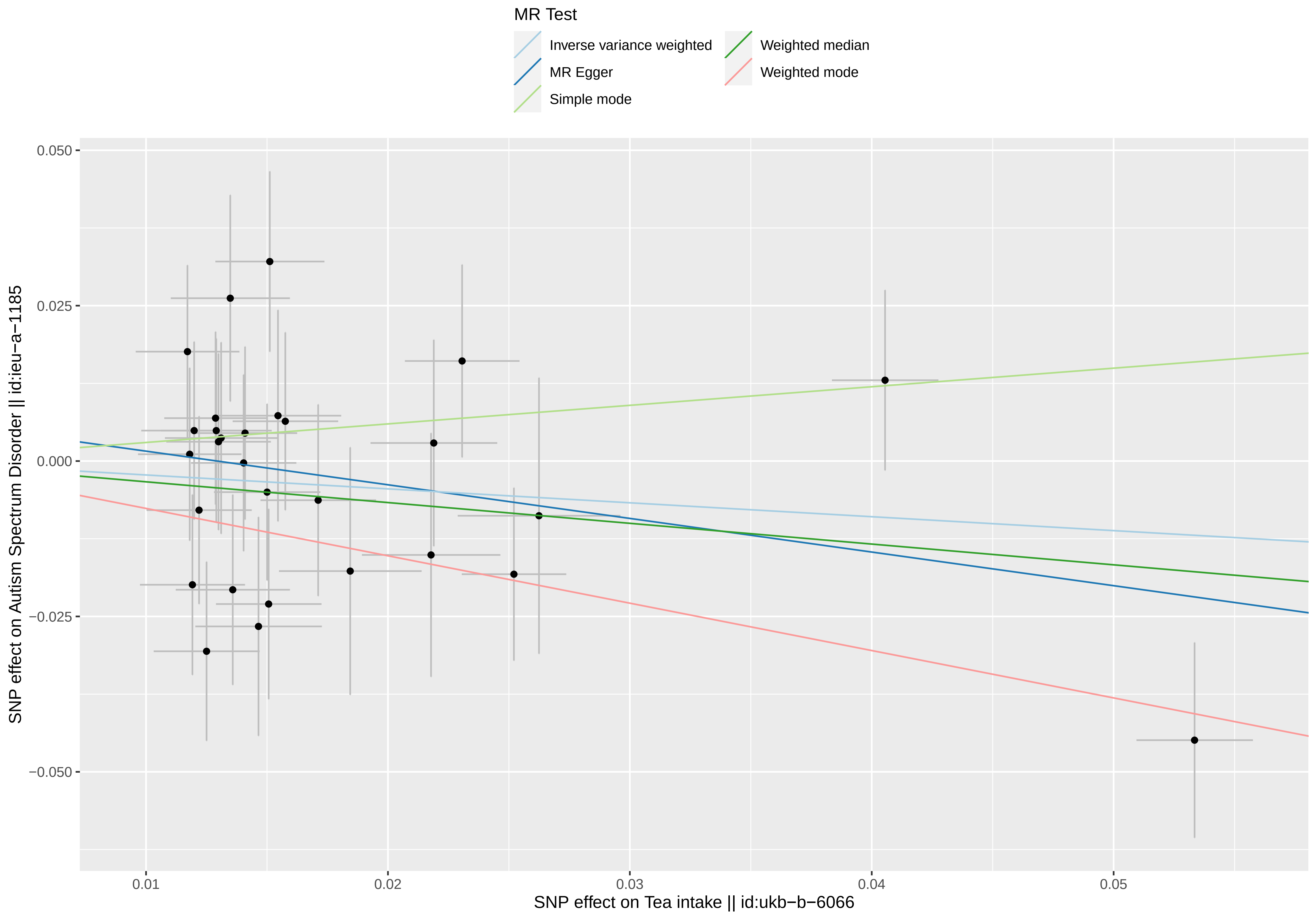
MR Test
Inverse variance weighted
MR Egger
Simple mode
Weighted median
Weighted mode
0.050
0.025
0.000
−0.025
−0.050
SNP effect on Autism Spectrum Disorder || id:ieu−a−1185
0.01
0.02
0.03
0.04
0.05
SNP effect on Tea intake || id:ukb−b−6066

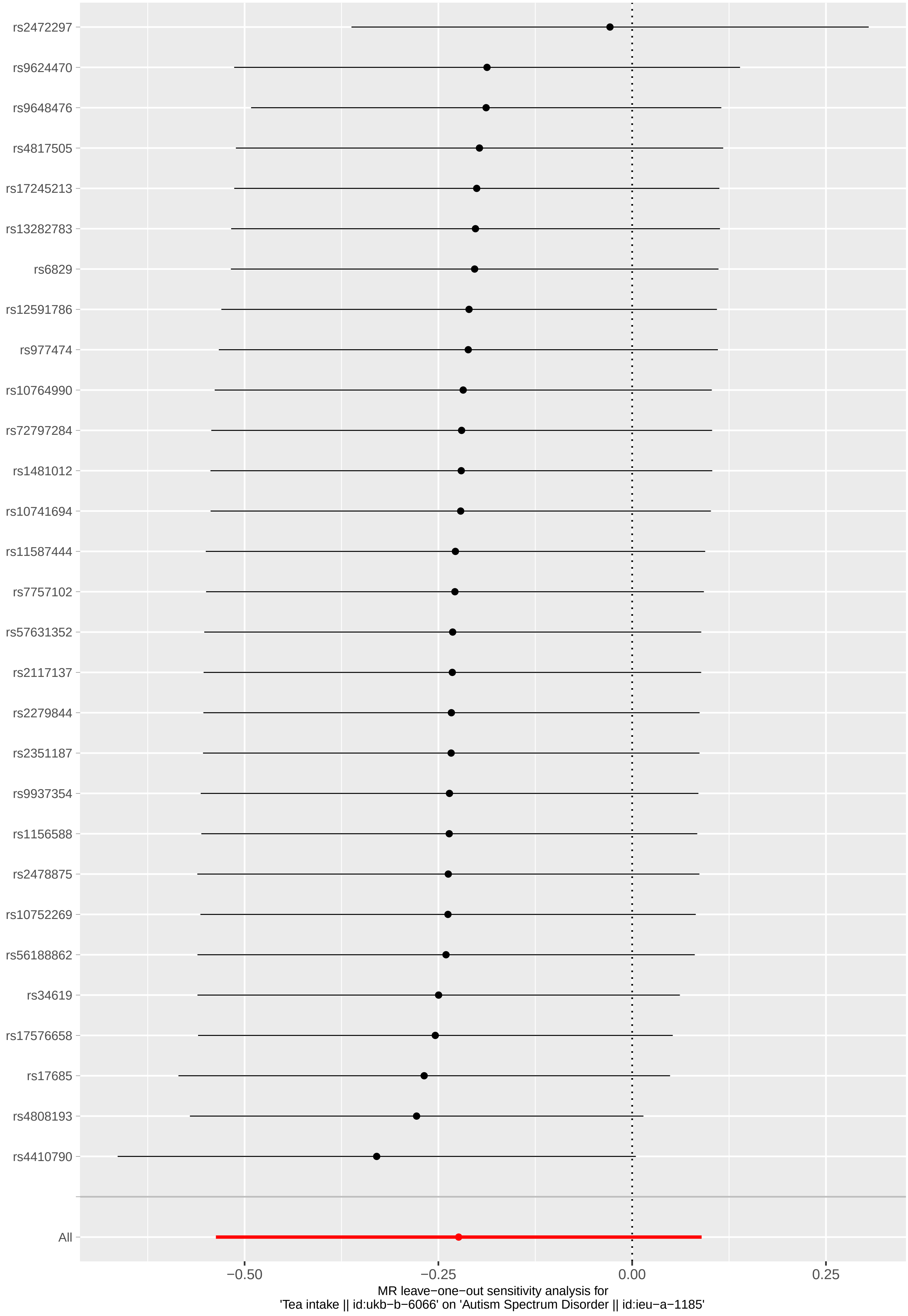
rs2472297
rs9624470
rs9648476
rs4817505
rs17245213
rs13282783
rs6829
rs12591786
rs977474
rs10764990
rs72797284
rs1481012
rs10741694
rs11587444
rs7757102
rs57631352
rs2117137
rs2279844
rs2351187
rs9937354
rs1156588
rs2478875
rs10752269
rs56188862
rs34619
rs17576658
rs17685
rs4808193
rs4410790
All
−0.50
−0.25
0.00
0.25
MR leave−one−out sensitivity analysis for
'Tea intake || id:ukb−b−6066' on 'Autism Spectrum Disorder || id:ieu−a−1185'

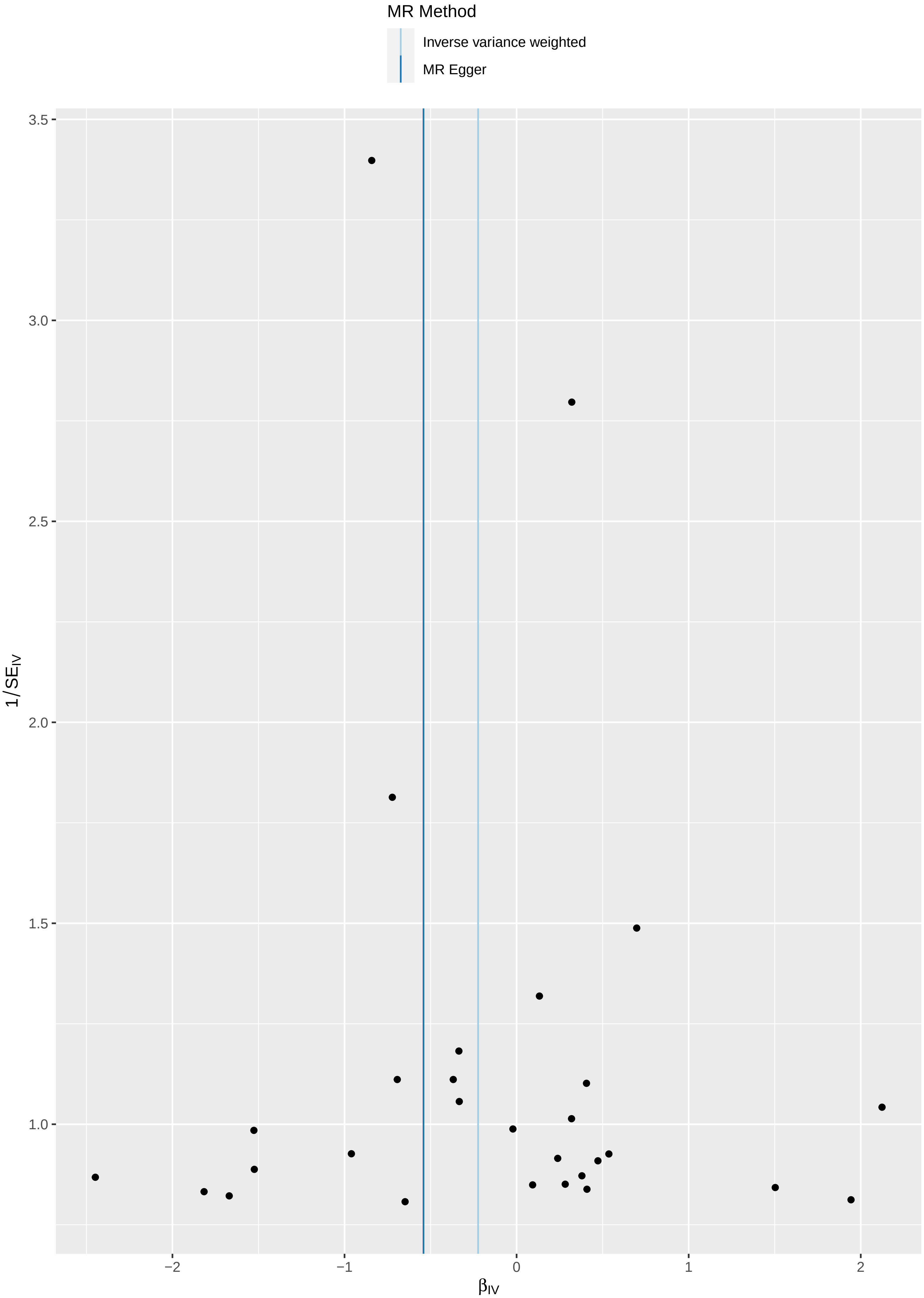

MR Method
Inverse variance weighted
MR Egger
3.5
3.0
2.5
2.0
1.5
1.0
1/SE_IV
−2
−1
0
1
2
β_IV

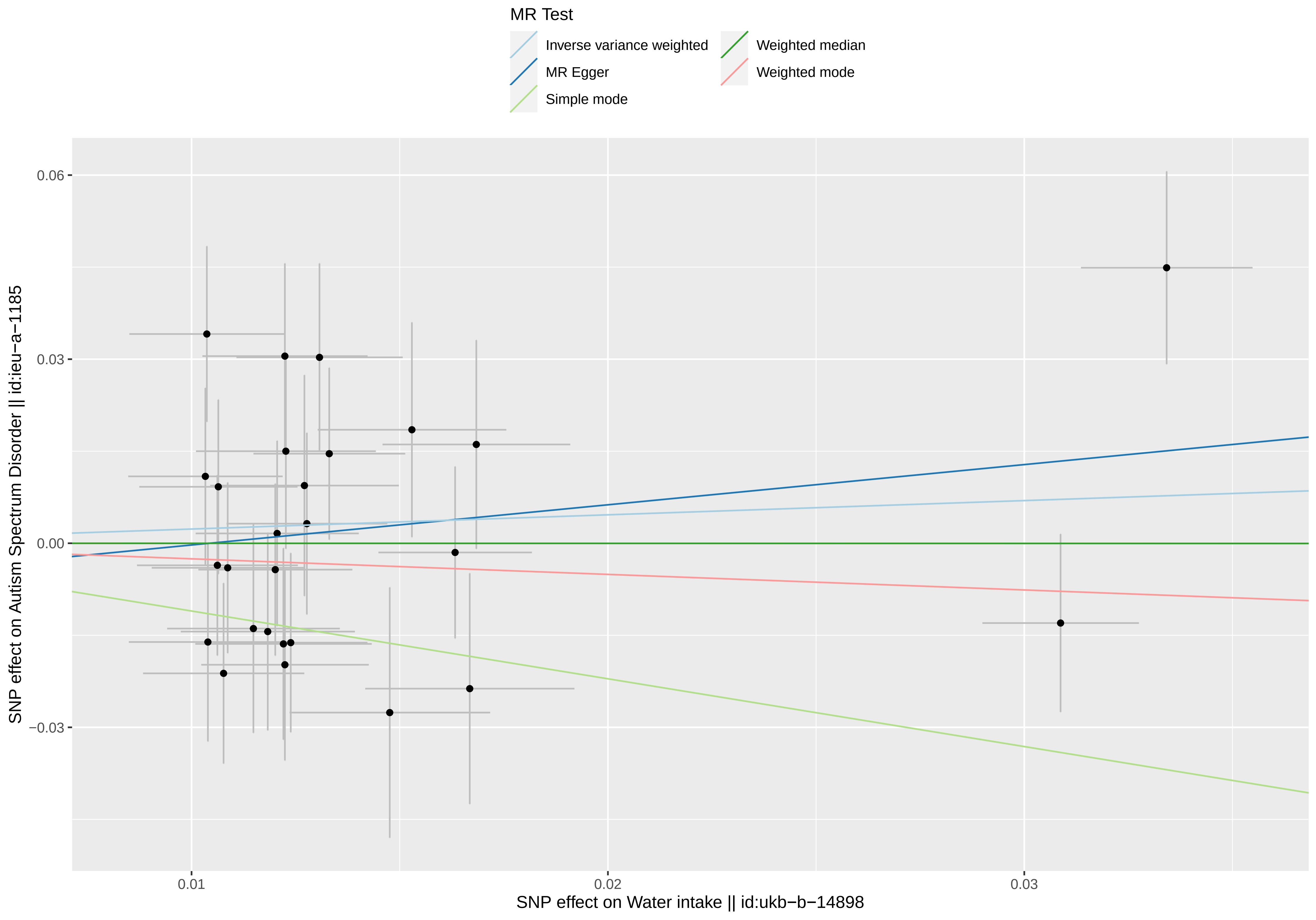
MR Test
Inverse variance weighted
MR Egger
Simple mode
Weighted median
Weighted mode
0.06
0.03
0.00
−0.03
0.01
0.02
0.03
SNP effect on Autism Spectrum Disorder || id:ieu−a−1185
SNP effect on Water intake || id:ukb−b−14898

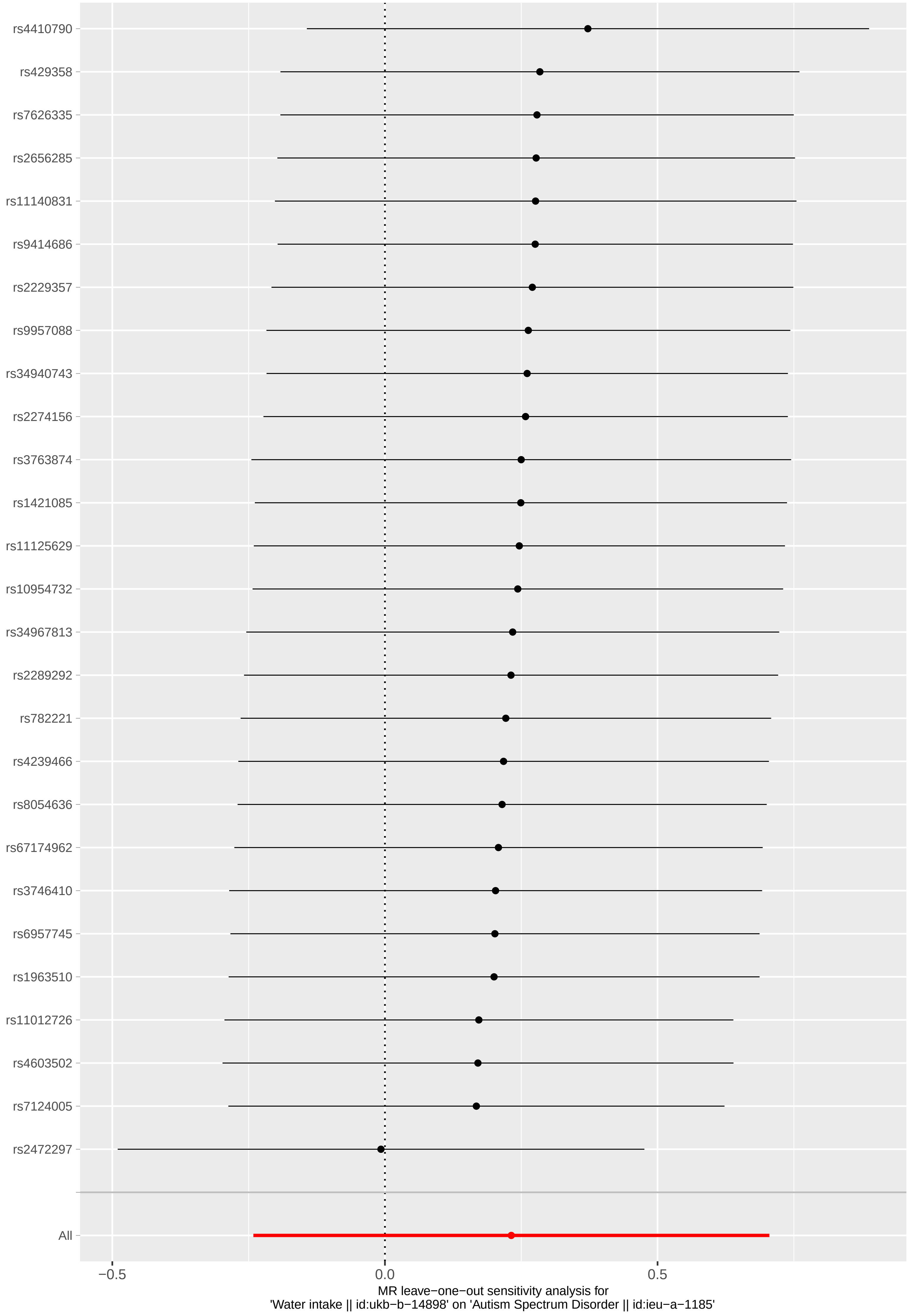
rs4410790
rs429358
rs7626335
rs2656285
rs11140831
rs9414686
rs2229357
rs9957088
rs34940743
rs2274156
rs3763874
rs1421085
rs11125629
rs10954732
rs34967813
rs2289292
rs782221
rs4239466
rs8054636
rs67174962
rs3746410
rs6957745
rs1963510
rs11012726
rs4603502
rs7124005
rs2472297
All
−0.5
0.0
0.5
MR leave−one−out sensitivity analysis for
'Water intake || id:ukb−b−14898' on 'Autism Spectrum Disorder || id:ieu−a−1185'

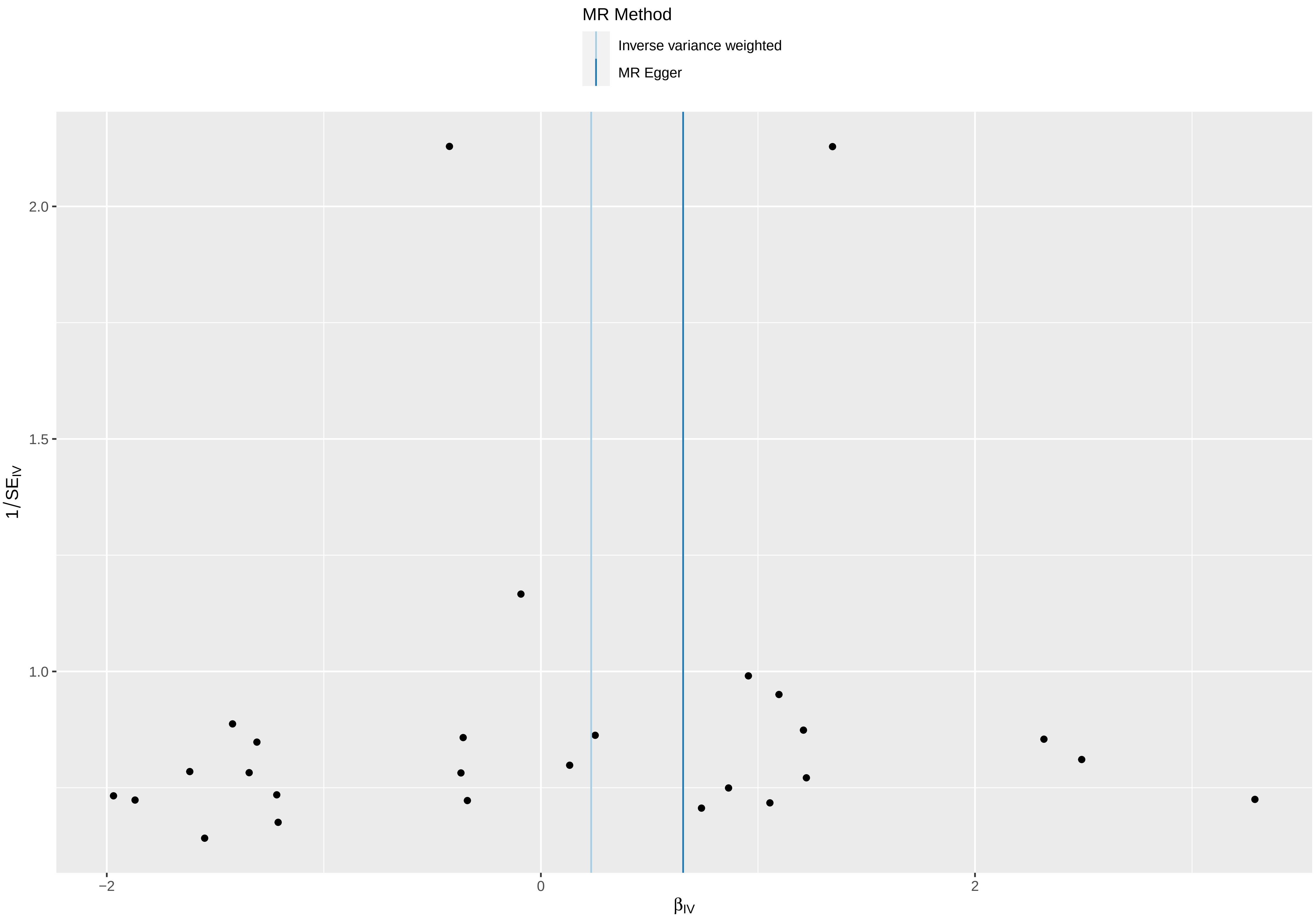

MR Method
Inverse variance weighted
MR Egger
2.0
1.5
1.0
$1/SE_{IV}$
−2
0
2
β_{IV}
