# Supplementary material for: Associations between genetically determined dietary factors and risk of autism spectrum disorder: a Mendelian randomization study
Source: Front Nutr. 2024 Mar 1;11:1210855. doi: 10.3389/fnut.2024.1210855 (PMC10940521; doi:10.3389/fnut.2024.1210855)
Supplement: Supplementary file 3 [file Data_Sheet_3.PDF]

STROBE-MR checklist of recommended items to address in reports of Mendelian randomization studies[1 2]

Item No.	Section		Checklist item	Page No.	Relevant text from manuscript
1	**TITLE and ABSTRACT**		Indicate Mendelian randomization (MR) as the study's design in the title and/or the abstract if that is a main purpose of the study	1	Mendelian randomization
	**INTRODUCTION**				
2	**Background**		Explain the scientific background and rationale for the reported study. What is the exposure? Is a potential causal relationship between exposure and outcome plausible? Justify why MR is a helpful method to address the study question	2,3	Therefore, MR is an ideal approach for investigating causal relationships between dietary factors and ASD.
3	**Objectives**		State specific objectives clearly, including pre-specified causal hypotheses (if any). State that MR is a method that, under specific assumptions, intends to estimate causal effects	3	This study uses MR methods to uncover the intrinsic link between dietary factors and ASD at the genetic level, hoping to provide research ideas for the prevention and diagnosis of ASD and help improve dietary guidance for children with ASD.
	**METHODS**				
4	**Study design and data sources**		Present key elements of the study design early in the article. Consider including a table listing sources of data for all phases of the study. For each data source contributing to the analysis, describe the following:		
		a)	Setting: Describe the study design and the underlying population, if possible. Describe the setting, locations, and relevant dates, including periods of recruitment, exposure, follow-up, and data collection, when available.	3	Study design
		b)	Participants: Give the eligibility criteria, and the sources and methods of selection of participants. Report the sample size, and whether any power or sample size calculations were carried out prior to the main analysis	4,5	Data source
		c)	Describe measurement, quality control and selection of genetic variants	5,6	Selection of instrumental variables
		d)	For each exposure, outcome, and other relevant variables, describe methods of assessment and diagnostic criteria for diseases	2	Autism spectrum disorder (ASD) is a neurodevelopmental disease characterized by social and communication impairments, restricted interests, and repetitive behavior.
		e)	Provide details of ethics committee approval and participant informed consent, if relevant	3	As the data used is previously publicly available, no additional ethical approval is required.
5	**Assumptions**		Explicitly state the three core IV assumptions for the main analysis (relevance, independence and exclusion restriction) as well assumptions for any additional or	4	MR analysis requires three core assumptions to be satisfied

			sensitivity analysis		
6	**Statistical methods: main analysis**		Describe statistical methods and statistics used		
		a)	Describe how quantitative variables were handled in the analyses (i.e., scale, units, model)	6	if $P < 0.05$, heterogeneity was present, random-effects IVW was selected, when heterogeneity was acceptable; if $P \geq 0.05$, heterogeneity was not present, fixed-effects IVW was selected.
		b)	Describe how genetic variants were handled in the analyses and, if applicable, how their weights were selected	6	The weighted median method
		c)	Describe the MR estimator (e.g. two-stage least squares, Wald ratio) and related statistics. Detail the included covariates and, in case of two-sample MR, whether the same covariate set was used for adjustment in the two samples	3	A two-sample MR analysis was performed using a publicly available GWAS based extensive sample database.
		d)	Explain how missing data were addressed	No	
		e)	If applicable, indicate how multiple testing was addressed	Not applicable	
7	**Assessment of assumptions**		Describe any methods or prior knowledge used to assess the assumptions or justify their validity	No	
8	**Sensitivity analyses and additional analyses**		Describe any sensitivity analyses or additional analyses performed (e.g. comparison of effect estimates from different approaches, independent replication, bias analytic techniques, validation of instruments, simulations)	6	The leave-one-out analysis is used for sensitivity analysis to assess the stability of the results.
9	**Software and pre-registration**				
		a)	Name statistical software and package(s), including version and settings used	6	All analyses were performed using the "TwoSampleMR" (version 0.5.6) and "MR-PRESSO" packages in R software (version 4.2.1)
		b)	State whether the study protocol and details were pre-registered (as well as when and where)	No	
	**RESULTS**				
10	**Descriptive data**				
		a)	Report the numbers of individuals at each stage of included studies and reasons for exclusion. Consider use of a flow diagram	6	the final number of valid SNPs for MR analysis ranged from 6 to 69.

		b)	Report summary statistics for phenotypic exposure(s), outcome(s), and other relevant variables (e.g. means, SDs, proportions)	6	Basic information about these SNPs is presented in the Supplementary material (Basic information of SNPs).
		c)	If the data sources include meta-analyses of previous studies, provide the assessments of heterogeneity across these studies	Not applicable	
		d)	For two-sample MR:<br>i. Provide justification of the similarity of the genetic variant-exposure associations between the exposure and outcome samples<br>ii. Provide information on the number of individuals who overlap between the exposure and outcome studies	4	However, it should be noted that this dataset was not extracted from the UK Biobank (the outcome variables were from a different dataset to the exposed variables).
11	**Main results**				
		a)	Report the associations between genetic variant and exposure, and between genetic variant and outcome, preferably on an interpretable scale	6	Basic information about these SNPs is presented in the Supplementary material (Basic information of SNPs).
		b)	Report MR estimates of the relationship between exposure and outcome, and the measures of uncertainty from the MR analysis, on an interpretable scale, such as odds ratio or relative risk per SD difference	6-8	This study analyzed the causal relationship between 18 dietary factors and ASD.
		c)	If relevant, consider translating estimates of relative risk into absolute risk for a meaningful time period	6-8	OR
		d)	Consider plots to visualize results (e.g. forest plot, scatterplot of associations between genetic variants and outcome versus between genetic variants and exposure)	7-9	The visualization results and sensitivity analysis of the MR analysis can be found in the Supplementary material (Visualization results).
12	**Assessment of assumptions**				
		a)	Report the assessment of the validity of the assumptions	No	
		b)	Report any additional statistics (e.g., assessments of heterogeneity across genetic variants, such as I^2, Q statistic or E-value)	7-9	Cochran's Q test
13	**Sensitivity analyses and additional analyses**				
		a)	Report any sensitivity analyses to assess the robustness of the main results to violations of the assumptions	7,8	The visualization results and sensitivity analysis of the MR analysis can be found in the Supplementary material (Visualization results).

		b)	Report results from other sensitivity analyses or additional analyses	7,8	The visualization results and sensitivity analysis of the MR analysis can be found in the Supplementary material (Visualization results).
		c)	Report any assessment of direction of causal relationship (e.g., bidirectional MR)	No	
		d)	When relevant, report and compare with estimates from non-MR analyses	No	
		e)	Consider additional plots to visualize results (e.g., leave-one-out analyses)	7,8	The visualization results and sensitivity analysis of the MR analysis can be found in the Supplementary material (Visualization results).
	**DISCUSSION**				
14	**Key results**		Summarize key results with reference to study objectives	10,11	The results indicated that the risk of ASD
15	**Limitations**		Discuss limitations of the study, taking into account the validity of the IV assumptions, other sources of potential bias, and imprecision. Discuss both direction and magnitude of any potential bias and any efforts to address them	12	This study has several limitations that warrant consideration.
16	**Interpretation**				
		a)	Meaning: Give a cautious overall interpretation of results in the context of their limitations and in comparison with other studies	12	In summary, this study revealed
		b)	Mechanism: Discuss underlying biological mechanisms that could drive a potential causal relationship between the investigated exposure and the outcome, and whether the gene-environment equivalence assumption is reasonable. Use causal language carefully, clarifying that IV estimates may provide causal effects only under certain assumptions	11,12	Therefore, we posit that cheese intake is highly likely to be a risk factor for ASD.
		c)	Clinical relevance: Discuss whether the results have clinical or public policy relevance, and to what extent they inform effect sizes of possible interventions	12	These findings provide a foundation for reliable clinical nutritional interventions for children with ASD, contributing to primary prevention strategies for ASD.
17	**Generalizability**		Discuss the generalizability of the study results (a) to other populations, (b) across other exposure periods/timings, and (c) across other levels of exposure	12	the study population consisted of individuals of European ancestry, which may limit the generalizability of the findings to other populations.
	**OTHER INFORMATION**				
18	**Funding**		Describe sources of funding and the role of funders in the present study and, if applicable, sources of funding for the databases and original study or studies on which the present study is based	12	This work was financially supported by Natural Science Foundation of Shandong Province (No. ZR2019MG022).

19	**Data and data sharing**	Provide the data used to perform all analyses or report where and how the data can be accessed, and reference these sources in the article. Provide the statistical code needed to reproduce the results in the article, or report whether the code is publicly accessible and if so, where	12	Data availability statement
20	**Conflicts of Interest**	All authors should declare all potential conflicts of interest	12	Conflict of interest

This checklist is copyrighted by the Equator Network under the Creative Commons Attribution 3.0 Unported (CC BY 3.0) license.

1. Skrivankova VW, Richmond RC, Woolf BAR, Yarmolinsky J, Davies NM, Swanson SA, et al. Strengthening the Reporting of Observational Studies in Epidemiology using Mendelian Randomization (STROBE-MR) Statement. JAMA. 2021;under review.

2. Skrivankova VW, Richmond RC, Woolf BAR, Davies NM, Swanson SA, VanderWeele TJ, et al. Strengthening the Reporting of Observational Studies in Epidemiology using Mendelian Randomisation (STROBE-MR): Explanation and Elaboration. BMJ. 2021;375:n2233.
